# DASPfind: new efficient method to predict drug–target interactions

**DOI:** 10.1186/s13321-016-0128-4

**Published:** 2016-03-16

**Authors:** Wail Ba-alawi, Othman Soufan, Magbubah Essack, Panos Kalnis, Vladimir B. Bajic

**Affiliations:** Computational Bioscience Research Center (CBRC), King Abdullah University of Science and Technology (KAUST), Thuwal, 23955-6900 Saudi Arabia; Computer, Electrical and Mathematical Sciences and Engineering Division (CEMSE), Infocloud Group, King Abdullah University of Science and Technology (KAUST), Thuwal, 23955-6900 Saudi Arabia

## Abstract

**Background:**

Identification of novel drug–target interactions (DTIs) is important for drug discovery. Experimental determination of such DTIs is costly and time consuming, hence it necessitates the development of efficient computational methods for the accurate prediction of potential DTIs. To-date, many computational methods have been proposed for this purpose, but they suffer the drawback of a high rate of false positive predictions.

**Results:**

Here, we developed a novel computational DTI prediction method, DASPfind. DASPfind uses simple paths of particular lengths inferred from a graph that describes DTIs, similarities between drugs, and similarities between the protein targets of drugs. We show that on average, over the four gold standard DTI datasets, DASPfind significantly outperforms other existing methods when the single top-ranked predictions are considered, resulting in 46.17 % of these predictions being correct, and it achieves 49.22 % correct single top ranked predictions when the set of all DTIs for a single drug is tested. Furthermore, we demonstrate that our method is best suited for predicting DTIs in cases of drugs with no known targets or with few known targets. We also show the practical use of DASPfind by generating novel predictions for the Ion Channel dataset and validating them manually.

**Conclusions:**

DASPfind is a computational method for finding reliable new interactions between drugs and proteins. We show over six different DTI datasets that DASPfind outperforms other state-of-the-art methods when the single top-ranked predictions are considered, or when a drug with no known targets or with few known targets is considered. We illustrate the usefulness and practicality of DASPfind by predicting novel DTIs for the Ion Channel dataset. The validated predictions suggest that DASPfind can be used as an efficient method to identify correct DTIs, thus reducing the cost of necessary experimental verifications in the process of drug discovery. DASPfind can be accessed online at: http://www.cbrc.kaust.edu.sa/daspfind.

**Graphical abstract:**
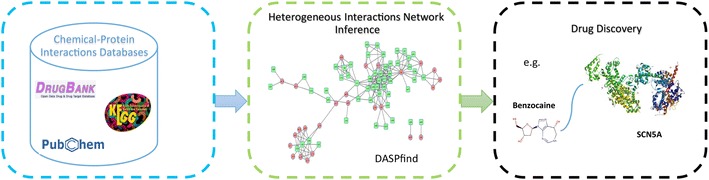
The conceptual workflow for predicting drug-target interactions using DASPfind

**Electronic supplementary material:**

The online version of this article (doi:10.1186/s13321-016-0128-4) contains supplementary material, which is available to authorized users.

## Background

Despite large research and development expenditures [1], only 27 new molecular entities were approved by the Food and Drug Administration (FDA) in 2013, illustrating the continued decline in drug discovery [2]. The approach to drug discovery based on *in silico* methods is thus becoming more attractive. Many efforts are put into developing methods for the prediction of drug–target interactions (DTIs) that mitigate the expensive and time consuming experimental identification of lead compounds and their interactors [3]. Moreover, such methods allow for the identification of potentially new therapeutic applications for the existing drugs (drug repositioning) that may reduce research cost and time due to the existing extensive clinical history and toxicology information of the drugs [4]. Furthermore, prediction of DTIs reveals drugs acting on multiple targets, i.e. those that exhibit polypharmacology, which may aid in understanding side effects caused by the use of drugs [5]. For example, one such *in silico* DTI prediction method [6] uses the crystal structure of the target binding site to yield a good prediction of druggability and to identify the less-druggable targets before the deployment of any substantial funding and effort for experiments. The study [6] further successfully and experimentally, tested two of the generated predictions using high-throughput screening of a diverse collection of compounds, thereby demonstrating the utility of their approach when dealing with difficult targets. Other studies, such as [7, 8], also successfully demonstrated the use of similar docking methods in identifying DTIs and in drug repositioning. The drawback of these docking methods is that they require high-resolution X-ray crystal (3D) structures of proteins, which are not known for membrane-bound proteins, that account for more than 40 % of current drug targets [9, 10]. An alternative ligand-based approach has therefore been developed based on the use of machine learning methods to predict the binding of a candidate ligand based on the known ligands of a target protein [11, 12]. One such ligand-based method to predict DTIs using a drug two-dimensional (2D) structural similarity is presented in [11], and is known as the similarity ensemble approach (SEA). The study experimentally confirmed 23 new DTIs, five of which were potent [13]. However, the performance of this ligand-based prediction method decreases as the number of known ligands of a particular target protein decreases. To further minimize the drawbacks of the above-mentioned methods, recently developed techniques have been based on supervised classification [4, 14–16] and graph interaction models [17]. In [17] the supervised inference method based on a bipartite graph is used to predict unknown DTIs. The study demonstrated the use of bipartite local models for generating two independent predictions: (a) prediction of target proteins of a given drug, and (b) prediction of drugs targeting a given protein. The obtained information is combined to give definitive predictions for each interaction. These predictions included known DTIs involving human enzymes, ion channels, G-protein-coupled receptors (GPCRs) and nuclear receptors, and also suggested potential novel DTIs [17]. On the other hand, [18] used a network-based inference (NBI) method that implements a simplified version of the algorithm proposed in [19]. The results clearly show good performance, but one limitation is that the knowledge pertaining to drug–drug similarities and target–target similarities has not been utilized, since only information from known drug–protein interactions has been used. Another limitation of NBI is its inability to give predictions for new drugs without known targets. The first limitation has been dealt with in [20] by adding information from the drug similarity and the targets similarity to the function used by NBI, which produced improved results. However, the resulting method (DT-Hybrid) still does not resolve the second limitation related to the target predictions for new drugs without known targets. The method, denoted as HGBI, presented in [21], also added a drug–similarity graph and a protein–similarity graph to the interactions graph used by NBI. This method allowed reducing both limitations of NBI. However, it used a restricted way of traversing the resultant network, so only partial information from the graph topology has been utilized and only partial benefits were achieved. Another approach that deals with both limitations is NRWRH [22], which uses a method of network-based random walk with a restart applied to a heterogeneous network.

In this study, we propose a novel method (DASPfind) that relies on the graph interaction model. Our method uses a heterogeneous graph consisting of three sub-graphs connected to each other. These sub-graphs represent: drug–drug similarity, protein–protein similarity, and known drug–protein interactions. Our algorithm for predicting new drug–protein interactions is based on all simple paths of particular lengths on such a graph model. The main idea in our method is to utilize the similarity information within the sub networks and combine it with information from the topology of the heterogeneous graph. In the results, we predict DTIs (targets here are proteins from several groups), and show that our method is capable of correctly predicting individual DTI in 27–53 % of cases (depending on the dataset and the target protein group) using only the single top-ranked prediction for a drug, achieving on average the correct prediction in 46.17 % of cases across the four gold standard datasets. Moreover, on the same datasets, the single top ranked DTI predictions by DASPfind are correct on average in 49.22 % of cases when predicting any of the known DTIs for a single drug, assuming there are no known DTIs for the drug. This last scenario corresponds to the case of predicting DTIs for a new drug without known targets. These results significantly outperform those produced by the other state-of-the-art methods. The notable advantage of DASPfind is demonstrated when considering the single top-ranked predictions and when there are no known or when there are very few known targets for a drug. We verified the utility of our method by providing a list of new predictions (not present in our datasets) several of which had been experimentally confirmed in other studies.

## Results and discussion

### Performance evaluation

To measure the performance of our method we compared it with the method reported in [22], denoted as NRWRH, the method reported in [20] denoted as DT-Hybrid, and the method reported in [21] denoted as HGBI. HGBI and DT-Hybrid, to the best of our knowledge, is the most recent works that have shown to outperform other state-of-the-art methods such as NBI [18] and BLM [17]. HGBI was demonstrated to perform better than NBI and BLM in terms of AUC and the ‘top 1’% of the predictions based on the leave-one-out-cross-validation (LOOCV). For our comparison, we also adopted the LOOCV scheme. The following procedure is repeated for each known DTI. For a drug, a single known DTI was removed from the graph and treated as a testing link. We made predictions of DTIs for that drug and all targets, and ranked in descending order the prediction scores generated. For each specific score threshold, if the testing score of the link is above the threshold, it is considered as true positive, and if the score of an unknown interaction is found above the threshold, it is considered as false positive. By varying the thresholds, we calculated true positive rate (TPR) and false positive rate (FPR) and hence generate the ROC curve. We then use the area under the curve (AUC) to show the overall performance of the method. More practically, we also counted the cases where the correct predictions were among the ‘top 5’, ‘top 2’ or represent the single top-ranked prediction (‘top 1’). ‘Top 5’ means that the link under the test is found among the ‘top 5’ predictions for that specific drug and so we report how many known interactions were found among these ‘top 5’. The same applies to the ‘top 2’ and ‘top 1’ prediction. Overall, the single top-ranked (‘top 1’) predictions are important for the utility of the method, as the aim is to find reliable predictions that can significantly reduce the set of required validation experiments.

### Performance

We used the four gold standard datasets of DTIs reported as benchmark data by many studies [14, 17, 18, 23]. To these four datasets, we added the set of approved drugs from DrugBank [24, 25] with their targets. For the purpose of fair comparison with HGBI, we added the dataset used by that method. Table 1 shows a summary of results when applying NRWRH, DT-Hybrid, HGBI and our DASPfind method to all benchmarking datasets. As shown in Table 1, the AUC values on most of the datasets appear to be similar for the four methods. The AUC information, however, is not of a great practical value in the context of narrowing down the number of candidate DTIs for downstream evaluation. The more useful appears to be the assessment of the correct DTI prediction from the top-ranked predictions. Different criteria have been used [20–22] to determine true positive predictions. We opted to use as the correct prediction, only the best one (the single top-ranked prediction from the ranked set of predictions for an individual drug, i.e. ‘top 1’ prediction), since such predictions will be ranked above all known DTIs for the drug, thus suggesting the highest confidence in such predictions. Two other customary ways would be to count a predicted DTI for a drug as correct, if it is top-ranked after removing known DTIs for that drug as used in [22], or to count a DTI prediction as correct if it is within the ‘top 1’% (5 or 10 %) of all predictions for the drug. In both of these cases it frequently happens that the predicted DTI is ranked below the known DTIs for the drug, thus confidence for such predictions is essentially smaller than by choosing the single top-ranked prediction for the drug. DASPfind outperformed the other methods significantly with respect to retrieving known DTIs as the single top-ranked (‘top 1’) predictions. For example, when considering only the ‘top 1’ DTI predictions found by DASPfind, based on the Enzyme target dataset in LOOCV, 52.08 % of these predictions were correct (known interactions), while this was the case for only 1.06 % of NRWRH, 2.36 % of HGBI and 0 % of DT-Hybrid. For the same dataset, in LOOCV, 62.74 % of the known interactions were retrieved among the ‘top 5’ DTI predictions by DASPfind, while among the ‘top 5’ DTI predictions by NRWRH, HGBI and DT-Hybrid only 12.89, 12.41, and 10.95 %, respectively, were the known ones. DASPfind outperformed the other methods we evaluated with respect to retrieving known interaction as the ‘top 1’ DTI predictions using all other datasets. On average, in the scenario when the ‘top 1’ ranked prediction of DTIs are considered in LOOCV, DASPfind makes a correct prediction in 46.17 % of cases across the four gold standard datasets and in 40.13 % across all six datasets we used. Overall, our results demonstrate that in this regard DASPfind is significantly superior to NRWRH, HGBI and DT-Hybrid. We also applied the tenfold cross-validation for the same scenario, which resulted in similar results (Additional file 1: Table S1).

**Table 1:** Comparison between methods over six different datasets based on LOOCV for each known DTI

Method	AUC (%)	‘Top 1’ (%)	‘Top 2’ (%)	‘Top 5’ (%)
Enzyme				
NRWRH	92.89	1.06	8.07	12.82
HGBI	91.60	2.36	8.1	12.41
DT-Hybrid	89.80	0	7.55	10.95
DASPfind	92.91	52.08	55.29	62.74
Ion channels				
NRWRH	91.56	1.69	2.91	10.16
HGBI	88.93	1.42	2.24	6.1
DT-Hybrid	92	0	1.42	14.3
DASPfind	90.68	32.72	35.09	46.54
GPCR				
NRWRH	84.93	2.52	11.50	40.94
HGBI	91.29	5.83	12.28	31.5
DT-Hybrid	83.87	0	6.93	31.65
DASPfind	88.10	46.61	51.18	64.4
Nuclear receptors				
NRWRH	73.9	7.78	32.22	52.22
HGBI	87.57	15.56	42.22	57.78
DT-Hybrid	69.95	0	14.44	22.22
DASPfind	85.27	53.3	65.5	77.7
HGBI_Dataset				
NRWRH	86.19	0	5.9	20.47
HGBI	89.07	0	5.17	16.19
DT-Hybrid	86.75	0	5.74	21.04
DASPfind	89.61	28.30	33.32	42.51
DrugBank_Approved				
NRWRH	89.5	1.04	5.65	18.63
HGBI	80.10	2.11	4.36	11.32
DT-Hybrid	84.44	0.34	5.88	22.69
DASPfind	88.84	27.82	32.89	48.56

We performed another experiment using LOOCV, each time removing for a drug all its known interactions and attempting to retrieve any of them as the ‘top 1’ prediction. This is equivalent to assessing the ability to predict a correct DTI for a new drug for which no DTIs are known. This scenario will generate a ranked list of DTI predictions for each drug like in the previous experiment. The difference here is that, in this case, we remove all known DTIs for the drug, while in the previous experiment we were removing individual DTIs. Table 2 shows the results for this new experiment between NRWRH, HGBI and DASPfind. We do not include the DT-Hybrid here, as it cannot produce predictions for drugs that are without known DTIs. On average, across the four gold standard datasets, the ‘top 1’ DTI predictions by DASPfind are correct in 49.22 % of cases, demonstrating that DASPfind performs better than NRWRH and HGBI, which achieve 27.26 and 41.94 %, respectively. In the same setup, across all six datasets, DASPfind, NRWRH, and HGBI make on average 42.34, 20.32, and 33.93 %, respectively, correct ‘top 1’ predictions. This means that in this setting DASPfind performs 2.084-fold and 1.248-fold better that NRWRH and HGBI, respectively. Note that in this experiment, a true positive would be any of the removed known DTIs between the drug and its targets, which increases the chances to get one of these removed links as the ‘top 1’ prediction.

**Table 2:** Comparison between methods over six different datasets based on LOOCV for each drug, assuming no DTIs are known for each drug. This is equivalent to estimating capacity to predict DTIs for new drugs without known targets

Data	Method	‘Top 1’ (%)
Enzyme	NRWRH	18.65
	HGBI	43.6
	DASPfind	49.66
Ion channels	NRWRH	33.33
	HGBI	35.71
	DASPfind	44.28
GPCR	NRWRH	25.56
	HGBI	42.15
	DASPfind	51.12
Nuclear receptors	NRWRH	31.48
	HGBI	46.30
	DASPfind	51.85
HGBI_Dataset	NRWRH	5.04
	HGBI	11.36
	DASPfind	11.49
DrugBank_Approved	NRWRH	7.9
	HGBI	24.49
	DASPfind	45.69

To complement our performance comparison study of different methods, we also used the same criterion as in [22] for NRWRH, where the predicted DTI is considered correct if it appears to be the top-ranked prediction after the removal of all predicted known DTIs. We demonstrate that in this case too, our method outperforms NRWRH when the targets for a drug are not known, or when there are only a few known targets of the drug (Additional file 1: Table S2, Figure S1).

In our study, we utilized information representing chemical similarity and protein similarity in addition to the information of the drug–protein interactions. In future, we plan to add different types of information, like drug side effects [26] and information derived from integrating multiple biological databases [27]. The complete lists of ‘top 1’ ranked predictions across all six datasets we used are provided in additional files (Additional file 1: Tables S3–S8).

### New predictions

To illustrate the utility of DASPfind, we applied it to the Ion Channel dataset without removing any of the known DTIs and attempted to predict new DTIs. Our method for this dataset generates 210 predictions as the ‘top 1’ DTI predictions (one for each drug). Out of these 210 DTI predictions, 91 are already known (the dataset we used has not been updated since 2008), while the remaining predicted DTIs are not present in that dataset. From the remaining 119 DTIs, which are from the viewpoint of our method new predictions, we selected 15 (Table 3) with the highest prediction score and evaluated them manually. We searched online resources (DrugBank [25], KEGG [28], SuperTarget [29], PubChem [30], GeneCards [31] and literature) to verify our predictions. We found that 12 out of these 15 do exist in the above-mentioned online resources indicating an interaction between the corresponding drugs and targets. We further investigated these 12 predictions as drug databases may contain noise. We found that 4 DTIs out of the 12 (pairs 2, 5, 12, and 13 in Table 3) are explicitly confirmed through experiments as reported in the literature (marked as italics in Table 3). For example, [32] studied the mechanosensitivity of SCN5A also known as NaV1.5, because mutations in NaV1.5 can result in disorders such as long QT type 3 (LQT3) in the heart and some pathologies in the gastrointestinal tract. Their experiments revealed that Benzocaine (pair 2, Table 3) could modulate NaV1.5 mechanosensitivity. Another 5 pairs from the list (pairs 1, 6, 9, 10, and 15 in Table 3) have targets that are related to sodium channels. These DTIs were reported explicitly in databases like DrugBank, KEGG, ZINC [33], Matador [29], and Drug2Gene [34]. However, we did not find in the references listed in these databases as support for the DTIs in question, that these DTIs are explicitly experimentally confirmed. Instead, we found information about these drugs to be typical drugs affecting the voltage-gated sodium channels in general. For example, Cocaine (pair 10, Table 3) is well known and one of the first local anesthetic drugs (LA) that has a confirmed effect to block sodium channel gates. Because of its severe side effects, clinical studies were conducted with similar drugs, but with less side effects. That led to a group of LAs with the ‘–caine’ suffix, such as dibucaine (pair 9, Table 3). Same observation applies to four other pairs (pair 4, 8, 11, and 14 in Table 3), which describe targets that belong to L-type calcium channels. Nimodipine (pair 4, Table 3) is a well-known drug for L-type calcium channels in general. Diltiazem hydrochloride (pair 11, Table 3) was reported to interact with the corresponding target in SuperTarget, which is a manually curated database, but without a link to a reference. We did not find any public information that validates pairs 3, 7, or 15. However, that does not imply that any of these are false. Overall, the results suggest that our method does provide reliable predictions useful for further downstream analyses.

**Table 3:** Fifteen novel ‘top 1’ predictions over the whole ion channel dataset

Pair	chemID (KEGG)	chemName	protID (KEGG)	protName	Score	Evidence	Type of evidence
*1*	D00538	Zonisamide	6331	SCN5A	177.46	[KEGG: D00538, PMID: 20025128]	Inferred
*2*	*D00552*	*Benzocaine*	*6331*	*SCN5A*	145.43	*[KEGG: D00552, PMID: 22874086]*	*Direct*
*3*	D00294	Diazoxide	6328	SCN3A	119	NA	
*4*	D00438	Nimodipine	779	CACNA1S	79.37	[DrugBank: DB00393, PMID: 17705883]	Inferred
*5*	*D03365*	*Nicotine*	*1137*	*CHRNA4*	72.05	*[PMID: 20061993]*	*Direct*
*6*	D00649	Amiloride hydrochloride	55800	SCN3B	67	Matador (http://matador.embl.de/proteins/9606.ENSP00000299333/)	Inferred
*7*	D00648	Ibutilide fumarate	779	CACNA1S	52.08	NA	
*8*	D05024	Mibefradil dihydrochloride	775	CACNA1C	52	[GeneCards: CACNA1C, PMID: 16306443]	Inferred
9	D00733	Dibucaine	6328	SCN3A	50.22	[KEGG: D00733, Drug2Gene: 103927525]	Inferred
*10*	D00110	Cocaine	6328	SCN3A	50.21	[KEGG: hsa6328, PMID: 22185904]	Inferred
*11*	D00616	Diltiazem hydrochloride	776	CACNA1D	49.31	[SuperTarget: has776, PubChem: CID39186]	Inferred
*12*	*D03830*	*Diltiazem malate*	*776*	*CACNA1D*	49.31	*[KEGG: D03830, PMID: 17949410]*	*Direct*
*13*	*D00619*	*Verapamil hydrochloride*	*778*	*CACNA1F*	41.25	*[DrugBank: DB00661], unpublished data (* http://edoc.ub.uni-muenchen.de/5321/ *)*	*Direct*
*14*	D01108	Magnesium sulfate	779	CACNA1S	38.08	[DrugBank: DB00653]	Inferred
*15*	D01969	Gallopamil hydrochloride	778	CACNA1F	34.07	NA	

## Conclusions

Our study introduces a method (DASPfind) that infers drug–protein interactions from a heterogeneous graph accompanied with information about similarities between drugs and similarities between targets. DASPfind relies on finding all simple paths of a specific length between any drug–protein pair, efficiently utilizing the drug similarity and protein similarity information and topology of the graph than the other current methods. We show that our method is significantly more accurate than the other state-of-the-art approaches when the single top-ranked DTI predictions are considered, as well as for the new drugs without known DTIs or for drugs with only a few known DTIs. These make DASPfind important and relevant for practical use. We show that our method is able to reliably predict novel DTIs with very high confidence, and validation of these DTIs proved DASPfind’s utility.

## Methods

### Experimental datasets

We used the gold standard datasets as collected by [23]. Each of the gold standard datasets represents one of four major families of drug targets, namely enzymes, ion channels, GPCRs, and nuclear receptors, and we refer to them using these names. As reported in [23], known interactions were extracted from the KEGG BRITE [35], BRENDA [36], SuperTarget [29] and DrugBank databases [25]. Chemical structures of the drugs were extracted from the KEGG LIGAND database [35], while the similarities between drugs were computed using SIMCOMP [37]. Similarity scores between the targets were computed using a normalized version of the Smith–Waterman algorithm [38]. Note that these datasets were compiled in 2008 and have not been updated since that time. We also added another dataset that we named DrugBank_approved, which contains all the FDA approved drugs in the DrugBank database along with their corresponding protein targets. We calculated similarities between drugs and between proteins in the same way as for the four gold standard datasets. In addition to that, we added the dataset used by HGBI for fair comparison. Table 4 shows a summary of these datasets. All datasets used in this study are available in additional files (Additional files 2, 3).

**Table 4:** Summary of the datasets used in this study

Dataset	Drugs	Target proteins	Known interactions
Enzyme	445	664	2926
Ion channels	210	204	1476
GPCR	223	95	635
Nuclear receptors	54	26	90
DrugBank_approved	1556	1610	5877
HGBI_Dataset	1409	4063	1915

### Methods

**Fig. 1:**
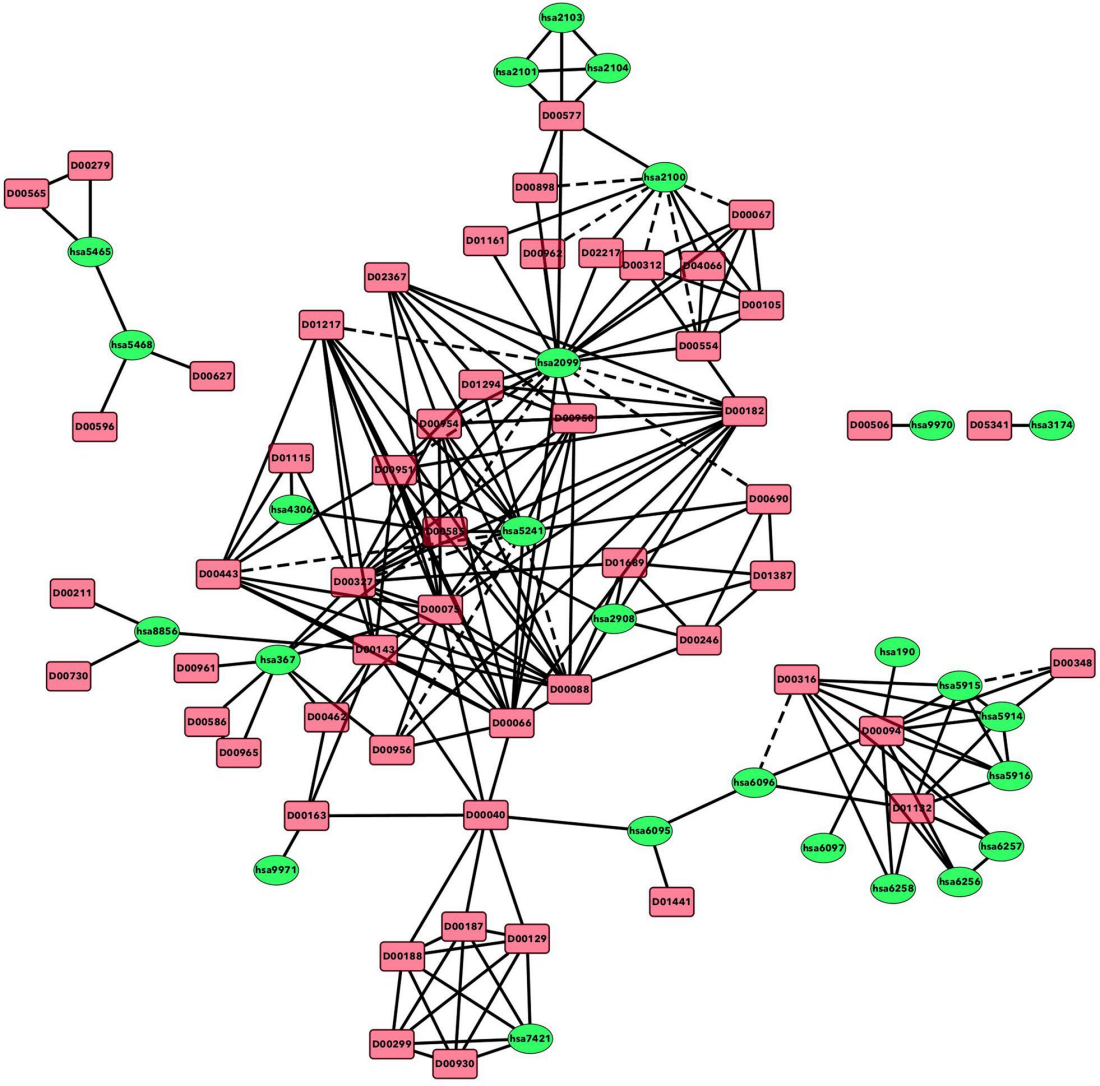
The heterogeneous graph built from the nuclear receptor dataset. Nodes represent drugs and proteins. Edges between drugs and proteins represent known interactions and are shown in *solid lines*. Edges between drugs alone or between proteins alone represent the similarity between them and are also represented by *solid lines*. *Dashed edges* represent predicted potentially new interactions

We consider a set of drugs ${\text{C}} = \{ {\text{c}}1,{\text{c}}2, \ldots \}$ and a set of proteins ${\text{T}} = \{ {\text{t}}1,{\text{t}}2, \ldots \} .$ A graph is constructed with nodes from C and T. The weight of an edge between two drug nodes represents the similarity between them and the weight of an edge between protein nodes represents similarity between the linked proteins. The degree of similarity is a score between (0, 1]. In our case, if the similarity between the nodes is smaller than 0.5 we do not show such links in the graph. An edge between a drug and a protein represents a known interaction between them with the weight of 1. Figure 1 shows an example of such a graph representing the nuclear receptor dataset, which is the smallest of our benchmark datasets.

**Fig. 2:**
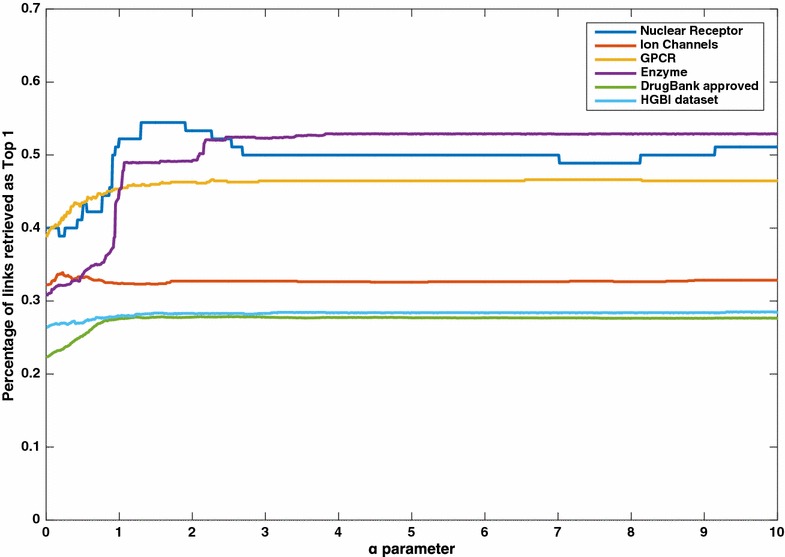
Results of changing α parameter across different datasets

In our method we traverse all simple paths between a drug and a target protein. A simple path means a path where nodes are not repeated so there are no cycles along the path. We do this by using a modification of a classical traversal algorithm like depth-first search to keep track of the visited nodes. The depth-first search is implemented as a recursive function that traverses the graph moving along the edge. We modify it to mark the nodes as they are visited in the recursion, and then remove the mark just before returning from the recursive call. This way we ensure no nodes are visited more than once in a specific path. We have tested our approach with different lengths and found that the length of up to three edges is the most suitable. Thus, for the application in this study we considered only paths up to the length three (although the method is not restricted to this length), i.e. a path should not have more than three edges. This constraint also significantly decreases the time required to find these paths. The assumption behind our method is that a drug and a protein have a higher probability to interact if there are more paths connecting them as the paths represent confident relationships between the nodes. Since the paths can vary in lengths (up to three edges long), we believe that, in general, longer paths should have less contribution to the aggregated score that represents the confidence of an interaction occurring between the end nodes of the path. So, we introduce an exponential decay function that gives less support for paths as the length of the path increases. Equation (1), defines how we aggregate the score from these different paths:

1 $${\text{score}} = \mathop \sum \limits_{{{\text{p}} = 1}}^{\text{n}} \left( {\prod {\text{p}}_{\text{w}} } \right)^{{\upalpha \times {\text{len(p)}}}}$$

where ${\text{p}} = \{ {\text{p}}_{1} ,{\text{p}}_{2} , \ldots ,{\text{p}}_{\text{n}} \}$ is the set of the paths connecting a specific drug and a specific protein, p_w_ is the weights of the edges along an individual path, and len(p) is the number of edges of that path; α is a parameter that we choose here to be equal to 2.26 as we found this value maximizes the average result across different benchmark datasets. However, it is possible to assign different values to each dataset for somewhat better results. Figure 2 shows the effect of changing this parameter across different datasets. For a path between a drug and its target we multiply the edges weights along the path. After that, we do the same for all other paths between this drug–target pair and aggregate the score through summation. We show the pseudocode for the DASPfind algorithm in additional files (Additional file 1: Figure S1). DASPfind can be accessed online at: http://www.cbrc.kaust.edu.sa/daspfind.

Our method differs from other methods in that it uses, in a specific manner not utilized by other methods, the similarities between drugs and between proteins along with the topology of the heterogeneous graph. For example, our method can utilize the following path over the network: drug1 (start) → protein1 → drug2 → protein2 (end), which maps to the following example path in the bottom-right side of Fig. 1: D00316 → hsa5915 → D00094 → hsa6096. Such a path gives the additional information that drugs interacting with the same target have some degree of similarity between them. Also, in most of the studies utilizing the network structure, all paths over the network contribute equally to the score, while we apply a decay function so that longer paths would have a lower total score.

## Additional files

10.1186/s13321-016-0128-4 This file includes the following: a) Pseudocode of DASPfind algorithm; b) 10-fold cross validation for different methods; c) detailed comparison between NRWRH and DASPfind; d) all ‘top 1’ predictions for each data set used in our study.
10.1186/s13321-016-0128-4 This contains DTI data sets named as: Nuclear Receptor, GPCR, Ion Channels, Enzyme, and DrugBank_approved.
10.1186/s13321-016-0128-4 This contains HGBI data set.
