# Supplementary material for: DASPfind: new efficient method to predict drug–target interactions
Source: J Cheminform. 2016 Mar 16;8:15. doi: 10.1186/s13321-016-0128-4 (PMC4793623; doi:10.1186/s13321-016-0128-4)
Supplement: Supplementary file 1 — 10.1186/s13321-016-0128-4 This file includes the following: a) Pseudocode of DASPfind algorithm; b) 10-fold cross validation for different methods; c) detailed comparison between NRWRH and DASPfind; d) all ‘top 1’ predictions for each data set used in our study. [file 13321_2016_128_MOESM1_ESM.docx]

**DASPfind: New Efficient Method to Predict Drug-Target Interactions**

**Wail Ba-alawi^1^, Othman Soufan^1^, Magbubah Essack^1^, Panos Kalnis^2^ and Vladimir B. Bajic^1,*^**

^1^King Abdullah University of Science and Technology (KAUST), Computational Bioscience Research Center (CBRC), Thuwal 23955-6900, Saudi Arabia. ^2^King Abdullah University of Science and Technology (KAUST), Infocloud Group, Computer, Electrical and Mathematical Sciences and Engineering Division (CEMSE), Thuwal 23955-6900, Saudi Arabia.

**SUPPLEMENTARY MATERIAL**


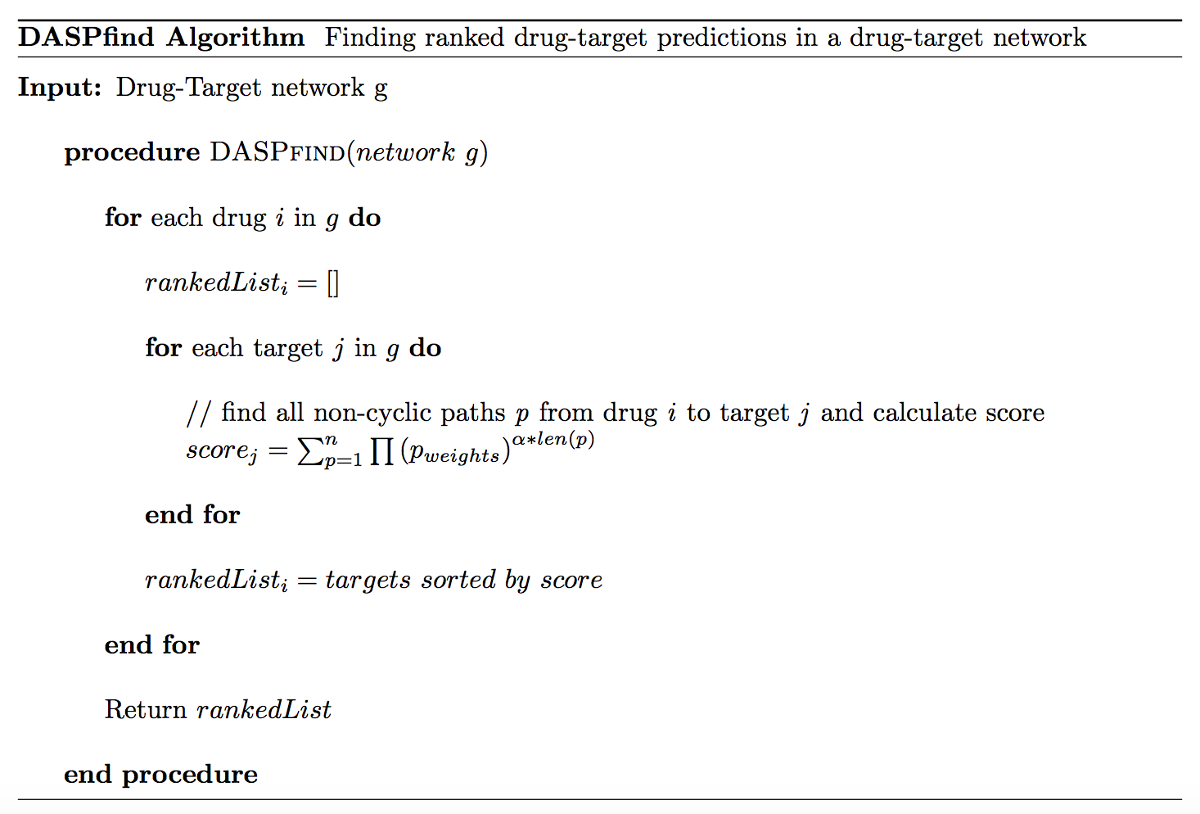


Figure S1. Pseudocode of DASPfind algorithm.

Table S1. Comparison between methods over six different datasets based on 10-fold cross-validation over all known interactions.

| **Enzyme** | | | |
| --- | --- | --- | --- |
| **Method** | **‘Top 1’ (%)** | **‘Top 2’ (%)** | **‘Top 5’ (%)** |
| NRWRH | **1.20** | 8.07 | 12.78 |
| HGBI | **2.50** | 7.79 | 12.17 |
| DT-Hybrid | **0** | 7.04 | 11.14 |
| DASPfind | **24.30** | 30.65 | 41.52 |
| **Ion Channels** | | | |
| **Method** | **‘Top 1’ (%)** | **‘Top 2’ (%)** | **‘Top 5’ (%)** |
| NRWRH | **1.49** | 3.18 | 10.57 |
| HGBI | **1.22** | 2.03 | 5.82 |
| DT-Hybrid | **0** | 1.35 | 13.68 |
| DASPfind | **23.50** | 29.67 | 43.50 |
| **GPCR** | | | |
| **Method** | **‘Top 1’ (%)** | **‘Top 2’ (%)** | **‘Top 5’ (%)** |
| NRWRH | **2.68** | 12.13 | 41.25 |
| HGBI | **5.68** | 12.92 | 31.04 |
| DT-Hybrid | **0** | 11.49 | 36.35 |
| DASPfind | **42.82** | 48.95 | 62.99 |
| **Nuclear Receptors** | | | |
| **Method** | **‘Top 1’ (%)** | **‘Top 2’ (%)** | **‘Top 5’ (%)** |
| NRWRH | **13.33** | 28.89 | 56.67 |
| HGBI | **14.44** | 38.89 | 56.67 |
| DT-Hybrid | **0** | 10.0 | 21.11 |
| DASPfind | **48.88** | 56.66 | 71.11 |
| **HGBI_Dataset** | | | |
| **Method** | **‘Top 1’ (%)** | **‘Top 2’ (%)** | **‘Top 5’ (%)** |
| NRWRH | **0.26** | 6.16 | 20.57 |
| HGBI | **0.21** | 5.12 | 15.67 |
| DT-Hybrid | **0** | 3.86 | 16.16 |
| DASPfind | **23.81** | 30.65 | 43.34 |
| **DrugBank_Approved** | | | |
| **Method** | **‘Top 1’ (%)** | **‘Top 2’ (%)** | **‘Top 5’ (%)** |
| NRWRH | **1.02** | 5.46 | 18.22 |
| HGBI | **2.06** | 4.47 | 11.88 |
| DT-Hybrid | **0.31** | 6.05 | 21.14 |
| DASPfind | **24.82** | 32.34 | 43.04 |

Table S2. Comparison between NRWRH and DASPfind over six different datasets based on LOOCV for each known interactions. Criterion to count the correct ‘Top N’ predictions is the same as in NRWRH.

| **Enzyme** | | | |
| --- | --- | --- | --- |
| **Method** | **‘Top 1’ (%)** | **‘Top 2’ (%)** | **‘Top 5’ (%)** |
| NRWH | 70.16 | 0.7884 | 0.8431 |
| DASPfind | 72.24 | 0.8082 | 0.8482 |
| **Ion Channels** | | | |
| **Method** | **‘Top 1’ (%)** | **‘Top 2’ (%)** | **‘Top 5’ (%)** |
| NRWH | 73.31 | 0.8178 | 0.8855 |
| DASPfind | 67.81 | 0.7411 | 0.7777 |
| **GPCR** | | | |
| **Method** | **‘Top 1’ (%)** | **‘Top 2’ (%)** | **‘Top 5’ (%)** |
| NRWH | 63.15 | 71.18 | 81.89 |
| DASPfind | 58.89 | 66.29 | 80.00 |
| **Nuclear Receptors** | | | |
| **Method** | **‘Top 1’ (%)** | **‘Top 2’ (%)** | **‘Top 5’ (%)** |
| NRWH | 50.00 | 64.44 | 80.00 |
| DASPfind | 58.88 | 72.22 | 83.33 |
| **HGBI_Dataset** | | | |
| **Method** | **‘Top 1’ (%)** | **‘Top 2’ (%)** | **‘Top 5’ (%)** |
| NRWH | 49.19 | 56.03 | 66.52 |
| DASPfind | 44.64 | 51.33 | 59.47 |
| **DrugBank_Approved** | | | |
| **Method** | **‘Top 1’ (%)** | **‘Top 2’ (%)** | **‘Top 5’ (%)** |
| NRWH | 47.01 | 53.61 | 64.06 |
| DASPfind | 44.69 | 51.79 | 59.50 |

**Figure S2. Comparison between NRWRH and DASPfind based on the ‘Top 1’ prediction using the criterion as in NRWRH in a LOOCV setup.** In this comparison, each value in the x-axis represents a network where each drug would have known interactions of at most that x value. Y-axis represents how many known interactions were retrieved from the ‘Top 1’ predictions for each constructed network.


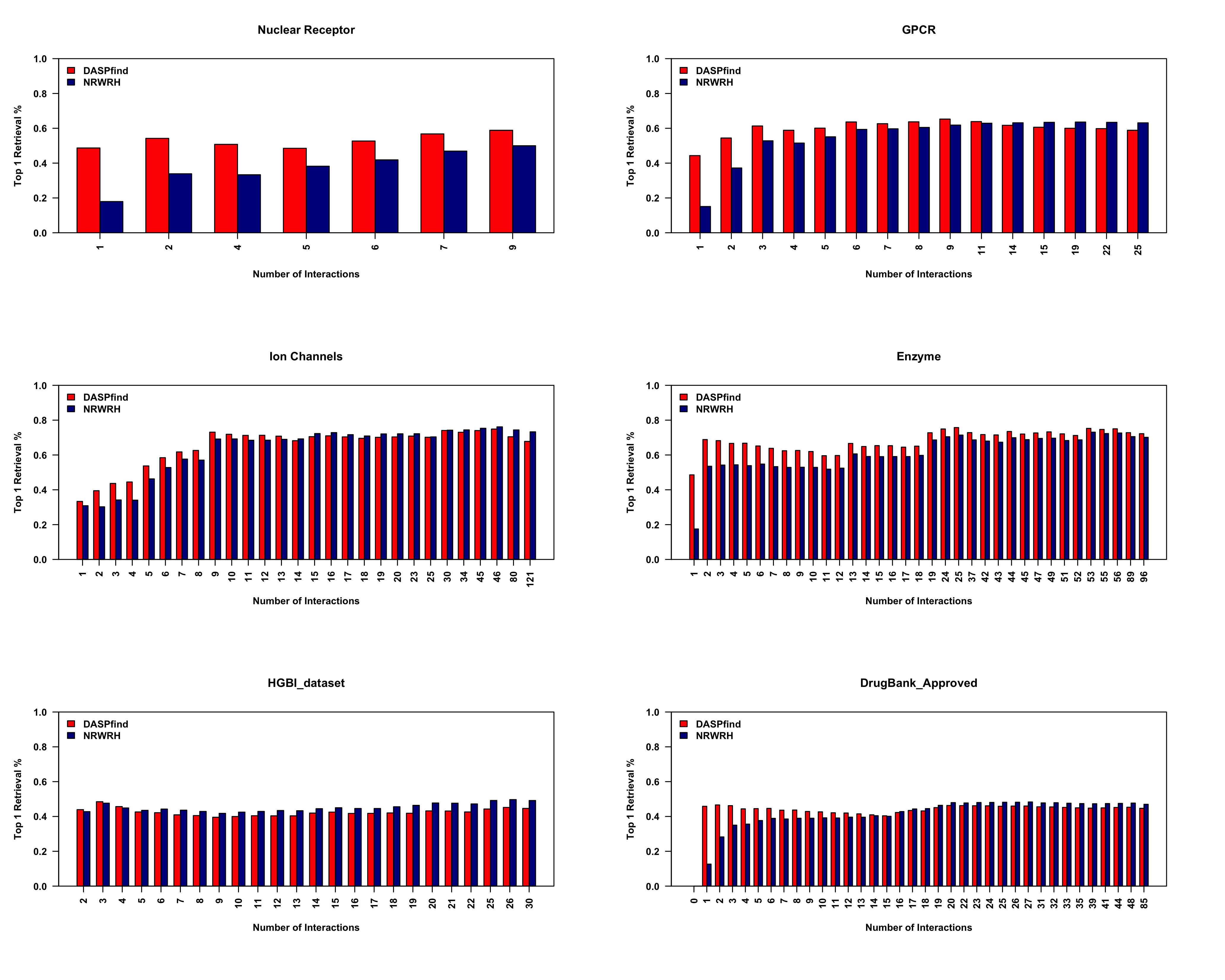


Table S3. ‘Top 1’ predictions for the Nuclear Receptor Dataset. ‘Exist’ refers to predictions of links that represent known DTIs in the network. Novel refers to new predictions that are not known DTIs in the network.

| **chemID (KEGG)** | **protID (KEGG)** | **Score** | **Type** |
| --- | --- | --- | --- |
| D00094 | hsa5914 | 13.453 | Exist |
| D01132 | hsa5915 | 12.483 | Exist |
| D00316 | hsa6096 | 12 | Novel |
| D00950 | hsa5241 | 8.064 | Exist |
| D00954 | hsa5241 | 8.064 | Exist |
| D00182 | hsa2099 | 7.724 | Novel |
| D00327 | hsa5241 | 6.649 | Novel |
| D02367 | hsa5241 | 6.4 | Exist |
| D00066 | hsa5241 | 6.281 | Exist |
| D00105 | hsa2099 | 6.183 | Exist |
| D00585 | hsa2099 | 6.059 | Novel |
| D01217 | hsa2099 | 5.622 | Novel |
| D01294 | hsa5241 | 5.619 | Exist |
| D00554 | hsa2100 | 5.539 | Novel |
| D00067 | hsa2100 | 5.519 | Novel |
| D00951 | hsa2099 | 5.441 | Novel |
| D00898 | hsa2100 | 5.404 | Novel |
| D00577 | hsa2099 | 5.404 | Exist |
| D04066 | hsa2099 | 5.289 | Exist |
| D00312 | hsa2100 | 5.145 | Novel |
| D00690 | hsa2099 | 5 | Novel |
| D00962 | hsa2100 | 5 | Novel |
| D01161 | hsa2099 | 5 | Exist |
| D02217 | hsa2099 | 5 | Exist |
| D00348 | hsa5915 | 4.472 | Novel |
| D00075 | hsa5241 | 2.969 | Novel |
| D00187 | hsa7421 | 2.122 | Exist |
| D00188 | hsa7421 | 2.001 | Exist |
| D00930 | hsa7421 | 1.907 | Exist |
| D00129 | hsa7421 | 1.902 | Exist |
| D00088 | hsa5241 | 1.875 | Novel |
| D00299 | hsa7421 | 1.772 | Exist |
| D00443 | hsa5241 | 1.483 | Novel |
| D00246 | hsa2908 | 1.336 | Exist |
| D01689 | hsa2908 | 1.208 | Exist |
| D01387 | hsa2908 | 1.198 | Exist |
| D01115 | hsa4306 | 1.182 | Exist |
| D00956 | hsa5241 | 1.136 | Novel |
| D00586 | hsa367 | 1.083 | Exist |
| D00965 | hsa367 | 1.083 | Exist |
| D00462 | hsa367 | 1.065 | Exist |
| D00279 | hsa5465 | 1.051 | Exist |
| D00565 | hsa5465 | 1.051 | Exist |
| D00040 | hsa6095 | 1 | Exist |
| D00143 | hsa8856 | 1 | Exist |
| D00163 | hsa9971 | 1 | Exist |
| D00211 | hsa8856 | 1 | Exist |
| D00506 | hsa9970 | 1 | Exist |
| D00596 | hsa5468 | 1 | Exist |
| D00627 | hsa5468 | 1 | Exist |
| D00730 | hsa8856 | 1 | Exist |
| D00961 | hsa2099 | 1 | Exist |
| D01441 | hsa6095 | 1 | Exist |
| D05341 | hsa3174 | 1 | Exist |

Table S4. ‘Top 1’ predictions for the GPCR Dataset. ‘Exist’ refers to predictions of links that represent known DTIs in the network. Novel refers to new predictions that are not known DTIs in the network.

| **chemID (KEGG)** | **protID (KEGG)** | **Score** | **Type** |
| --- | --- | --- | --- |
| D00454 | hsa148 | 138.201 | Exist |
| D00283 | hsa148 | 125.434 | Exist |
| D00513 | hsa148 | 117.008 | Exist |
| D00996 | hsa148 | 103.828 | Exist |
| D02149 | hsa148 | 103.161 | Exist |
| D00255 | hsa148 | 95 | Exist |
| D00437 | hsa148 | 95 | Exist |
| D01713 | hsa148 | 90.232 | Exist |
| D00281 | hsa146 | 88.732 | Exist |
| D00607 | hsa146 | 88.151 | Exist |
| D02237 | hsa146 | 88.151 | Exist |
| D00509 | hsa146 | 88 | Exist |
| D00609 | hsa146 | 88 | Exist |
| D01022 | hsa146 | 88 | Exist |
| D01603 | hsa146 | 88 | Exist |
| D02361 | hsa148 | 86.401 | Exist |
| D02356 | hsa146 | 85 | Exist |
| D02354 | hsa1128 | 67.677 | Exist |
| D00604 | hsa147 | 65.732 | Novel |
| D00494 | hsa1128 | 62.887 | Exist |
| D00726 | hsa3356 | 61 | Exist |
| D01051 | hsa150 | 58.231 | Novel |
| D01358 | hsa150 | 55.972 | Novel |
| D00426 | hsa148 | 55 | Exist |
| D00270 | hsa148 | 54.6 | Novel |
| D00613 | hsa146 | 49.056 | Novel |
| D01024 | hsa150 | 49.002 | Novel |
| D01020 | hsa150 | 49 | Novel |
| D01965 | hsa150 | 49 | Novel |
| D02234 | hsa150 | 49 | Novel |
| D00563 | hsa146 | 46.823 | Novel |
| D00493 | hsa1813 | 46.481 | Exist |
| D01390 | hsa153 | 44.81 | Exist |
| D02150 | hsa153 | 44.81 | Exist |
| D03274 | hsa146 | 44.001 | Novel |
| D00606 | hsa146 | 44 | Novel |
| D02076 | hsa146 | 44 | Novel |
| D04034 | hsa146 | 44 | Novel |
| D03879 | hsa154 | 43.733 | Exist |
| D00483 | hsa153 | 43.025 | Exist |
| D01454 | hsa154 | 42.519 | Exist |
| D00451 | hsa3351 | 42.422 | Exist |
| D02338 | hsa153 | 42.385 | Exist |
| D02066 | hsa154 | 42.345 | Exist |
| D00432 | hsa154 | 42.21 | Exist |
| D02374 | hsa154 | 42.053 | Exist |
| D03415 | hsa153 | 42 | Exist |
| D02070 | hsa1128 | 41.774 | Exist |
| D00113 | hsa1128 | 41.528 | Exist |
| D01871 | hsa1128 | 41.499 | Exist |
| D00095 | hsa155 | 39.653 | Novel |
| D00136 | hsa148 | 39.311 | Novel |
| D02671 | hsa1813 | 38.975 | Exist |
| D01295 | hsa3356 | 37.011 | Novel |
| D00503 | hsa3356 | 35.675 | Novel |
| D03621 | hsa1128 | 34.472 | Exist |
| D01164 | hsa3356 | 34.173 | Exist |
| D02910 | hsa154 | 34 | Novel |
| D00632 | hsa155 | 33.069 | Novel |
| D03881 | hsa155 | 32.353 | Novel |
| D00235 | hsa155 | 32.251 | Novel |
| D02342 | hsa155 | 32.131 | Novel |
| D03490 | hsa155 | 32.111 | Novel |
| D00598 | hsa155 | 32.087 | Novel |
| D03880 | hsa155 | 32.003 | Novel |
| D00601 | hsa155 | 32.001 | Novel |
| D00635 | hsa155 | 32 | Novel |
| D00645 | hsa155 | 32 | Novel |
| D00560 | hsa3356 | 30.108 | Novel |
| D02566 | hsa146 | 29.095 | Novel |
| D01973 | hsa3356 | 29.046 | Novel |
| D03858 | hsa1128 | 28.323 | Exist |
| D02358 | hsa154 | 28.308 | Novel |
| D02359 | hsa153 | 28.225 | Novel |
| D04625 | hsa154 | 28.218 | Novel |
| D02340 | hsa1812 | 28.103 | Novel |
| D00232 | hsa1128 | 28.064 | Exist |
| D02147 | hsa153 | 27.928 | Novel |
| D02614 | hsa154 | 27.478 | Novel |
| D00683 | hsa153 | 27.194 | Novel |
| D00688 | hsa153 | 27.151 | Novel |
| D01386 | hsa153 | 27.052 | Novel |
| D00397 | hsa1131 | 27 | Novel |
| D00684 | hsa153 | 27 | Novel |
| D00687 | hsa153 | 27 | Novel |
| D05792 | hsa153 | 27 | Novel |
| D04375 | hsa151 | 25.16 | Novel |
| D02327 | hsa1129 | 25.001 | Novel |
| D00332 | hsa151 | 25 | Novel |
| D00514 | hsa151 | 25 | Novel |
| D02349 | hsa151 | 25 | Novel |
| D01692 | hsa146 | 24.077 | Novel |
| D00954 | hsa146 | 24 | Novel |
| D00965 | hsa146 | 24 | Novel |
| D00110 | hsa1813 | 23 | Novel |
| D00274 | hsa1128 | 21.001 | Exist |
| D00540 | hsa1131 | 20.215 | Novel |
| D00646 | hsa1131 | 20.047 | Novel |
| D00465 | hsa1131 | 20.045 | Novel |
| D00779 | hsa1131 | 20 | Novel |
| D00524 | hsa1128 | 20 | Exist |
| D00790 | hsa1814 | 19.722 | Novel |
| D01118 | hsa1129 | 19.644 | Novel |
| D00715 | hsa1129 | 19.46 | Novel |
| D01297 | hsa1129 | 19.248 | Novel |
| D01103 | hsa1129 | 19.247 | Novel |
| D00059 | hsa1814 | 19 | Novel |
| D00525 | hsa1129 | 19 | Novel |
| D00760 | hsa1128 | 19 | Novel |
| D00765 | hsa1128 | 19 | Novel |
| D01269 | hsa1129 | 19 | Novel |
| D00676 | hsa3351 | 15.34 | Exist |
| D01745 | hsa1812 | 12.515 | Novel |
| D00415 | hsa3355 | 12.086 | Novel |
| D00675 | hsa3355 | 12 | Novel |
| D00780 | hsa1812 | 12 | Novel |
| D00987 | hsa1812 | 12 | Novel |
| D01462 | hsa1812 | 12 | Novel |
| D02826 | hsa3355 | 12 | Novel |
| D03165 | hsa1812 | 12 | Novel |
| D05740 | hsa3355 | 12 | Novel |
| D00300 | hsa148 | 11.097 | Novel |
| D02357 | hsa1813 | 11.069 | Novel |
| D00234 | hsa148 | 11.011 | Novel |
| D00559 | hsa1813 | 11 | Novel |
| D03654 | hsa1128 | 10.067 | Novel |
| D01699 | hsa1128 | 10.062 | Novel |
| D00318 | hsa3269 | 10 | Novel |
| D02578 | hsa3356 | 9.003 | Novel |
| D00480 | hsa1813 | 8.88 | Novel |
| D04979 | hsa3356 | 8.275 | Novel |
| D01324 | hsa3356 | 7.764 | Novel |
| D00364 | hsa1813 | 7.334 | Novel |
| D01242 | hsa1128 | 7.26 | Novel |
| D00521 | hsa1813 | 7.192 | Novel |
| D00665 | hsa1128 | 7.165 | Novel |
| D01782 | hsa1128 | 7.117 | Novel |
| D00666 | hsa1128 | 7.09 | Novel |
| D00520 | hsa1128 | 7.006 | Novel |
| D01332 | hsa1128 | 7 | Novel |
| D01717 | hsa1128 | 7 | Novel |
| D05129 | hsa1128 | 7 | Novel |
| D00674 | hsa3351 | 6.136 | Novel |
| D00528 | hsa135 | 5.421 | Exist |
| D00227 | hsa134 | 5.059 | Exist |
| D00775 | hsa2918 | 4.865 | Exist |
| D03966 | hsa2914 | 4.546 | Novel |
| D02884 | hsa135 | 4.34 | Exist |
| D01712 | hsa135 | 4.328 | Exist |
| D01994 | hsa3350 | 4 | Novel |
| D06056 | hsa3350 | 4 | Novel |
| D06396 | hsa3350 | 4 | Novel |
| D05312 | hsa4988 | 3.378 | Exist |
| D05113 | hsa4986 | 3.297 | Novel |
| D00498 | hsa4986 | 3.148 | Novel |
| D00838 | hsa4988 | 3.147 | Exist |
| D00845 | hsa4986 | 3.076 | Novel |
| D05938 | hsa4986 | 3.076 | Novel |
| D00301 | hsa4986 | 3.075 | Novel |
| D04716 | hsa4986 | 3.075 | Novel |
| D00837 | hsa4986 | 3.075 | Exist |
| D00682 | hsa5739 | 2.876 | Novel |
| D01352 | hsa5731 | 2.779 | Novel |
| D00079 | hsa5731 | 2.748 | Novel |
| D00180 | hsa5739 | 2.654 | Exist |
| D00422 | hsa3274 | 2.438 | Exist |
| D00673 | hsa3274 | 2.414 | Exist |
| D01346 | hsa2916 | 2.226 | Novel |
| D02721 | hsa5739 | 2.137 | Exist |
| D00419 | hsa5731 | 2.129 | Novel |
| D00440 | hsa3274 | 2.119 | Exist |
| D02725 | hsa5739 | 2.047 | Novel |
| D00499 | hsa1128 | 2 | Novel |
| D01828 | hsa57105 | 2 | Novel |
| D03365 | hsa1128 | 2 | Novel |
| D00411 | hsa10800 | 2 | Exist |
| D00371 | hsa135 | 1.879 | Novel |
| D00295 | hsa3274 | 1.772 | Exist |
| D03503 | hsa3274 | 1.772 | Exist |
| D00394 | hsa148 | 1.555 | Novel |
| D00442 | hsa6755 | 1.55 | Novel |
| D02082 | hsa185 | 1.498 | Exist |
| D04040 | hsa185 | 1.498 | Exist |
| D05246 | hsa185 | 1.413 | Exist |
| D02588 | hsa7201 | 1.385 | Exist |
| D02279 | hsa222545 | 1.38 | Exist |
| D03187 | hsa5732 | 1.373 | Exist |
| D01925 | hsa7201 | 1.36 | Exist |
| D00522 | hsa185 | 1.339 | Exist |
| D00400 | hsa185 | 1.287 | Exist |
| D04006 | hsa136 | 1.279 | Exist |
| D01126 | hsa222545 | 1.269 | Exist |
| D02007 | hsa7201 | 1.222 | Exist |
| D02250 | hsa6752 | 1.218 | Exist |
| D02278 | hsa222545 | 1.211 | Exist |
| D00356 | hsa5737 | 1.19 | Exist |
| D01964 | hsa5737 | 1.19 | Exist |
| D01891 | hsa5731 | 1.142 | Exist |
| D00627 | hsa185 | 1.138 | Exist |
| D01227 | hsa1909 | 1.064 | Exist |
| D00523 | hsa185 | 1.055 | Exist |
| D00410 | hsa148 | 1 | Novel |
| D00049 | hsa338442 | 1 | Exist |
| D00094 | hsa9052 | 1 | Exist |
| D00106 | hsa10161 | 1 | Exist |
| D00139 | hsa10161 | 1 | Exist |
| D00225 | hsa10161 | 1 | Exist |
| D00241 | hsa2550 | 1 | Exist |
| D00306 | hsa1268 | 1 | Exist |
| D00336 | hsa6915 | 1 | Exist |
| D00380 | hsa10161 | 1 | Exist |
| D00443 | hsa185 | 1 | Exist |
| D00542 | hsa10161 | 1 | Exist |
| D00574 | hsa10161 | 1 | Exist |
| D00769 | hsa10161 | 1 | Exist |
| D01071 | hsa10161 | 1 | Exist |
| D01236 | hsa552 | 1 | Exist |
| D01441 | hsa9283 | 1 | Exist |
| D01652 | hsa5724 | 1 | Exist |
| D03210 | hsa1234 | 1 | Exist |
| D03442 | hsa886 | 1 | Exist |
| D03642 | hsa6915 | 1 | Exist |
| D05341 | hsa6010 | 1 | Exist |

Table S5. ‘Top 1’ predictions for the Ion Channels Dataset. ‘Exist’ refers to predictions of links that represent known DTIs in the network. Novel refers to new predictions that are not known DTIs in the network.

| **chemID (KEGG)** | **protID (KEGG)** | **Score** | **Type** |
| --- | --- | --- | --- |
| D02356 | hsa775 | 328.264 | Exist |
| D00332 | hsa8912 | 303 | Exist |
| D00512 | hsa8912 | 303 | Exist |
| D00629 | hsa775 | 274.029 | Exist |
| D00618 | hsa775 | 273.864 | Exist |
| D00437 | hsa775 | 273.773 | Exist |
| D00560 | hsa775 | 272.364 | Exist |
| D01295 | hsa775 | 272.364 | Exist |
| D00542 | hsa775 | 254.295 | Exist |
| D01450 | hsa6328 | 196.475 | Exist |
| D00740 | hsa6328 | 196.194 | Exist |
| D01287 | hsa6328 | 189.469 | Exist |
| D04048 | hsa6328 | 188.777 | Exist |
| D02086 | hsa6328 | 188.032 | Exist |
| D00738 | hsa6328 | 187.912 | Exist |
| D00358 | hsa6328 | 187.737 | Exist |
| D00732 | hsa6328 | 186.803 | Exist |
| D01243 | hsa6328 | 186.495 | Exist |
| D00642 | hsa6328 | 186.381 | Exist |
| D02272 | hsa6328 | 186.381 | Exist |
| D00735 | hsa6328 | 185.979 | Exist |
| D00741 | hsa6328 | 185.966 | Exist |
| D00739 | hsa6328 | 185.91 | Exist |
| D00547 | hsa2554 | 177.965 | Exist |
| D00538 | hsa6331 | 177.468 | Novel |
| D00545 | hsa2554 | 175.175 | Exist |
| D00293 | hsa2554 | 172.263 | Exist |
| D02098 | hsa6328 | 171.121 | Exist |
| D00549 | hsa2554 | 170.75 | Exist |
| D00640 | hsa6328 | 170.381 | Exist |
| D00499 | hsa2554 | 164.412 | Exist |
| D00638 | hsa6328 | 161.381 | Exist |
| D00530 | hsa2555 | 159.784 | Exist |
| D00225 | hsa2554 | 158.639 | Exist |
| D00550 | hsa2554 | 158.467 | Exist |
| D00329 | hsa2554 | 158.24 | Exist |
| D00548 | hsa2555 | 157.784 | Exist |
| D00775 | hsa6328 | 157.39 | Exist |
| D00252 | hsa6328 | 156.395 | Exist |
| D00303 | hsa6328 | 156.39 | Exist |
| D02088 | hsa6328 | 151.981 | Exist |
| D00477 | hsa6328 | 151.107 | Exist |
| D00639 | hsa6328 | 150.825 | Exist |
| D03991 | hsa6328 | 150.538 | Exist |
| D02087 | hsa6328 | 150.409 | Exist |
| D00354 | hsa6328 | 150.381 | Exist |
| D00552 | hsa6331 | 145.436 | Novel |
| D00349 | hsa775 | 119.323 | Exist |
| D00294 | hsa6328 | 119 | Novel |
| D02207 | hsa1135 | 100.398 | Exist |
| D02101 | hsa1136 | 94.45 | Exist |
| D03580 | hsa1136 | 94.45 | Exist |
| D00195 | hsa1136 | 94.302 | Exist |
| D02173 | hsa1136 | 94.302 | Exist |
| D00611 | hsa1136 | 94.194 | Exist |
| D02204 | hsa1136 | 94.194 | Exist |
| D03826 | hsa1136 | 94.194 | Exist |
| D05156 | hsa1136 | 94.194 | Exist |
| D05453 | hsa1136 | 94.194 | Exist |
| D00726 | hsa1136 | 92.194 | Exist |
| D00438 | hsa779 | 79.372 | Novel |
| D00331 | hsa2555 | 76.784 | Exist |
| D00711 | hsa2555 | 76.784 | Exist |
| D02253 | hsa2555 | 76.784 | Exist |
| D02461 | hsa2555 | 76.784 | Exist |
| D05461 | hsa2555 | 76.784 | Exist |
| D03365 | hsa1137 | 72.056 | Novel |
| D00524 | hsa1136 | 71.194 | Exist |
| D00649 | hsa55800 | 67 | Novel |
| D00617 | hsa775 | 62.076 | Exist |
| D00319 | hsa775 | 61.664 | Exist |
| D00615 | hsa775 | 60.561 | Exist |
| D02914 | hsa775 | 60.492 | Exist |
| D00648 | hsa779 | 52.085 | Novel |
| D05024 | hsa775 | 52 | Novel |
| D00733 | hsa6328 | 50.22 | Novel |
| D00110 | hsa6328 | 50.213 | Novel |
| D00616 | hsa776 | 49.315 | Novel |
| D03830 | hsa776 | 49.315 | Novel |
| D00619 | hsa778 | 41.252 | Novel |
| D01453 | hsa6263 | 39.648 | Exist |
| D01712 | hsa6263 | 39.065 | Exist |
| D02409 | hsa6263 | 38.691 | Exist |
| D00227 | hsa6263 | 38.37 | Exist |
| D01108 | hsa779 | 38.085 | Novel |
| D01969 | hsa778 | 34.074 | Novel |
| D00528 | hsa6263 | 32.577 | Exist |
| D06172 | hsa6328 | 31.736 | Novel |
| D01275 | hsa6263 | 31.37 | Exist |
| D01350 | hsa6263 | 31.37 | Exist |
| D00553 | hsa6328 | 31.364 | Novel |
| D05077 | hsa6328 | 30.798 | Novel |
| D02630 | hsa776 | 30.168 | Novel |
| D00539 | hsa773 | 30 | Novel |
| D00533 | hsa6328 | 29.442 | Novel |
| D00708 | hsa6328 | 29.133 | Novel |
| D00709 | hsa6328 | 28.628 | Novel |
| D00546 | hsa2555 | 25.742 | Novel |
| D00537 | hsa6328 | 25.628 | Novel |
| D00504 | hsa6328 | 24.628 | Novel |
| D00543 | hsa2555 | 24.55 | Novel |
| D00544 | hsa2555 | 24.304 | Novel |
| D01768 | hsa6328 | 22.505 | Novel |
| D00677 | hsa3359 | 21.89 | Exist |
| D00678 | hsa3359 | 21.561 | Exist |
| D02041 | hsa285242 | 21.368 | Exist |
| D00633 | hsa285242 | 21.301 | Exist |
| D00338 | hsa2555 | 16.957 | Novel |
| D01253 | hsa2555 | 16.84 | Novel |
| D03180 | hsa2555 | 16.794 | Novel |
| D00464 | hsa2555 | 16.783 | Novel |
| D06106 | hsa2555 | 16.453 | Novel |
| D00694 | hsa2555 | 16.446 | Novel |
| D04985 | hsa2555 | 16.397 | Novel |
| D00474 | hsa2555 | 16.284 | Novel |
| D00697 | hsa2555 | 16.284 | Novel |
| D00704 | hsa2555 | 16.284 | Novel |
| D01071 | hsa2555 | 16.284 | Novel |
| D02347 | hsa6263 | 16.167 | Novel |
| D00823 | hsa3781 | 15.193 | Exist |
| D00326 | hsa3781 | 15.151 | Exist |
| D00283 | hsa3359 | 15.06 | Exist |
| D00392 | hsa8912 | 15 | Novel |
| D00536 | hsa2554 | 15 | Novel |
| D00451 | hsa285242 | 15 | Exist |
| D00513 | hsa285242 | 15 | Exist |
| D00495 | hsa8913 | 14.272 | Novel |
| D03742 | hsa1136 | 14.159 | Novel |
| D00761 | hsa1136 | 14.151 | Novel |
| D00779 | hsa1136 | 14.098 | Novel |
| D00759 | hsa1136 | 14.054 | Novel |
| D00760 | hsa1136 | 14.054 | Novel |
| D00765 | hsa1136 | 14.054 | Novel |
| D03878 | hsa2903 | 12.044 | Exist |
| D00136 | hsa116443 | 12 | Novel |
| D03274 | hsa3780 | 9.216 | Novel |
| D04034 | hsa3780 | 9.216 | Novel |
| D00809 | hsa3780 | 9.213 | Novel |
| D00647 | hsa9424 | 9 | Novel |
| D04370 | hsa285242 | 8.984 | Novel |
| D00456 | hsa285242 | 8.619 | Novel |
| D00563 | hsa285242 | 8.06 | Novel |
| D00799 | hsa3782 | 8.021 | Novel |
| D00791 | hsa3781 | 7.806 | Novel |
| D00816 | hsa3781 | 7.723 | Novel |
| D02163 | hsa3781 | 7.615 | Novel |
| D00812 | hsa3781 | 7.533 | Novel |
| D01448 | hsa3782 | 7.496 | Novel |
| D00480 | hsa3781 | 7.303 | Novel |
| D01242 | hsa3781 | 7.129 | Novel |
| D02234 | hsa3782 | 7.125 | Novel |
| D02262 | hsa3741 | 7.074 | Novel |
| D00351 | hsa3743 | 7.067 | Novel |
| D00336 | hsa3783 | 7 | Novel |
| D02261 | hsa3737 | 6.18 | Novel |
| D00364 | hsa6331 | 6.142 | Novel |
| D00228 | hsa6531 | 5.251 | Novel |
| D00394 | hsa6531 | 5.178 | Novel |
| D01575 | hsa3781 | 5.133 | Novel |
| D00636 | hsa3741 | 5.053 | Novel |
| D03450 | hsa6261 | 5 | Novel |
| D02546 | hsa2899 | 4.677 | Exist |
| D00798 | hsa3782 | 4.318 | Novel |
| D04999 | hsa6530 | 4.251 | Exist |
| D00367 | hsa6530 | 4.25 | Exist |
| D01256 | hsa2890 | 4.213 | Novel |
| D00373 | hsa3782 | 4.197 | Novel |
| D00607 | hsa6531 | 4.181 | Novel |
| D02237 | hsa6531 | 4.181 | Novel |
| D02566 | hsa6531 | 4.179 | Novel |
| D01603 | hsa6531 | 4.177 | Novel |
| D05458 | hsa6531 | 4.177 | Exist |
| D00345 | hsa3778 | 4 | Novel |
| D00574 | hsa1135 | 4 | Novel |
| D00960 | hsa1135 | 4 | Novel |
| D00963 | hsa1135 | 4 | Novel |
| D00964 | hsa1135 | 4 | Novel |
| D00335 | hsa6833 | 3.325 | Novel |
| D02360 | hsa6530 | 3.194 | Novel |
| D00219 | hsa6833 | 3.19 | Novel |
| D00380 | hsa3758 | 3.147 | Exist |
| D00379 | hsa6833 | 3.13 | Novel |
| D00593 | hsa6833 | 3.077 | Novel |
| D00274 | hsa90134 | 3.066 | Novel |
| D02092 | hsa90134 | 3.066 | Novel |
| D00234 | hsa90134 | 3.056 | Novel |
| D00521 | hsa90134 | 3.056 | Novel |
| D02485 | hsa90134 | 3.055 | Novel |
| D00340 | hsa3783 | 3 | Novel |
| D00418 | hsa6833 | 3 | Novel |
| D00519 | hsa3783 | 3 | Novel |
| D00594 | hsa6833 | 3 | Novel |
| D00650 | hsa3783 | 3 | Novel |
| D00651 | hsa3783 | 3 | Novel |
| D00654 | hsa3783 | 3 | Novel |
| D00656 | hsa3783 | 3 | Novel |
| D00658 | hsa3783 | 3 | Novel |
| D00771 | hsa3783 | 3 | Novel |
| D00831 | hsa3753 | 3 | Novel |
| D01111 | hsa6833 | 3 | Novel |
| D01828 | hsa3758 | 3 | Novel |
| D02362 | hsa6530 | 3 | Novel |
| D03037 | hsa3753 | 3 | Novel |
| D01799 | hsa3758 | 2.637 | Novel |
| D01599 | hsa3758 | 2.586 | Novel |
| D01118 | hsa6328 | 2.073 | Novel |
| D00631 | hsa10008 | 2 | Novel |
| D01854 | hsa3758 | 2 | Novel |
| D00035 | hsa11254 | 1 | Exist |
| D04790 | hsa1181 | 1 | Exist |

Table S6. ‘Top 1’ predictions for the Enzyme Dataset. ‘Exist’ refers to predictions of links that represent known DTIs in the network. Novel refers to new predictions that are not known DTIs in the network.

| **chemID (KEGG)** | **protID (KEGG)** | **Score** | **Type** |
| --- | --- | --- | --- |
| D00225 | hsa1576 | 598.357 | Exist |
| D00394 | hsa1576 | 596.907 | Exist |
| D00380 | hsa1576 | 596.902 | Exist |
| D01071 | hsa1576 | 596.902 | Exist |
| D00542 | hsa1571 | 593.788 | Novel |
| D00574 | hsa1576 | 587.902 | Exist |
| D00528 | hsa1549 | 587.629 | Novel |
| D00139 | hsa1576 | 581.902 | Exist |
| D00437 | hsa1559 | 581.638 | Novel |
| D03670 | hsa1544 | 567.249 | Exist |
| D00410 | hsa1576 | 565.902 | Exist |
| D03781 | hsa1576 | 414.902 | Exist |
| D03784 | hsa1576 | 414.902 | Exist |
| D00964 | hsa1576 | 402.962 | Exist |
| D03778 | hsa1576 | 402.962 | Exist |
| D00960 | hsa1576 | 402.902 | Exist |
| D01425 | hsa1576 | 402.902 | Exist |
| D02451 | hsa1576 | 402.902 | Exist |
| D00417 | hsa27115 | 392.393 | Exist |
| D01977 | hsa3643 | 366.504 | Exist |
| D03350 | hsa3643 | 366.431 | Exist |
| D03252 | hsa3643 | 366.345 | Exist |
| D04023 | hsa3643 | 366.223 | Exist |
| D01441 | hsa3643 | 366.122 | Exist |
| D03218 | hsa3643 | 366.122 | Exist |
| D04024 | hsa3643 | 366.122 | Exist |
| D04025 | hsa3643 | 366.122 | Exist |
| D00501 | hsa5144 | 359.743 | Exist |
| D00371 | hsa27115 | 337.017 | Exist |
| D02731 | hsa27115 | 336.8 | Exist |
| D03260 | hsa27115 | 336.792 | Exist |
| D01712 | hsa27115 | 336.673 | Exist |
| D02017 | hsa27115 | 336.566 | Exist |
| D00231 | hsa27115 | 336.265 | Exist |
| D02229 | hsa27115 | 336.236 | Exist |
| D02042 | hsa27115 | 336.234 | Exist |
| D00227 | hsa27115 | 336.1 | Exist |
| D01133 | hsa27115 | 336.1 | Exist |
| D01198 | hsa27115 | 336.1 | Exist |
| D01690 | hsa27115 | 336.1 | Exist |
| D01704 | hsa27115 | 336.1 | Exist |
| D02008 | hsa27115 | 336.1 | Exist |
| D02655 | hsa27115 | 336.1 | Exist |
| D03217 | hsa27115 | 336.1 | Exist |
| D00691 | hsa5152 | 285.538 | Novel |
| D00340 | hsa760 | 126.719 | Exist |
| D00519 | hsa760 | 126.122 | Exist |
| D00653 | hsa760 | 125.97 | Exist |
| D00218 | hsa760 | 125.955 | Exist |
| D03845 | hsa760 | 125.872 | Exist |
| D01196 | hsa760 | 125.848 | Exist |
| D00652 | hsa760 | 125.763 | Exist |
| D00655 | hsa760 | 125.654 | Exist |
| D00518 | hsa760 | 125.559 | Exist |
| D00538 | hsa760 | 125.367 | Exist |
| D00097 | hsa5743 | 94.961 | Novel |
| D00132 | hsa5742 | 94.938 | Exist |
| D00510 | hsa5742 | 92.136 | Exist |
| D00118 | hsa5742 | 91.861 | Exist |
| D00141 | hsa5742 | 91.336 | Exist |
| D00217 | hsa1576 | 86 | Novel |
| D00126 | hsa5742 | 84.374 | Exist |
| D00283 | hsa1559 | 74.879 | Novel |
| D00771 | hsa1565 | 74 | Novel |
| D00377 | hsa5742 | 66.673 | Exist |
| D01364 | hsa5742 | 65.133 | Exist |
| D02350 | hsa5742 | 62.862 | Exist |
| D00330 | hsa5742 | 62.821 | Exist |
| D00903 | hsa5742 | 62.31 | Exist |
| D00904 | hsa5742 | 62.31 | Exist |
| D02341 | hsa5742 | 62.255 | Exist |
| D00452 | hsa5743 | 62.116 | Exist |
| D01325 | hsa5742 | 62.096 | Exist |
| D00169 | hsa5742 | 61.923 | Exist |
| D03714 | hsa5742 | 61.885 | Exist |
| D00151 | hsa5742 | 61.864 | Exist |
| D00127 | hsa5742 | 61.826 | Exist |
| D01767 | hsa5742 | 61.818 | Exist |
| D01183 | hsa5742 | 61.701 | Exist |
| D00567 | hsa5742 | 61.593 | Exist |
| D02290 | hsa5742 | 61.577 | Exist |
| D01866 | hsa5742 | 61.543 | Exist |
| D00120 | hsa5742 | 61.317 | Exist |
| D03716 | hsa5742 | 61.289 | Exist |
| D01547 | hsa5742 | 61.275 | Exist |
| D01578 | hsa5742 | 61.219 | Exist |
| D03717 | hsa5742 | 61.207 | Exist |
| D02355 | hsa5742 | 61.203 | Exist |
| D02709 | hsa5742 | 61.163 | Exist |
| D00130 | hsa5742 | 61.13 | Exist |
| D00970 | hsa5742 | 61.076 | Exist |
| D00969 | hsa5742 | 61.037 | Exist |
| D01582 | hsa5742 | 60.94 | Exist |
| D02110 | hsa5742 | 60.916 | Exist |
| D00158 | hsa5742 | 60.79 | Exist |
| D01397 | hsa5742 | 60.688 | Exist |
| D00566 | hsa5742 | 60.683 | Exist |
| D01718 | hsa5743 | 60.64 | Exist |
| D03689 | hsa5742 | 60.492 | Exist |
| D03710 | hsa5742 | 60.465 | Exist |
| D00109 | hsa5742 | 60.379 | Exist |
| D00425 | hsa5742 | 60.376 | Exist |
| D00463 | hsa5742 | 60.353 | Exist |
| D01811 | hsa5742 | 60.334 | Exist |
| D01475 | hsa5742 | 60.327 | Exist |
| D01513 | hsa5742 | 60.311 | Exist |
| D00449 | hsa5742 | 60.308 | Novel |
| D01565 | hsa5742 | 60.302 | Exist |
| D00810 | hsa5742 | 60.254 | Exist |
| D00568 | hsa5742 | 60.233 | Exist |
| D01122 | hsa5742 | 60.192 | Exist |
| D00813 | hsa5742 | 60.183 | Exist |
| D03712 | hsa5742 | 60.177 | Exist |
| D00315 | hsa5742 | 60.133 | Exist |
| D00827 | hsa5742 | 60.133 | Exist |
| D00968 | hsa5742 | 60.133 | Exist |
| D01709 | hsa5742 | 60.133 | Exist |
| D01765 | hsa5742 | 60.133 | Exist |
| D01974 | hsa5742 | 60.133 | Exist |
| D05353 | hsa1565 | 60 | Novel |
| D00563 | hsa1557 | 59.06 | Novel |
| D00107 | hsa5501 | 50.429 | Exist |
| D00184 | hsa5501 | 50.429 | Exist |
| D00454 | hsa1576 | 44.178 | Novel |
| D00293 | hsa1565 | 43.193 | Novel |
| D00503 | hsa1544 | 42.113 | Novel |
| D00252 | hsa1565 | 42.004 | Novel |
| D00234 | hsa1544 | 42 | Novel |
| D00274 | hsa1544 | 42 | Novel |
| D00521 | hsa1544 | 42 | Novel |
| D02356 | hsa1565 | 42 | Novel |
| D00043 | hsa2155 | 40 | Exist |
| D00160 | hsa2155 | 40 | Exist |
| D00569 | hsa1565 | 39.219 | Novel |
| D00416 | hsa1576 | 39.084 | Novel |
| D00882 | hsa1576 | 39.006 | Novel |
| D00322 | hsa1576 | 39 | Novel |
| D00512 | hsa1576 | 39 | Novel |
| D00658 | hsa760 | 38.467 | Exist |
| D00654 | hsa760 | 38.199 | Exist |
| D00448 | hsa5742 | 38.058 | Novel |
| D00656 | hsa760 | 38.046 | Exist |
| D03365 | hsa1548 | 37.31 | Novel |
| D01332 | hsa10846 | 37 | Novel |
| D00650 | hsa760 | 35.659 | Exist |
| D01256 | hsa760 | 35.61 | Exist |
| D00651 | hsa760 | 35.315 | Exist |
| D00537 | hsa759 | 35.103 | Novel |
| D00294 | hsa760 | 35.103 | Exist |
| D00703 | hsa762 | 35 | Novel |
| D01840 | hsa5599 | 26.08 | Exist |
| D03115 | hsa5599 | 26.08 | Exist |
| D03736 | hsa5599 | 26.08 | Exist |
| D00577 | hsa1733 | 26 | Exist |
| D00136 | hsa1565 | 25.131 | Novel |
| D00387 | hsa1565 | 24.671 | Novel |
| D00550 | hsa1565 | 24.591 | Novel |
| D02671 | hsa1576 | 24.179 | Novel |
| D00300 | hsa1576 | 24.12 | Novel |
| D00364 | hsa1565 | 24.12 | Novel |
| D01164 | hsa1576 | 24.063 | Novel |
| D01973 | hsa1565 | 24.044 | Novel |
| D00434 | hsa1565 | 24 | Novel |
| D02342 | hsa1576 | 24 | Novel |
| D01275 | hsa7153 | 23.836 | Exist |
| D02214 | hsa7153 | 23.836 | Exist |
| D03899 | hsa7153 | 23.516 | Exist |
| D00533 | hsa1559 | 22.981 | Novel |
| D00593 | hsa1557 | 22.88 | Novel |
| D00536 | hsa1559 | 22.879 | Novel |
| D01264 | hsa7153 | 22.741 | Exist |
| D00544 | hsa1544 | 21.169 | Novel |
| D00543 | hsa1544 | 21.072 | Novel |
| D00547 | hsa1544 | 21.001 | Novel |
| D01747 | hsa7153 | 20.411 | Exist |
| D02756 | hsa7153 | 20.222 | Exist |
| D01911 | hsa7153 | 20.219 | Exist |
| D00596 | hsa1576 | 20 | Novel |
| D05341 | hsa1576 | 20 | Novel |
| D00562 | hsa1733 | 20 | Exist |
| D00785 | hsa4129 | 19.512 | Exist |
| D03731 | hsa4129 | 19.467 | Exist |
| D01885 | hsa7153 | 19.213 | Exist |
| D00037 | hsa10846 | 19 | Novel |
| D00131 | hsa1544 | 19 | Novel |
| D02564 | hsa4129 | 18.942 | Exist |
| D05458 | hsa4128 | 18.476 | Novel |
| D02561 | hsa4128 | 18.368 | Exist |
| D02563 | hsa4128 | 18.328 | Exist |
| D02581 | hsa4128 | 18.328 | Exist |
| D01097 | hsa4128 | 18.325 | Exist |
| D00947 | hsa4129 | 18.314 | Novel |
| D00505 | hsa4129 | 18.314 | Exist |
| D02562 | hsa4128 | 18.314 | Exist |
| D00005 | hsa4128 | 18.269 | Novel |
| D00270 | hsa4128 | 18.269 | Exist |
| D00826 | hsa4128 | 18.269 | Exist |
| D01888 | hsa4128 | 18.269 | Exist |
| D02559 | hsa4128 | 18.269 | Exist |
| D02560 | hsa4128 | 18.269 | Exist |
| D02579 | hsa4128 | 18.269 | Exist |
| D02580 | hsa4128 | 18.269 | Exist |
| D03733 | hsa4128 | 18.269 | Exist |
| D04092 | hsa4128 | 18.269 | Exist |
| D00401 | hsa8288 | 18.2 | Novel |
| D02441 | hsa760 | 18.147 | Novel |
| D00709 | hsa760 | 18.103 | Novel |
| D00153 | hsa1544 | 18 | Novel |
| D00963 | hsa1544 | 18 | Novel |
| D04031 | hsa116447 | 16.317 | Exist |
| D02168 | hsa116447 | 16.265 | Exist |
| D01061 | hsa116447 | 16.174 | Exist |
| D04966 | hsa3480 | 16.137 | Novel |
| D01432 | hsa116447 | 16.121 | Exist |
| D00125 | hsa7153 | 15.732 | Exist |
| D02698 | hsa7153 | 15.732 | Exist |
| D00183 | hsa7153 | 15.166 | Exist |
| D00186 | hsa7153 | 15.166 | Exist |
| D02166 | hsa7153 | 15.166 | Exist |
| D02321 | hsa7153 | 15.166 | Exist |
| D02333 | hsa7153 | 15.166 | Exist |
| D00188 | hsa1594 | 11.74 | Novel |
| D00129 | hsa1593 | 11.4 | Novel |
| D00187 | hsa1593 | 11.339 | Novel |
| D02556 | hsa1557 | 11.12 | Novel |
| D01223 | hsa43 | 10.059 | Exist |
| D03822 | hsa43 | 9.526 | Exist |
| D00196 | hsa43 | 9.328 | Exist |
| D00995 | hsa590 | 9.227 | Novel |
| D02418 | hsa590 | 9.224 | Novel |
| D01228 | hsa590 | 9.197 | Novel |
| D00667 | hsa43 | 9.196 | Exist |
| D02558 | hsa590 | 9.195 | Novel |
| D03823 | hsa590 | 9.166 | Novel |
| D00998 | hsa590 | 9.165 | Novel |
| D00487 | hsa590 | 9.122 | Novel |
| D03751 | hsa590 | 9.107 | Novel |
| D00670 | hsa43 | 9.107 | Exist |
| D02173 | hsa590 | 9.069 | Novel |
| D04292 | hsa590 | 9.069 | Novel |
| D00994 | hsa590 | 9.061 | Novel |
| D00733 | hsa43 | 9.059 | Novel |
| D01001 | hsa590 | 9.059 | Novel |
| D01118 | hsa43 | 9.059 | Novel |
| D02068 | hsa590 | 9.059 | Novel |
| D03826 | hsa590 | 9.059 | Novel |
| D00805 | hsa43 | 9.059 | Exist |
| D02193 | hsa43 | 9.059 | Exist |
| D02729 | hsa43 | 9.059 | Exist |
| D01276 | hsa10720 | 8.77 | Exist |
| D04028 | hsa5406 | 8.389 | Exist |
| D00414 | hsa239 | 8 | Novel |
| D00726 | hsa10461 | 8 | Novel |
| D03882 | hsa239 | 8 | Novel |
| D00216 | hsa2548 | 8 | Exist |
| D00625 | hsa2548 | 8 | Exist |
| D00545 | hsa27032 | 7.417 | Exist |
| D00546 | hsa27032 | 7.417 | Exist |
| D00900 | hsa10825 | 7 | Exist |
| D00902 | hsa10825 | 7 | Exist |
| D01665 | hsa2548 | 7 | Exist |
| D03433 | hsa2548 | 7 | Exist |
| D03829 | hsa10825 | 7 | Exist |
| D03756 | hsa1636 | 6.991 | Exist |
| D00383 | hsa1636 | 6.971 | Exist |
| D00421 | hsa1636 | 6.598 | Exist |
| D00039 | hsa5095 | 6.551 | Exist |
| D00459 | hsa1636 | 6.473 | Exist |
| D03752 | hsa1636 | 6.439 | Exist |
| D00065 | hsa5095 | 6.438 | Exist |
| D02335 | hsa10279 | 6 | Novel |
| D03765 | hsa1636 | 5.289 | Exist |
| D01667 | hsa1636 | 4.938 | Exist |
| D00041 | hsa586 | 4.55 | Novel |
| D01549 | hsa1636 | 4.375 | Exist |
| D00002 | hsa125 | 4.21 | Exist |
| D00423 | hsa5169 | 4.058 | Novel |
| D01136 | hsa10279 | 4 | Novel |
| D00029 | hsa10295 | 4 | Exist |
| D01966 | hsa6646 | 4 | Exist |
| D03012 | hsa6646 | 4 | Exist |
| D03734 | hsa6646 | 4 | Exist |
| D03735 | hsa6646 | 4 | Exist |
| D00623 | hsa1636 | 3.857 | Exist |
| D03773 | hsa1636 | 3.621 | Exist |
| D03753 | hsa1636 | 3.286 | Exist |
| D00038 | hsa8288 | 3.261 | Novel |
| D00620 | hsa1636 | 3.23 | Exist |
| D00621 | hsa1636 | 3.157 | Exist |
| D03769 | hsa1636 | 3.067 | Exist |
| D00298 | hsa759 | 3 | Novel |
| D00332 | hsa10295 | 3 | Novel |
| D01240 | hsa759 | 3 | Novel |
| D01844 | hsa2155 | 3 | Novel |
| D04029 | hsa2155 | 3 | Novel |
| D03775 | hsa1636 | 2.882 | Exist |
| D01119 | hsa1636 | 2.55 | Exist |
| D00222 | hsa30 | 2.532 | Exist |
| D00094 | hsa217 | 2.411 | Novel |
| D00624 | hsa1636 | 2.393 | Exist |
| D00333 | hsa28 | 2.345 | Novel |
| D00362 | hsa1636 | 2.264 | Exist |
| D02323 | hsa57016 | 2.255 | Novel |
| D01346 | hsa5407 | 2.211 | Novel |
| D01069 | hsa1636 | 2.208 | Exist |
| D02769 | hsa5834 | 2.191 | Exist |
| D00398 | hsa28 | 2.101 | Novel |
| D05407 | hsa28 | 2.085 | Novel |
| D01825 | hsa5321 | 2.079 | Exist |
| D00219 | hsa1576 | 2.076 | Novel |
| D03760 | hsa1636 | 2.005 | Exist |
| D00203 | hsa30 | 2 | Novel |
| D00208 | hsa4835 | 2 | Novel |
| D00342 | hsa28 | 2 | Novel |
| D00391 | hsa28 | 2 | Novel |
| D00884 | hsa30 | 2 | Novel |
| D00885 | hsa30 | 2 | Novel |
| D01688 | hsa10327 | 2 | Novel |
| D01715 | hsa10327 | 2 | Novel |
| D01842 | hsa10327 | 2 | Novel |
| D02328 | hsa10327 | 2 | Novel |
| D02835 | hsa10327 | 2 | Novel |
| D03803 | hsa10327 | 2 | Novel |
| D03805 | hsa10327 | 2 | Novel |
| D03806 | hsa10327 | 2 | Novel |
| D03807 | hsa10327 | 2 | Novel |
| D00317 | hsa28 | 2 | Exist |
| D01027 | hsa4143 | 2 | Exist |
| D03798 | hsa1610 | 2 | Exist |
| D04983 | hsa326625 | 2 | Exist |
| D03077 | hsa1636 | 1.946 | Exist |
| D00325 | hsa5321 | 1.913 | Exist |
| D00328 | hsa5321 | 1.836 | Exist |
| D00142 | hsa1719 | 1.7 | Exist |
| D02115 | hsa1719 | 1.7 | Exist |
| D00359 | hsa3156 | 1.692 | Exist |
| D02880 | hsa1017 | 1.595 | Exist |
| D02258 | hsa3156 | 1.555 | Exist |
| D01367 | hsa5321 | 1.498 | Exist |
| D00035 | hsa29920 | 1.497 | Exist |
| D04197 | hsa30 | 1.478 | Novel |
| D00045 | hsa132 | 1.445 | Novel |
| D03772 | hsa1636 | 1.435 | Exist |
| D03776 | hsa1636 | 1.405 | Exist |
| D00887 | hsa3156 | 1.402 | Exist |
| D03758 | hsa1636 | 1.389 | Exist |
| D01918 | hsa1991 | 1.376 | Exist |
| D03788 | hsa1991 | 1.376 | Exist |
| D00752 | hsa3614 | 1.366 | Exist |
| D03440 | hsa1636 | 1.319 | Exist |
| D00622 | hsa1636 | 1.296 | Exist |
| D02289 | hsa5321 | 1.288 | Exist |
| D02566 | hsa6241 | 1.287 | Novel |
| D03828 | hsa7298 | 1.277 | Exist |
| D00007 | hsa32 | 1.248 | Novel |
| D00027 | hsa32 | 1.248 | Novel |
| D00494 | hsa590 | 1.236 | Novel |
| D00145 | hsa1719 | 1.218 | Exist |
| D06238 | hsa1719 | 1.215 | Exist |
| D01211 | hsa7298 | 1.21 | Exist |
| D00516 | hsa4881 | 1.207 | Exist |
| D00630 | hsa4881 | 1.207 | Exist |
| D00584 | hsa1806 | 1.199 | Exist |
| D00889 | hsa3156 | 1.16 | Exist |
| D00892 | hsa3156 | 1.16 | Exist |
| D00285 | hsa1719 | 1.136 | Exist |
| D02368 | hsa7298 | 1.128 | Exist |
| D00021 | hsa7054 | 1.121 | Exist |
| D00762 | hsa10056 | 1.121 | Exist |
| D00579 | hsa7015 | 1.12 | Exist |
| D02267 | hsa7015 | 1.12 | Exist |
| D00054 | hsa4860 | 1.117 | Exist |
| D01370 | hsa4860 | 1.117 | Exist |
| D03745 | hsa5972 | 1.101 | Exist |
| D03738 | hsa5972 | 1.098 | Exist |
| D00279 | hsa43 | 1.091 | Novel |
| D01064 | hsa7298 | 1.08 | Exist |
| D00893 | hsa3156 | 1.068 | Exist |
| D01900 | hsa1636 | 1.065 | Exist |
| D01862 | hsa3156 | 1.056 | Exist |
| D01915 | hsa3156 | 1.056 | Exist |
| D03743 | hsa5972 | 1.047 | Exist |
| D02308 | hsa2746 | 1.044 | Novel |
| D00455 | hsa495 | 1.006 | Exist |
| D01907 | hsa7298 | 1.002 | Novel |
| D00535 | hsa586 | 1.001 | Novel |
| D00560 | hsa4128 | 1.001 | Novel |
| D00475 | hsa6898 | 1 | Novel |
| D00014 | hsa10 | 1 | Exist |
| D00018 | hsa5033 | 1 | Exist |
| D00032 | hsa3034 | 1 | Exist |
| D00036 | hsa683 | 1 | Exist |
| D00049 | hsa23475 | 1 | Exist |
| D00050 | hsa55312 | 1 | Exist |
| D00052 | hsa1363 | 1 | Exist |
| D00055 | hsa1723 | 1 | Exist |
| D00070 | hsa2356 | 1 | Exist |
| D00086 | hsa51601 | 1 | Exist |
| D00103 | hsa51205 | 1 | Exist |
| D00148 | hsa10901 | 1 | Exist |
| D00155 | hsa100 | 1 | Exist |
| D00168 | hsa5423 | 1 | Exist |
| D00185 | hsa1557 | 1 | Exist |
| D00198 | hsa1636 | 1 | Exist |
| D00224 | hsa1610 | 1 | Exist |
| D00251 | hsa1636 | 1 | Exist |
| D00324 | hsa1056 | 1 | Exist |
| D00369 | hsa189 | 1 | Exist |
| D00418 | hsa10549 | 1 | Exist |
| D00451 | hsa1544 | 1 | Exist |
| D00488 | hsa1719 | 1 | Exist |
| D00496 | hsa834 | 1 | Exist |
| D00513 | hsa1544 | 1 | Exist |
| D00515 | hsa4881 | 1 | Exist |
| D00530 | hsa1557 | 1 | Exist |
| D00549 | hsa1557 | 1 | Exist |
| D00749 | hsa1723 | 1 | Exist |
| D00753 | hsa2280 | 1 | Exist |
| D00781 | hsa1312 | 1 | Exist |
| D00786 | hsa1312 | 1 | Exist |
| D00829 | hsa4953 | 1 | Exist |
| D00965 | hsa1728 | 1 | Exist |
| D01180 | hsa3283 | 1 | Exist |
| D01828 | hsa2135 | 1 | Exist |
| D01968 | hsa2224 | 1 | Exist |
| D01981 | hsa10901 | 1 | Exist |
| D01984 | hsa495 | 1 | Exist |
| D02176 | hsa1374 | 1 | Exist |
| D02194 | hsa1800 | 1 | Exist |
| D02304 | hsa3735 | 1 | Exist |
| D02315 | hsa2638 | 1 | Exist |
| D02375 | hsa6713 | 1 | Exist |
| D02487 | hsa1719 | 1 | Exist |
| D03034 | hsa6716 | 1 | Exist |
| D03208 | hsa5972 | 1 | Exist |
| D03601 | hsa1576 | 1 | Exist |
| D03643 | hsa1576 | 1 | Exist |
| D03720 | hsa2339 | 1 | Exist |
| D03722 | hsa10901 | 1 | Exist |
| D03728 | hsa10901 | 1 | Exist |
| D03741 | hsa5972 | 1 | Exist |
| D03763 | hsa1636 | 1 | Exist |
| D03767 | hsa1636 | 1 | Exist |
| D03787 | hsa1571 | 1 | Exist |
| D03816 | hsa1576 | 1 | Exist |

Table S7. ‘Top 1’ predictions for the DrugBank_approved Dataset. ‘Exist’ refers to predictions of links that represent known DTIs in the network. Novel refers to new predictions that are not known DTIs in the network.

| **chemID (DrugBank)** | **protID (UniProt)** | **Score** | **Type** |
| --- | --- | --- | --- |
| DB00312 | P14867 | 797.059 | Exist |
| DB00829 | P14867 | 776.977 | Exist |
| DB00231 | P14867 | 775.296 | Exist |
| DB00897 | P14867 | 773.604 | Exist |
| DB00306 | P14867 | 771.79 | Exist |
| DB00237 | P14867 | 771.48 | Exist |
| DB00241 | P14867 | 770.497 | Exist |
| DB00794 | P14867 | 768.857 | Exist |
| DB00842 | P47869 | 766.198 | Exist |
| DB01558 | P47869 | 766.189 | Exist |
| DB01595 | P47869 | 765.491 | Exist |
| DB00801 | P47869 | 764.503 | Exist |
| DB00683 | P47869 | 764.207 | Exist |
| DB01588 | P47869 | 764.205 | Exist |
| DB01215 | P47869 | 763.653 | Exist |
| DB00690 | P47869 | 762.676 | Exist |
| DB01589 | P47869 | 761.502 | Exist |
| DB00334 | P47869 | 756.86 | Exist |
| DB00543 | P47869 | 755.974 | Exist |
| DB01049 | P47869 | 748.783 | Exist |
| DB01159 | P14867 | 748.193 | Exist |
| DB01189 | P14867 | 743.528 | Exist |
| DB01028 | P14867 | 742.095 | Exist |
| DB01236 | P14867 | 741.985 | Exist |
| DB00753 | P14867 | 740.528 | Exist |
| DB00228 | P14867 | 740.474 | Exist |
| DB00659 | P14867 | 739.761 | Exist |
| DB00186 | P14867 | 729.871 | Exist |
| DB01068 | P14867 | 728.436 | Exist |
| DB00628 | P14867 | 727.554 | Exist |
| DB00402 | P14867 | 725.067 | Exist |
| DB00404 | P47869 | 719.7 | Exist |
| DB00292 | P47869 | 717.783 | Exist |
| DB00349 | P14867 | 717.67 | Exist |
| DB00475 | P47869 | 716.309 | Exist |
| DB00273 | P47869 | 715.783 | Exist |
| DB01437 | P14867 | 715.639 | Exist |
| DB01107 | P47869 | 715.185 | Exist |
| DB00189 | P47869 | 714.783 | Exist |
| DB00371 | P47869 | 714.783 | Exist |
| DB00818 | P47869 | 714.783 | Exist |
| DB01205 | P47869 | 714.783 | Exist |
| DB00408 | P28223 | 686.471 | Exist |
| DB01224 | P28223 | 646.491 | Exist |
| DB01567 | P14867 | 628.85 | Exist |
| DB00246 | P28223 | 626.662 | Exist |
| DB01238 | P28223 | 626.517 | Exist |
| DB01594 | P14867 | 623.933 | Exist |
| DB00546 | P14867 | 623.436 | Exist |
| DB01559 | P14867 | 622.834 | Exist |
| DB00363 | P28223 | 617.066 | Exist |
| DB00540 | P35348 | 580.524 | Exist |
| DB00477 | P35348 | 576.687 | Exist |
| DB00726 | P35348 | 566.893 | Exist |
| DB01142 | P35348 | 566.609 | Exist |
| DB01151 | P35348 | 557.163 | Exist |
| DB06148 | P35348 | 548.941 | Exist |
| DB01403 | P35348 | 535.11 | Exist |
| DB00321 | P35348 | 534.309 | Exist |
| DB00248 | P35348 | 527.056 | Exist |
| DB00370 | P35348 | 506.321 | Exist |
| DB00458 | P28223 | 503.212 | Exist |
| DB01200 | P14416 | 498.42 | Exist |
| DB00934 | P35348 | 491.157 | Exist |
| DB01186 | P14416 | 479.137 | Exist |
| DB06216 | P28223 | 472.235 | Exist |
| DB00734 | P14416 | 426.749 | Exist |
| DB01267 | P14416 | 426.703 | Exist |
| DB00696 | P35348 | 413.359 | Exist |
| DB00714 | P14416 | 395.147 | Exist |
| DB00589 | P14416 | 393.082 | Exist |
| DB00268 | P14416 | 393 | Exist |
| DB00413 | P14416 | 393 | Exist |
| DB00420 | P28223 | 386.318 | Exist |
| DB00777 | P28223 | 381.411 | Exist |
| DB00182 | P35348 | 358.18 | Exist |
| DB00418 | P14867 | 347.025 | Exist |
| DB01221 | P28223 | 331 | Exist |
| DB01353 | P14867 | 321.399 | Exist |
| DB01352 | P14867 | 320.931 | Exist |
| DB01351 | P14867 | 320.648 | Exist |
| DB00599 | P14867 | 319.808 | Exist |
| DB00849 | P14867 | 319.015 | Exist |
| DB01239 | P28223 | 318.885 | Exist |
| DB01354 | P47869 | 318.077 | Exist |
| DB01392 | P35348 | 318 | Novel |
| DB01355 | P47869 | 317.965 | Exist |
| DB04946 | P08913 | 317.445 | Novel |
| DB01069 | P11229 | 285.846 | Exist |
| DB01544 | P14867 | 284.337 | Novel |
| DB00368 | P35348 | 276.319 | Exist |
| DB00668 | P35348 | 272.306 | Exist |
| DB06262 | P35348 | 270.793 | Exist |
| DB01136 | P35348 | 270.08 | Exist |
| DB01365 | P35348 | 268.675 | Exist |
| DB00800 | P35348 | 268 | Exist |
| DB01149 | P14416 | 260.577 | Novel |
| DB00988 | P28223 | 260 | Novel |
| DB00568 | P11229 | 259.424 | Exist |
| DB04855 | P35348 | 254 | Exist |
| DB01587 | P47869 | 251.328 | Novel |
| DB00925 | P35348 | 245.365 | Exist |
| DB06144 | P08913 | 236.107 | Novel |
| DB00656 | P14416 | 235.369 | Novel |
| DB00622 | P11229 | 235 | Exist |
| DB00715 | P11229 | 230.006 | Exist |
| DB05271 | P28223 | 227.057 | Novel |
| DB01624 | P35368 | 226.75 | Novel |
| DB00575 | P35348 | 222.546 | Exist |
| DB06694 | P35348 | 222.526 | Exist |
| DB00935 | P35348 | 222.484 | Exist |
| DB00454 | P11229 | 216.549 | Exist |
| DB01614 | P08913 | 216.179 | Novel |
| DB01622 | P08913 | 215.308 | Novel |
| DB01198 | Q16445 | 212.393 | Novel |
| DB08815 | P35348 | 210 | Novel |
| DB00835 | P11229 | 209.153 | Exist |
| DB00457 | P35348 | 201.65 | Exist |
| DB00247 | P14416 | 196.369 | Novel |
| DB00797 | P35368 | 191.063 | Novel |
| DB00805 | P28223 | 191.006 | Exist |
| DB00679 | P08913 | 190.249 | Novel |
| DB00434 | P14416 | 184.543 | Novel |
| DB01577 | P35348 | 184.518 | Novel |
| DB00572 | P11229 | 182.609 | Exist |
| DB00747 | P08172 | 181.318 | Exist |
| DB00725 | P11229 | 181.316 | Exist |
| DB00376 | P11229 | 180.891 | Exist |
| DB00340 | P11229 | 180.484 | Exist |
| DB01036 | P11229 | 179.796 | Exist |
| DB06702 | P11229 | 179.351 | Exist |
| DB00496 | P08172 | 179.197 | Exist |
| DB08897 | P11229 | 179.11 | Exist |
| DB01591 | P08172 | 179.07 | Exist |
| DB00217 | P35348 | 173.627 | Novel |
| DB00751 | P14416 | 173.47 | Novel |
| DB00852 | P35368 | 168.725 | Novel |
| DB00425 | P31644 | 167.636 | Novel |
| DB00875 | P35368 | 167.016 | Novel |
| DB00508 | P28223 | 163.389 | Novel |
| DB01235 | P28223 | 160 | Novel |
| DB00397 | P35368 | 156.081 | Novel |
| DB00598 | P08913 | 156.009 | Novel |
| DB00424 | P11229 | 148.609 | Exist |
| DB00387 | P11229 | 146.73 | Exist |
| DB00809 | P08172 | 145.083 | Exist |
| DB01242 | P14416 | 141.487 | Novel |
| DB01608 | P35348 | 141.288 | Novel |
| DB01621 | P28335 | 139.59 | Novel |
| DB01175 | P35368 | 138.764 | Novel |
| DB00216 | P28223 | 138.165 | Novel |
| DB00907 | P20309 | 136.543 | Novel |
| DB01174 | P47869 | 136.109 | Novel |
| DB00409 | P21728 | 136 | Novel |
| DB00964 | P35368 | 132.446 | Novel |
| DB01618 | P28335 | 132 | Novel |
| DB00502 | P28335 | 131.101 | Novel |
| DB06288 | P21728 | 128 | Novel |
| DB01346 | P34903 | 126.347 | Novel |
| DB01162 | P08913 | 125.291 | Novel |
| DB00590 | P08913 | 125.281 | Novel |
| DB00697 | P35348 | 124.255 | Novel |
| DB00484 | P35348 | 124.216 | Novel |
| DB00346 | P08913 | 123.078 | Novel |
| DB01576 | P08913 | 122.409 | Novel |
| DB00280 | P08173 | 119.868 | Novel |
| DB00388 | P08913 | 118.759 | Novel |
| DB00723 | P08913 | 118.139 | Novel |
| DB00298 | P08913 | 118.072 | Novel |
| DB00706 | P08913 | 118.013 | Novel |
| DB00211 | P08913 | 118.003 | Novel |
| DB06207 | P08913 | 118 | Novel |
| DB00332 | P08173 | 116.619 | Novel |
| DB00462 | P08173 | 116.435 | Novel |
| DB01231 | P08173 | 115.747 | Novel |
| DB00986 | P08173 | 115.686 | Novel |
| DB00383 | P08173 | 115.656 | Novel |
| DB01409 | P08173 | 115.209 | Novel |
| DB01062 | P08173 | 115.183 | Novel |
| DB00517 | P08173 | 115.146 | Novel |
| DB00202 | P08173 | 115.069 | Novel |
| DB01085 | P08173 | 115.069 | Novel |
| DB00193 | P11229 | 114.066 | Novel |
| DB00215 | P28223 | 113.148 | Novel |
| DB00449 | P35368 | 112 | Novel |
| DB00392 | P20309 | 111.391 | Novel |
| DB00865 | P35368 | 111.316 | Novel |
| DB01226 | P11229 | 111 | Novel |
| DB01337 | P11229 | 111 | Novel |
| DB01338 | P11229 | 111 | Novel |
| DB01623 | P35348 | 110.159 | Novel |
| DB04843 | P08172 | 109.508 | Novel |
| DB00411 | P20309 | 109.104 | Novel |
| DB00185 | P08172 | 109 | Novel |
| DB01148 | P20309 | 106.109 | Novel |
| DB00804 | P20309 | 106 | Novel |
| DB00320 | P08908 | 104.017 | Novel |
| DB01579 | P08913 | 104.005 | Novel |
| DB00831 | P28223 | 98.253 | Novel |
| DB00571 | P08913 | 98.006 | Novel |
| DB08807 | P08913 | 98.001 | Novel |
| DB06711 | P35368 | 97.089 | Novel |
| DB00692 | P35368 | 97.004 | Novel |
| DB00850 | P28223 | 96.282 | Novel |
| DB09016 | P14416 | 95.907 | Novel |
| DB00623 | P28223 | 94.47 | Novel |
| DB01063 | P28223 | 93.398 | Novel |
| DB06716 | P18505 | 92.918 | Novel |
| DB01233 | P28223 | 92.15 | Novel |
| DB01625 | P11229 | 91.875 | Novel |
| DB00450 | P28223 | 91.241 | Novel |
| DB00490 | P28223 | 91.044 | Novel |
| DB08810 | P14416 | 91 | Novel |
| DB01295 | P35368 | 89.185 | Novel |
| DB00421 | Q13936 | 88.221 | Exist |
| DB00960 | P08913 | 88.006 | Novel |
| DB01359 | P08913 | 88.001 | Novel |
| DB00629 | P18825 | 88 | Novel |
| DB01018 | P18825 | 88 | Novel |
| DB01100 | P28223 | 87.429 | Novel |
| DB00933 | P21728 | 87.399 | Novel |
| DB08801 | P11229 | 87.13 | Novel |
| DB00952 | P28223 | 86.001 | Novel |
| DB00315 | P28223 | 86 | Novel |
| DB00669 | P28223 | 86 | Novel |
| DB00245 | P08172 | 85.414 | Novel |
| DB04842 | P21728 | 84.252 | Novel |
| DB00289 | P23975 | 82.821 | Exist |
| DB00366 | P08172 | 81.56 | Novel |
| DB01114 | P35348 | 81.143 | Novel |
| DB01184 | P28223 | 79.93 | Novel |
| DB00391 | P28223 | 79.492 | Novel |
| DB00354 | P08172 | 79.326 | Novel |
| DB01364 | P35368 | 77.725 | Novel |
| DB00904 | P28223 | 76 | Novel |
| DB06707 | P35368 | 75.219 | Novel |
| DB00514 | P35372 | 73.158 | Exist |
| DB09014 | P28223 | 72.449 | Novel |
| DB00604 | P14416 | 72.176 | Novel |
| DB00745 | P35348 | 70.029 | Novel |
| DB01255 | P35348 | 70.009 | Novel |
| DB00866 | P35348 | 68.011 | Novel |
| DB06738 | Q8TCU5 | 68 | Exist |
| DB00898 | P47869 | 67.293 | Novel |
| DB00728 | P11229 | 67 | Novel |
| DB00191 | P23975 | 66.563 | Exist |
| DB01173 | P31645 | 66.458 | Novel |
| DB00962 | P47869 | 66.293 | Novel |
| DB04896 | Q01959 | 65.35 | Novel |
| DB00661 | Q13936 | 65.221 | Exist |
| DB01023 | Q13936 | 65.185 | Exist |
| DB01043 | P28223 | 65 | Novel |
| DB01407 | P07550 | 64.782 | Exist |
| DB00810 | P08172 | 64.6 | Novel |
| DB01064 | P07550 | 64.58 | Exist |
| DB00953 | P08908 | 64.195 | Novel |
| DB06204 | P35372 | 64.166 | Exist |
| DB01104 | P23975 | 63.56 | Novel |
| DB00422 | P23975 | 63.464 | Exist |
| DB06701 | P23975 | 63.464 | Exist |
| DB08808 | P08588 | 63.362 | Exist |
| DB01102 | P07550 | 63.171 | Exist |
| DB01288 | P07550 | 63.157 | Exist |
| DB00285 | P23975 | 62.923 | Exist |
| DB01054 | Q13936 | 62.718 | Exist |
| DB00476 | P23975 | 62.533 | Exist |
| DB00579 | P23975 | 62.347 | Exist |
| DB01105 | P23975 | 62.221 | Exist |
| DB01156 | P31645 | 61.055 | Novel |
| DB00940 | P08172 | 61.04 | Novel |
| DB06706 | P35368 | 61 | Novel |
| DB00937 | P31645 | 60.33 | Novel |
| DB00830 | P31645 | 60.109 | Novel |
| DB00996 | Q12879 | 60 | Exist |
| DB01154 | P47869 | 59.493 | Novel |
| DB00483 | P11229 | 59 | Novel |
| DB00610 | P35368 | 58.993 | Novel |
| DB00393 | Q13936 | 58.984 | Exist |
| DB00270 | Q13936 | 58.701 | Exist |
| DB00474 | P47869 | 58.612 | Novel |
| DB01115 | Q13936 | 58.152 | Exist |
| DB01253 | P35368 | 58.068 | Novel |
| DB00699 | P35368 | 58 | Novel |
| DB01135 | P11229 | 58 | Novel |
| DB01191 | P28223 | 58 | Novel |
| DB01336 | P11229 | 58 | Novel |
| DB00704 | P35372 | 57.999 | Exist |
| DB00433 | P28223 | 57.842 | Novel |
| DB00327 | P35372 | 57.821 | Exist |
| DB00497 | P35372 | 57.748 | Exist |
| DB00942 | P08172 | 57.524 | Novel |
| DB00837 | P47869 | 57.293 | Novel |
| DB00795 | P35354 | 57.259 | Exist |
| DB00244 | P35354 | 57.083 | Exist |
| DB00782 | P08172 | 56.73 | Novel |
| DB01183 | P35372 | 56.71 | Exist |
| DB00979 | P08172 | 56.707 | Novel |
| DB00219 | P08172 | 56.677 | Novel |
| DB00771 | P08172 | 56.597 | Novel |
| DB00670 | P08172 | 56.473 | Novel |
| DB00209 | P08172 | 56.454 | Novel |
| DB00333 | P41145 | 56.43 | Novel |
| DB01019 | P11229 | 56.196 | Novel |
| DB00318 | P35372 | 56.161 | Exist |
| DB00295 | P35372 | 56.071 | Exist |
| DB00431 | P14867 | 56 | Novel |
| DB00924 | P14416 | 55.803 | Novel |
| DB00918 | P08908 | 55.452 | Novel |
| DB00844 | P35372 | 55.323 | Exist |
| DB00854 | P35372 | 55.322 | Exist |
| DB00381 | Q13936 | 55.185 | Exist |
| DB00998 | P08908 | 55 | Novel |
| DB01192 | P41145 | 54.539 | Novel |
| DB00611 | P35372 | 54.439 | Exist |
| DB00653 | Q01668 | 54.181 | Novel |
| DB01452 | P35372 | 54.023 | Exist |
| DB00647 | P35372 | 53.964 | Exist |
| DB00813 | P35372 | 53.9 | Exist |
| DB00708 | P35372 | 53.819 | Exist |
| DB00956 | P41145 | 53.586 | Novel |
| DB06709 | P08172 | 53.545 | Novel |
| DB00921 | P35372 | 53.513 | Exist |
| DB00836 | P35372 | 53.48 | Exist |
| DB00899 | P35372 | 53.457 | Exist |
| DB06800 | P41143 | 53.354 | Novel |
| DB06274 | P35372 | 53.277 | Exist |
| DB00729 | P11229 | 53.199 | Novel |
| DB00843 | P14416 | 53.051 | Novel |
| DB06712 | Q13936 | 53.04 | Exist |
| DB00915 | P28223 | 53 | Novel |
| DB01014 | P35354 | 52.34 | Exist |
| DB00652 | P41143 | 52.314 | Novel |
| DB01425 | P28223 | 52.176 | Novel |
| DB00713 | Q75Y35 | 52.065 | Exist |
| DB09018 | P28223 | 52.006 | Novel |
| DB04844 | P28223 | 52 | Novel |
| DB00344 | Q01959 | 51.9 | Novel |
| DB00586 | P23219 | 51.744 | Exist |
| DB06700 | Q01959 | 51.704 | Novel |
| DB08918 | Q01959 | 51.35 | Novel |
| DB00401 | Q13936 | 51.33 | Exist |
| DB00233 | P23219 | 51.026 | Novel |
| DB00489 | P35348 | 51.01 | Novel |
| DB00939 | P35354 | 50.77 | Exist |
| DB04948 | P35348 | 50.536 | Novel |
| DB00968 | P35348 | 50.469 | Novel |
| DB01209 | P41143 | 50.468 | Novel |
| DB01081 | P41145 | 50.387 | Novel |
| DB00456 | Q75Y35 | 50.129 | Exist |
| DB00633 | P35348 | 50.003 | Novel |
| DB06623 | P35348 | 50 | Novel |
| DB00415 | P0A3M6 | 49.789 | Exist |
| DB00328 | P35354 | 49.651 | Exist |
| DB00493 | Q75Y35 | 48.932 | Novel |
| DB01331 | Q8DR59 | 48.92 | Exist |
| DB00567 | P0A3M6 | 48.857 | Exist |
| DB00319 | Q8DR59 | 48.812 | Novel |
| DB08795 | P0A3M6 | 48.69 | Exist |
| DB00264 | P08588 | 48.468 | Exist |
| DB00485 | P0A3M6 | 48.381 | Exist |
| DB00159 | P23219 | 48.348 | Exist |
| DB00711 | P35354 | 48.134 | Novel |
| DB01001 | P07550 | 48.112 | Exist |
| DB01140 | Q8DNB6 | 48.073 | Novel |
| DB01118 | P07550 | 48.001 | Novel |
| DB00335 | P08588 | 47.792 | Exist |
| DB00607 | P0A3M6 | 47.78 | Exist |
| DB01580 | P08588 | 47.727 | Exist |
| DB01297 | P07550 | 47.702 | Novel |
| DB01603 | P0A3M6 | 47.618 | Exist |
| DB00187 | P07550 | 47.569 | Novel |
| DB00841 | P07550 | 47.531 | Exist |
| DB00592 | P47870 | 47.431 | Novel |
| DB00602 | P47870 | 47.431 | Novel |
| DB01291 | P07550 | 47.43 | Exist |
| DB00221 | P07550 | 47.404 | Exist |
| DB00195 | P08588 | 47.316 | Exist |
| DB01050 | P35354 | 47.245 | Exist |
| DB00774 | P00918 | 47.207 | Exist |
| DB01210 | P08588 | 47.194 | Exist |
| DB00816 | P08588 | 47.186 | Novel |
| DB00871 | P08588 | 47.186 | Novel |
| DB00612 | P08588 | 47.164 | Exist |
| DB00521 | P08588 | 47.093 | Exist |
| DB00667 | P35348 | 47 | Novel |
| DB01440 | P14867 | 47 | Novel |
| DB06151 | O60391 | 47 | Novel |
| DB00867 | P08588 | 46.844 | Novel |
| DB01203 | P07550 | 46.837 | Exist |
| DB01193 | P08588 | 46.787 | Exist |
| DB00983 | P08588 | 46.722 | Novel |
| DB01274 | P08588 | 46.722 | Novel |
| DB00739 | P0A3M6 | 46.634 | Exist |
| DB00945 | P35354 | 46.634 | Exist |
| DB00999 | P00918 | 46.56 | Exist |
| DB00353 | P14416 | 46.503 | Novel |
| DB01366 | P08588 | 46.261 | Novel |
| DB00721 | P23975 | 46.2 | Novel |
| DB00938 | P08588 | 46.18 | Novel |
| DB00201 | P27815 | 46.164 | Exist |
| DB01214 | P08588 | 46.133 | Exist |
| DB00373 | P08588 | 46.123 | Exist |
| DB01408 | P08588 | 46.003 | Novel |
| DB05039 | P08588 | 46.001 | Novel |
| DB04861 | P08588 | 46 | Exist |
| DB01009 | P23219 | 44.97 | Exist |
| DB00936 | P35354 | 44.671 | Exist |
| DB00573 | P35354 | 44.544 | Exist |
| DB00909 | P00918 | 44.415 | Exist |
| DB00605 | P23219 | 44.267 | Exist |
| DB00712 | P35354 | 44.147 | Exist |
| DB00870 | P23219 | 44.013 | Exist |
| DB01600 | P35354 | 44.01 | Exist |
| DB04552 | P35354 | 43.649 | Exist |
| DB01146 | P35348 | 43.644 | Novel |
| DB00784 | P35354 | 43.631 | Exist |
| DB01283 | P35354 | 43.575 | Exist |
| DB01398 | P35354 | 43.572 | Exist |
| DB00963 | P23219 | 43.38 | Exist |
| DB00821 | P23219 | 43.356 | Exist |
| DB00500 | P23219 | 43.202 | Exist |
| DB01041 | P23219 | 43.157 | Novel |
| DB00788 | P35354 | 43.138 | Exist |
| DB00861 | P35354 | 43.068 | Exist |
| DB00469 | P23219 | 42.773 | Exist |
| DB00465 | P23219 | 42.766 | Exist |
| DB01399 | P35354 | 42.718 | Exist |
| DB06725 | P23219 | 42.708 | Exist |
| DB01397 | P35354 | 42.666 | Exist |
| DB00554 | P35354 | 42.525 | Exist |
| DB00814 | P23219 | 42.451 | Exist |
| DB00991 | P35354 | 42.394 | Exist |
| DB00562 | P00918 | 42.37 | Exist |
| DB00154 | P23219 | 42.348 | Exist |
| DB06802 | P23219 | 42.334 | Exist |
| DB04920 | Q08289 | 42.282 | Novel |
| DB00461 | P35354 | 42.262 | Exist |
| DB01401 | P35354 | 42.206 | Exist |
| DB01628 | P23219 | 42.193 | Novel |
| DB08910 | P23219 | 42.169 | Novel |
| DB00480 | P23219 | 42.157 | Novel |
| DB00637 | P35348 | 42.144 | Novel |
| DB00482 | P23219 | 42.136 | Novel |
| DB00316 | P35354 | 42.135 | Exist |
| DB00812 | P23219 | 42.135 | Exist |
| DB00350 | P35354 | 42.134 | Novel |
| DB00749 | P23219 | 42.134 | Exist |
| DB01419 | P23219 | 42.134 | Exist |
| DB01435 | P23219 | 42.134 | Exist |
| DB06594 | P28223 | 42 | Novel |
| DB01329 | P02918 | 41.391 | Exist |
| DB01161 | P23975 | 40.196 | Novel |
| DB00436 | P00918 | 39.671 | Exist |
| DB01616 | P28223 | 39.106 | Novel |
| DB01246 | P28223 | 39.105 | Novel |
| DB06684 | P28223 | 39.094 | Novel |
| DB01021 | P00918 | 38.568 | Exist |
| DB01234 | P04150 | 38.321 | Exist |
| DB01119 | P22748 | 38 | Novel |
| DB04841 | P14416 | 37.11 | Novel |
| DB00880 | P00918 | 36.862 | Exist |
| DB00819 | P00918 | 36.486 | Exist |
| DB00472 | P23975 | 36.386 | Novel |
| DB01325 | P22748 | 36.351 | Novel |
| DB00443 | P04150 | 36.321 | Exist |
| DB00234 | P31645 | 36.278 | Novel |
| DB00232 | P00918 | 36.155 | Exist |
| DB00674 | P43681 | 36.058 | Exist |
| DB00176 | P23975 | 36 | Novel |
| DB00226 | P31645 | 36 | Novel |
| DB01170 | P31645 | 36 | Novel |
| DB04840 | P31645 | 36 | Novel |
| DB00184 | P43681 | 36 | Exist |
| DB00303 | P02919 | 36 | Exist |
| DB01615 | P28223 | 35.8 | Novel |
| DB08802 | P28223 | 35.584 | Novel |
| DB00651 | Q07343 | 34.802 | Exist |
| DB08896 | P10721 | 34.659 | Exist |
| DB00902 | P28223 | 34.494 | Novel |
| DB00703 | P00918 | 34.388 | Exist |
| DB01144 | P00918 | 34.388 | Exist |
| DB00308 | Q13698 | 34.067 | Novel |
| DB00145 | Q8TCU5 | 34 | Novel |
| DB00949 | Q05586 | 34 | Novel |
| DB01620 | P28223 | 33.751 | Novel |
| DB00768 | P28223 | 33.7 | Novel |
| DB04837 | P28223 | 33.66 | Novel |
| DB00405 | P11229 | 33.61 | Novel |
| DB01075 | P28223 | 33.18 | Novel |
| DB00792 | P28223 | 33.161 | Novel |
| DB08800 | P28223 | 32.922 | Novel |
| DB01071 | P28223 | 32.909 | Novel |
| DB00748 | P28223 | 32.855 | Novel |
| DB01237 | P28223 | 32.674 | Novel |
| DB06691 | P28223 | 32.63 | Novel |
| DB08936 | P28223 | 32.552 | Novel |
| DB01328 | P02918 | 32.393 | Exist |
| DB01194 | P00918 | 32.384 | Exist |
| DB00719 | P28223 | 32.382 | Novel |
| DB00438 | P02918 | 32.362 | Exist |
| DB06698 | P35348 | 32.29 | Novel |
| DB01176 | P28223 | 32.256 | Novel |
| DB00557 | P28223 | 32.197 | Novel |
| DB01619 | P28223 | 31.956 | Novel |
| DB00920 | P28223 | 31.808 | Novel |
| DB00283 | P28223 | 31.739 | Novel |
| DB00341 | P28223 | 31.669 | Novel |
| DB00606 | P00918 | 31.658 | Exist |
| DB08799 | P35348 | 31.577 | Novel |
| DB00972 | P35348 | 31.576 | Novel |
| DB00967 | P28223 | 31.54 | Novel |
| DB00737 | P28223 | 31.481 | Novel |
| DB00455 | P28223 | 31.439 | Novel |
| DB00869 | P00918 | 31.384 | Exist |
| DB04890 | P28223 | 31.297 | Novel |
| DB06766 | P28223 | 31.242 | Novel |
| DB00427 | P35348 | 31.2 | Novel |
| DB01084 | P28223 | 31.18 | Novel |
| DB00950 | P28223 | 31.094 | Novel |
| DB00985 | P28223 | 31.025 | Novel |
| DB01106 | P28223 | 31.006 | Novel |
| DB00430 | P02918 | 30.48 | Exist |
| DB01227 | P41143 | 29.861 | Novel |
| DB01466 | P41143 | 29.405 | Novel |
| DB00764 | P04150 | 29.317 | Exist |
| DB00274 | P02918 | 29.241 | Exist |
| DB00223 | P04150 | 29.229 | Exist |
| DB00277 | P27815 | 29.049 | Exist |
| DB01031 | P22748 | 29 | Novel |
| DB00620 | P04150 | 28.833 | Exist |
| DB00504 | P41145 | 28.535 | Novel |
| DB01415 | P02918 | 28.465 | Exist |
| DB01327 | P02919 | 28.38 | Exist |
| DB01384 | P04150 | 28.332 | Exist |
| DB00396 | P35372 | 28.077 | Novel |
| DB08901 | P09619 | 28.068 | Novel |
| DB01273 | P14867 | 28 | Novel |
| DB09028 | P14867 | 28 | Novel |
| DB01433 | P41143 | 27.861 | Novel |
| DB00398 | P16234 | 27.659 | Novel |
| DB00913 | P41143 | 27.478 | Novel |
| DB01463 | P23975 | 27.321 | Novel |
| DB00802 | P41143 | 27.311 | Novel |
| DB01000 | P0A3M6 | 27.152 | Novel |
| DB00825 | P35372 | 27.077 | Novel |
| DB01598 | P0AD68 | 27 | Novel |
| DB01303 | Q07343 | 26.632 | Novel |
| DB00547 | P04150 | 25.982 | Exist |
| DB01059 | P43700 | 25.275 | Exist |
| DB00487 | P43700 | 25.141 | Exist |
| DB01137 | P43700 | 25.127 | Exist |
| DB01165 | P43700 | 25.127 | Exist |
| DB00467 | P43700 | 24.975 | Exist |
| DB04570 | P0AD68 | 24.63 | Novel |
| DB00978 | P43700 | 24.472 | Exist |
| DB00537 | P43700 | 24.356 | Exist |
| DB05266 | Q07343 | 23.3 | Exist |
| DB00240 | P04150 | 23.16 | Exist |
| DB00218 | P43700 | 23.053 | Exist |
| DB08954 | Q12879 | 23.001 | Novel |
| DB01268 | P10721 | 23 | Exist |
| DB01208 | P43700 | 22.749 | Exist |
| DB01332 | P02918 | 22.631 | Exist |
| DB00824 | P30542 | 22.515 | Exist |
| DB00838 | P04150 | 22.482 | Exist |
| DB00806 | P27815 | 22.424 | Exist |
| DB01412 | P27815 | 22.229 | Novel |
| DB00860 | P04150 | 22.011 | Exist |
| DB06771 | P11388 | 21.992 | Novel |
| DB08970 | P04150 | 21.819 | Novel |
| DB01155 | P11388 | 21.359 | Novel |
| DB08971 | P04150 | 21.315 | Novel |
| DB00685 | P43700 | 21.241 | Exist |
| DB00695 | P00915 | 21.159 | Novel |
| DB06589 | P10721 | 21 | Exist |
| DB01147 | Q75Y35 | 20.747 | Novel |
| DB00313 | Q14524 | 20.641 | Exist |
| DB01414 | P0AD68 | 20.362 | Novel |
| DB00523 | P28702 | 20.357 | Exist |
| DB01088 | Q07343 | 20.3 | Exist |
| DB01656 | Q07343 | 20.3 | Exist |
| DB01244 | Q13936 | 20.003 | Novel |
| DB00959 | P04150 | 19.894 | Exist |
| DB00687 | P04150 | 19.253 | Exist |
| DB00833 | Q8DR59 | 19.171 | Novel |
| DB01413 | P02918 | 18.788 | Novel |
| DB00459 | P28702 | 18.378 | Exist |
| DB00210 | P28702 | 18.204 | Exist |
| DB01199 | P08172 | 18.102 | Novel |
| DB01185 | P06401 | 17.886 | Novel |
| DB00948 | Q8DR59 | 17.793 | Novel |
| DB01013 | P04150 | 17.649 | Exist |
| DB00180 | P04150 | 17.416 | Exist |
| DB01066 | Q75Y35 | 17.352 | Novel |
| DB00591 | P04150 | 17.351 | Exist |
| DB00423 | P00918 | 17.107 | Novel |
| DB00619 | P35968 | 17.068 | Novel |
| DB01110 | P35348 | 17 | Novel |
| DB08893 | P08588 | 17 | Novel |
| DB00588 | P04150 | 16.804 | Exist |
| DB00896 | P04150 | 16.72 | Exist |
| DB00367 | P06401 | 16.611 | Exist |
| DB01260 | P04150 | 16.429 | Exist |
| DB00596 | P04150 | 16.224 | Exist |
| DB08811 | Q07343 | 16.182 | Novel |
| DB00324 | P04150 | 15.768 | Exist |
| DB00755 | P10276 | 15.654 | Novel |
| DB00640 | Q07343 | 15.459 | Novel |
| DB00741 | P04150 | 15.391 | Exist |
| DB00131 | P27815 | 15.14 | Novel |
| DB00799 | P48443 | 15.087 | Novel |
| DB01223 | P29274 | 15.052 | Novel |
| DB01254 | P16234 | 15 | Novel |
| DB06751 | Q01668 | 14.109 | Novel |
| DB00975 | Q07343 | 14.078 | Novel |
| DB01427 | P27815 | 14.078 | Novel |
| DB01212 | Q7CRA4 | 13.35 | Novel |
| DB00663 | P04150 | 13.138 | Exist |
| DB01330 | Q8DR59 | 13.065 | Novel |
| DB01395 | P03372 | 13.02 | Novel |
| DB01333 | P02919 | 13.007 | Novel |
| DB01222 | P04150 | 12.865 | Exist |
| DB01406 | P06401 | 12.778 | Exist |
| DB00827 | P43702 | 12.465 | Novel |
| DB01113 | P27815 | 12.183 | Novel |
| DB00126 | O15460 | 12.143 | Novel |
| DB00139 | P13674 | 12.143 | Exist |
| DB00757 | P14416 | 12.05 | Novel |
| DB01047 | P04150 | 12.04 | Exist |
| DB00294 | P06401 | 12.034 | Exist |
| DB00307 | P13631 | 12.024 | Novel |
| DB00204 | P35348 | 12.001 | Novel |
| DB00377 | P14416 | 12 | Novel |
| DB00889 | P14416 | 12 | Novel |
| DB00969 | P14416 | 12 | Novel |
| DB00717 | P03372 | 11.868 | Novel |
| DB00783 | P03372 | 11.766 | Exist |
| DB00394 | P04150 | 11.6 | Exist |
| DB00304 | P06401 | 11.481 | Exist |
| DB00635 | P04150 | 11.473 | Exist |
| DB01416 | P02918 | 11.46 | Novel |
| DB06218 | Q14524 | 11.315 | Novel |
| DB01182 | P35348 | 11.3 | Novel |
| DB00267 | P02918 | 11.282 | Novel |
| DB00417 | Q75Y35 | 11.173 | Novel |
| DB06713 | P03372 | 11.139 | Novel |
| DB01245 | P06276 | 11.06 | Novel |
| DB00276 | P35348 | 11 | Novel |
| DB00908 | P35348 | 11 | Novel |
| DB01218 | P35348 | 11 | Novel |
| DB04838 | Q13936 | 11 | Novel |
| DB01037 | P27338 | 10.982 | Exist |
| DB00351 | P03372 | 10.927 | Novel |
| DB00780 | P27338 | 10.883 | Exist |
| DB01626 | P27338 | 10.807 | Exist |
| DB00846 | P04150 | 10.633 | Exist |
| DB00957 | P06401 | 10.582 | Exist |
| DB00752 | P27338 | 10.473 | Exist |
| DB00603 | P06401 | 10.427 | Exist |
| DB01171 | P27338 | 10.27 | Novel |
| DB01367 | P21397 | 10.268 | Novel |
| DB01247 | P27338 | 10.268 | Exist |
| DB03147 | P27338 | 10.268 | Exist |
| DB00977 | Q92731 | 10.165 | Novel |
| DB00823 | P06401 | 10.131 | Exist |
| DB00288 | P04150 | 10.119 | Exist |
| DB01431 | P06401 | 10.092 | Exist |
| DB00834 | P03372 | 10.065 | Novel |
| DB04574 | P03372 | 10.021 | Exist |
| DB00380 | P43700 | 10 | Novel |
| DB00385 | P43700 | 10 | Novel |
| DB00444 | P43700 | 10 | Novel |
| DB00445 | P43700 | 10 | Novel |
| DB00694 | P43700 | 10 | Novel |
| DB00773 | P43700 | 10 | Novel |
| DB00970 | P43700 | 10 | Novel |
| DB00997 | P43700 | 10 | Novel |
| DB01090 | P43681 | 10 | Novel |
| DB01177 | P43700 | 10 | Novel |
| DB01178 | P14867 | 10 | Novel |
| DB01179 | P43700 | 10 | Novel |
| DB01204 | P43700 | 10 | Novel |
| DB04967 | P43700 | 10 | Novel |
| DB00378 | P03372 | 9.966 | Novel |
| DB04573 | P03372 | 9.918 | Exist |
| DB01016 | Q09428 | 9.751 | Exist |
| DB00675 | P03372 | 9.723 | Exist |
| DB00595 | P0A7X3 | 9.6 | Exist |
| DB01400 | P06276 | 9.545 | Novel |
| DB01393 | P23219 | 9.457 | Novel |
| DB01511 | P47869 | 9.443 | Novel |
| DB00989 | P22303 | 9.385 | Exist |
| DB00254 | P0A7X3 | 9.354 | Exist |
| DB00545 | P22303 | 9.347 | Exist |
| DB00655 | Q92731 | 9.333 | Novel |
| DB01010 | P22303 | 9.234 | Exist |
| DB01357 | P06401 | 9.176 | Novel |
| DB00286 | Q92731 | 9.146 | Novel |
| DB04575 | P06401 | 9.142 | Novel |
| DB01060 | Q75Y35 | 9.12 | Novel |
| DB06781 | P04150 | 9.093 | Exist |
| DB01108 | P06401 | 9.077 | Novel |
| DB00981 | P06276 | 9.062 | Novel |
| DB00772 | P22303 | 9.06 | Novel |
| DB00941 | P22303 | 9.06 | Novel |
| DB01057 | P22303 | 9.06 | Novel |
| DB01122 | P06276 | 9.06 | Novel |
| DB08996 | P06276 | 9.06 | Novel |
| DB00122 | P22303 | 9.06 | Exist |
| DB00677 | P22303 | 9.06 | Exist |
| DB00733 | P22303 | 9.06 | Exist |
| DB00944 | P22303 | 9.06 | Exist |
| DB04868 | P09619 | 9.052 | Novel |
| DB01196 | P06401 | 9.001 | Novel |
| DB00172 | Q02809 | 9 | Novel |
| DB00255 | P06401 | 9 | Novel |
| DB00481 | P06401 | 9 | Novel |
| DB00539 | P06401 | 9 | Novel |
| DB00882 | P06401 | 9 | Novel |
| DB00890 | P06401 | 9 | Novel |
| DB00947 | P06401 | 9 | Novel |
| DB01065 | P06401 | 9 | Novel |
| DB04938 | P06401 | 9 | Novel |
| DB06616 | P10721 | 9 | Novel |
| DB00453 | P0A7X3 | 8.966 | Exist |
| DB00618 | P0A7X3 | 8.949 | Exist |
| DB01339 | P08172 | 8.892 | Novel |
| DB01017 | P0A7X3 | 8.718 | Exist |
| DB00982 | P13631 | 8.574 | Novel |
| DB00281 | Q14524 | 8.523 | Exist |
| DB04942 | P13631 | 8.491 | Novel |
| DB00732 | P08172 | 8.43 | Novel |
| DB00222 | Q15842 | 8.308 | Novel |
| DB00301 | Q75Y35 | 8.234 | Novel |
| DB00243 | Q9Y5Y9 | 8.204 | Novel |
| DB00873 | P04150 | 8.175 | Exist |
| DB06213 | P30542 | 8.172 | Novel |
| DB01301 | P0A7V8 | 8.135 | Novel |
| DB00565 | P08172 | 8.079 | Novel |
| DB00272 | P35367 | 8 | Novel |
| DB00358 | P30542 | 8 | Novel |
| DB00501 | P35367 | 8 | Novel |
| DB00585 | P35367 | 8 | Novel |
| DB00657 | P08172 | 8 | Novel |
| DB00863 | P35367 | 8 | Novel |
| DB00903 | P00918 | 8 | Novel |
| DB00927 | P35367 | 8 | Novel |
| DB06714 | P35348 | 8 | Novel |
| DB08806 | P35367 | 8 | Novel |
| DB00129 | P30825 | 7.58 | Exist |
| DB01130 | P04150 | 7.564 | Exist |
| DB00115 | Q99707 | 7.529 | Exist |
| DB00125 | P52569 | 7.498 | Novel |
| DB00123 | P78540 | 7.469 | Novel |
| DB01061 | Q75Y35 | 7.378 | Novel |
| DB00256 | P0A7X3 | 7.363 | Exist |
| DB06710 | P06401 | 7.234 | Novel |
| DB00473 | Q15858 | 7.156 | Novel |
| DB00527 | Q15858 | 7.156 | Novel |
| DB00759 | P0A7X3 | 7.104 | Novel |
| DB00412 | P23219 | 7.07 | Novel |
| DB00171 | P48048 | 7 | Novel |
| DB00206 | P35348 | 7 | Novel |
| DB06637 | P20309 | 7 | Novel |
| DB01248 | Q9H4B7 | 6.953 | Exist |
| DB01429 | P14416 | 6.928 | Novel |
| DB08906 | P04150 | 6.917 | Exist |
| DB00560 | P0A7V8 | 6.91 | Novel |
| DB01324 | P00918 | 6.864 | Novel |
| DB00624 | P06401 | 6.854 | Novel |
| DB01229 | Q9H4B7 | 6.597 | Exist |
| DB00252 | Q15858 | 6.529 | Novel |
| DB00200 | Q99707 | 6.529 | Exist |
| DB01139 | Q75Y35 | 6.422 | Novel |
| DB01251 | Q14654 | 6.393 | Novel |
| DB09000 | P35367 | 6.358 | Novel |
| DB01112 | Q75Y35 | 6.327 | Novel |
| DB00451 | P10828 | 6.291 | Exist |
| DB00509 | P10828 | 6.291 | Exist |
| DB00524 | P00918 | 6.206 | Novel |
| DB01195 | Q15858 | 6.157 | Novel |
| DB00279 | P10828 | 6.079 | Exist |
| DB00887 | P00918 | 6.059 | Novel |
| DB01132 | P23219 | 6.047 | Novel |
| DB00912 | P23219 | 6.001 | Novel |
| DB00163 | P23219 | 6 | Novel |
| DB00179 | P23219 | 6 | Novel |
| DB00471 | P23219 | 6 | Novel |
| DB00731 | P23219 | 6 | Novel |
| DB00744 | P23219 | 6 | Novel |
| DB00761 | P00918 | 6 | Novel |
| DB00966 | P23219 | 6 | Novel |
| DB01067 | P23219 | 6 | Novel |
| DB01089 | P35348 | 6 | Novel |
| DB01252 | P23219 | 6 | Novel |
| DB01044 | P43700 | 5.964 | Novel |
| DB00769 | P04150 | 5.894 | Exist |
| DB01420 | P06401 | 5.857 | Novel |
| DB01035 | Q9Y5Y9 | 5.603 | Novel |
| DB01056 | Q9Y5Y9 | 5.579 | Novel |
| DB01213 | P00326 | 5.564 | Exist |
| DB00035 | P37288 | 5.532 | Exist |
| DB00253 | P06401 | 5.502 | Novel |
| DB00750 | Q9Y5Y9 | 5.459 | Novel |
| DB00297 | Q14524 | 5.436 | Novel |
| DB01002 | Q14524 | 5.436 | Novel |
| DB00296 | Q14524 | 5.431 | Novel |
| DB00961 | Q14524 | 5.356 | Novel |
| DB01602 | Q75Y35 | 5.336 | Novel |
| DB01604 | Q75Y35 | 5.336 | Novel |
| DB02638 | P37288 | 5.282 | Exist |
| DB00347 | Q9P0X4 | 5.272 | Novel |
| DB00617 | O43497 | 5.272 | Novel |
| DB00155 | P00966 | 5.23 | Exist |
| DB01380 | P04150 | 5.228 | Exist |
| DB00892 | Q14524 | 5.208 | Novel |
| DB01228 | Q9Y5Y9 | 5.207 | Novel |
| DB00229 | Q75Y35 | 5.201 | Novel |
| DB00929 | P34995 | 5.195 | Novel |
| DB00192 | Q9Y5Y9 | 5.174 | Novel |
| DB00379 | Q9Y5Y9 | 5.151 | Novel |
| DB00645 | Q14524 | 5.148 | Novel |
| DB00760 | P02919 | 5.132 | Novel |
| DB00807 | Q14524 | 5.115 | Novel |
| DB00564 | Q9Y5Y9 | 5.103 | Novel |
| DB00776 | Q9Y5Y9 | 5.102 | Novel |
| DB01086 | Q14524 | 5.1 | Novel |
| DB01320 | Q9Y5Y9 | 5.098 | Novel |
| DB00680 | Q9Y5Y9 | 5.096 | Novel |
| DB00740 | Q9Y5Y9 | 5.095 | Novel |
| DB00754 | Q9Y5Y9 | 5.095 | Novel |
| DB00868 | Q9Y5Y9 | 5.095 | Novel |
| DB01426 | Q9Y5Y9 | 5.095 | Novel |
| DB01436 | P11473 | 5.048 | Exist |
| DB00310 | P00918 | 5.003 | Novel |
| DB00235 | P27815 | 5.001 | Novel |
| DB01605 | Q75Y35 | 5.001 | Novel |
| DB00214 | P00918 | 5 | Novel |
| DB00261 | P27815 | 5 | Novel |
| DB00534 | P00918 | 5 | Novel |
| DB00593 | Q9P0X4 | 5 | Novel |
| DB00820 | P27815 | 5 | Novel |
| DB00922 | P48048 | 5 | Novel |
| DB01124 | Q14654 | 5 | Novel |
| DB01166 | P27815 | 5 | Novel |
| DB01382 | Q14654 | 5 | Novel |
| DB04880 | P27815 | 5 | Novel |
| DB05246 | Q9P0X4 | 5 | Novel |
| DB01410 | P04150 | 4.975 | Exist |
| DB00146 | P11473 | 4.967 | Exist |
| DB00169 | P11473 | 4.944 | Exist |
| DB01053 | Q75Y35 | 4.796 | Novel |
| DB00136 | P11473 | 4.788 | Exist |
| DB04839 | P06401 | 4.741 | Novel |
| DB00570 | P68371 | 4.634 | Novel |
| DB00770 | P43115 | 4.623 | Novel |
| DB00839 | Q09428 | 4.601 | Novel |
| DB00858 | P06401 | 4.599 | Novel |
| DB00093 | P30518 | 4.594 | Novel |
| DB01551 | P35372 | 4.588 | Novel |
| DB00143 | P08263 | 4.582 | Exist |
| DB00162 | P13631 | 4.581 | Novel |
| DB00917 | P43116 | 4.565 | Exist |
| DB00528 | Q13936 | 4.459 | Novel |
| DB01120 | P48048 | 4.418 | Novel |
| DB01289 | Q14654 | 4.382 | Novel |
| DB08804 | P06401 | 4.372 | Novel |
| DB00672 | P48048 | 4.332 | Novel |
| DB00700 | P06401 | 4.322 | Novel |
| DB00621 | P06401 | 4.264 | Novel |
| DB00160 | P48637 | 4.209 | Novel |
| DB00104 | P35346 | 4.186 | Exist |
| DB00299 | P03176 | 4.177 | Novel |
| DB00984 | P06401 | 4.174 | Novel |
| DB06663 | P35346 | 4.163 | Exist |
| DB00914 | Q14654 | 4.158 | Novel |
| DB00116 | P34897 | 4.144 | Exist |
| DB09002 | P35367 | 4.105 | Novel |
| DB00563 | P04818 | 4.102 | Novel |
| DB06201 | Q14524 | 4.1 | Novel |
| DB01583 | P10828 | 4.092 | Exist |
| DB00631 | P09884 | 4.086 | Exist |
| DB01216 | P31213 | 4.078 | Exist |
| DB04894 | P30872 | 4.071 | Novel |
| DB00872 | P37288 | 4.07 | Exist |
| DB06212 | P37288 | 4.07 | Exist |
| DB00905 | P43116 | 4.061 | Novel |
| DB00128 | P00966 | 4.002 | Exist |
| DB00134 | Q8IVH4 | 4 | Novel |
| DB00203 | P27815 | 4 | Novel |
| DB00257 | Q12791 | 4 | Novel |
| DB00343 | Q13936 | 4 | Novel |
| DB00356 | P00915 | 4 | Novel |
| DB00499 | P06401 | 4 | Novel |
| DB00548 | P18405 | 4 | Novel |
| DB00665 | P06401 | 4 | Novel |
| DB00862 | P27815 | 4 | Novel |
| DB01003 | P00915 | 4 | Novel |
| DB01026 | P06401 | 4 | Novel |
| DB01128 | P06401 | 4 | Novel |
| DB01169 | P23219 | 4 | Novel |
| DB01411 | P35354 | 4 | Novel |
| DB06237 | P27815 | 4 | Novel |
| DB06267 | P27815 | 4 | Novel |
| DB08899 | P06401 | 4 | Novel |
| DB08912 | P35916 | 4 | Novel |
| DB00384 | P51168 | 4 | Exist |
| DB00594 | P51168 | 4 | Exist |
| DB04953 | P23219 | 4 | Exist |
| DB00242 | P09884 | 3.984 | Exist |
| DB06772 | P07437 | 3.968 | Novel |
| DB00518 | P07437 | 3.938 | Novel |
| DB00577 | P03176 | 3.874 | Novel |
| DB00578 | Q75Y35 | 3.869 | Novel |
| DB00178 | P12821 | 3.856 | Exist |
| DB00519 | P12821 | 3.806 | Exist |
| DB01545 | P47869 | 3.802 | Novel |
| DB00120 | P35348 | 3.759 | Novel |
| DB01280 | P23921 | 3.72 | Novel |
| DB00541 | P07437 | 3.705 | Exist |
| DB06813 | P00374 | 3.702 | Exist |
| DB00642 | P04818 | 3.697 | Exist |
| DB01127 | P10613 | 3.69 | Exist |
| DB00584 | P12821 | 3.655 | Exist |
| DB00153 | P11473 | 3.646 | Exist |
| DB00688 | P12268 | 3.626 | Exist |
| DB01024 | P12268 | 3.626 | Exist |
| DB00650 | P00374 | 3.593 | Novel |
| DB01073 | P09884 | 3.586 | Exist |
| DB00132 | P23219 | 3.584 | Novel |
| DB00135 | P00439 | 3.581 | Novel |
| DB01045 | P0A8V2 | 3.563 | Exist |
| DB00811 | P12268 | 3.524 | Novel |
| DB01033 | P12268 | 3.524 | Exist |
| DB00787 | P04293 | 3.501 | Exist |
| DB00293 | P00374 | 3.478 | Novel |
| DB09017 | P47870 | 3.465 | Novel |
| DB00151 | P21549 | 3.454 | Novel |
| DB01421 | P0A7S3 | 3.421 | Novel |
| DB01220 | P0A8T7 | 3.404 | Novel |
| DB01348 | P12821 | 3.404 | Exist |
| DB00643 | F1L7U3 | 3.389 | Novel |
| DB01607 | Q75Y35 | 3.389 | Novel |
| DB05013 | P05771 | 3.386 | Novel |
| DB01007 | P10613 | 3.312 | Exist |
| DB00615 | O75469 | 3.311 | Novel |
| DB00239 | P10613 | 3.308 | Exist |
| DB09021 | P35367 | 3.304 | Novel |
| DB01581 | P0AC13 | 3.277 | Exist |
| DB00174 | P00966 | 3.272 | Novel |
| DB00441 | P09884 | 3.247 | Novel |
| DB00691 | P12821 | 3.231 | Exist |
| DB00309 | P07437 | 3.196 | Novel |
| DB01004 | P09252 | 3.17 | Novel |
| DB00720 | P05141 | 3.151 | Exist |
| DB01157 | P04818 | 3.148 | Novel |
| DB00440 | P00374 | 3.148 | Exist |
| DB01150 | P0A3M6 | 3.139 | Novel |
| DB01121 | Q14524 | 3.117 | Novel |
| DB00188 | P28062 | 3.116 | Novel |
| DB01438 | Q14524 | 3.116 | Novel |
| DB04930 | Q14524 | 3.116 | Novel |
| DB08889 | P28074 | 3.116 | Exist |
| DB00980 | P49286 | 3.089 | Exist |
| DB00555 | P35499 | 3.085 | Novel |
| DB00689 | Q75Y35 | 3.082 | Novel |
| DB01370 | Q13621 | 3.08 | Novel |
| DB01126 | P18405 | 3.073 | Exist |
| DB00559 | P24530 | 3.062 | Exist |
| DB06268 | P24530 | 3.062 | Exist |
| DB08932 | P24530 | 3.062 | Exist |
| DB01375 | P62158 | 3.048 | Novel |
| DB00119 | P24298 | 3.044 | Novel |
| DB01592 | O14732 | 3.027 | Novel |
| DB01593 | O14732 | 3.027 | Novel |
| DB00361 | P68366 | 3.023 | Novel |
| DB00608 | P08588 | 3.001 | Novel |
| DB00205 | P04818 | 3 | Novel |
| DB00227 | P30542 | 3 | Novel |
| DB00266 | P16083 | 3 | Novel |
| DB00322 | P00374 | 3 | Novel |
| DB00360 | P35228 | 3 | Novel |
| DB00390 | P00915 | 3 | Novel |
| DB00426 | P03176 | 3 | Novel |
| DB00432 | P00374 | 3 | Novel |
| DB00468 | Q12791 | 3 | Novel |
| DB00511 | P00915 | 3 | Novel |
| DB00544 | P00374 | 3 | Novel |
| DB00995 | P23219 | 3 | Novel |
| DB01005 | P09884 | 3 | Novel |
| DB01078 | P00915 | 3 | Novel |
| DB01092 | P00915 | 3 | Novel |
| DB01101 | P00374 | 3 | Novel |
| DB01116 | P30926 | 3 | Novel |
| DB01131 | P04818 | 3 | Novel |
| DB01158 | P00915 | 3 | Novel |
| DB01188 | P00915 | 3 | Novel |
| DB01202 | Q13936 | 3 | Novel |
| DB01275 | Q32P28 | 3 | Novel |
| DB01296 | P08588 | 3 | Novel |
| DB01356 | P02787 | 3 | Novel |
| DB01394 | P68366 | 3 | Novel |
| DB01396 | P00915 | 3 | Novel |
| DB01430 | P00915 | 3 | Novel |
| DB02546 | P30542 | 3 | Novel |
| DB08881 | P04049 | 3 | Novel |
| DB00170 | P15559 | 3 | Exist |
| DB00576 | P0AC13 | 2.997 | Exist |
| DB01160 | P34995 | 2.996 | Novel |
| DB01582 | P0AC13 | 2.988 | Exist |
| DB01015 | P0AC13 | 2.979 | Exist |
| DB00994 | P0A7S3 | 2.948 | Exist |
| DB00452 | P0A7S3 | 2.946 | Exist |
| DB00881 | P12821 | 2.895 | Exist |
| DB01070 | P11473 | 2.884 | Exist |
| DB08943 | P10613 | 2.86 | Novel |
| DB00199 | P60725 | 2.854 | Exist |
| DB00207 | P60725 | 2.843 | Exist |
| DB00864 | P42345 | 2.798 | Novel |
| DB00910 | P11473 | 2.745 | Exist |
| DB00337 | P62942 | 2.738 | Exist |
| DB01489 | P18505 | 2.722 | Novel |
| DB08998 | P35367 | 2.686 | Novel |
| DB00671 | P0AD68 | 2.67 | Novel |
| DB00014 | P30968 | 2.664 | Exist |
| DB01077 | P14324 | 2.654 | Novel |
| DB01211 | P61177 | 2.625 | Novel |
| DB00359 | Q27738 | 2.595 | Exist |
| DB01153 | P10613 | 2.581 | Exist |
| DB06147 | Q27738 | 2.578 | Exist |
| DB00765 | P17735 | 2.572 | Novel |
| DB00161 | O15382 | 2.528 | Novel |
| DB00644 | P22888 | 2.516 | Novel |
| DB00684 | P0A7S3 | 2.501 | Exist |
| DB01109 | P01008 | 2.459 | Exist |
| DB00167 | P54687 | 2.456 | Exist |
| DB00149 | P54687 | 2.444 | Exist |
| DB00722 | P12821 | 2.443 | Exist |
| DB00407 | P00742 | 2.435 | Novel |
| DB00050 | P30968 | 2.434 | Exist |
| DB01172 | P0A7S3 | 2.427 | Exist |
| DB05245 | Q27738 | 2.419 | Novel |
| DB02300 | P11473 | 2.416 | Exist |
| DB00133 | Q9Y697 | 2.409 | Novel |
| DB00877 | P42345 | 2.399 | Exist |
| DB00263 | P0AC13 | 2.393 | Exist |
| DB00144 | P05771 | 2.386 | Novel |
| DB08911 | Q02750 | 2.381 | Exist |
| DB00891 | Q27738 | 2.357 | Novel |
| DB06730 | P06401 | 2.357 | Novel |
| DB08798 | P0AC13 | 2.315 | Novel |
| DB00664 | Q27738 | 2.302 | Exist |
| DB01590 | P62942 | 2.294 | Novel |
| DB00479 | P0A7S3 | 2.29 | Exist |
| DB00666 | P22888 | 2.272 | Novel |
| DB04794 | O15554 | 2.272 | Novel |
| DB00355 | P02919 | 2.216 | Novel |
| DB00091 | P62937 | 2.21 | Exist |
| DB00429 | P43116 | 2.205 | Novel |
| DB00331 | O43741 | 2.204 | Novel |
| DB06696 | P0A7S3 | 2.202 | Exist |
| DB08916 | P00533 | 2.196 | Exist |
| DB05294 | P00519 | 2.192 | Novel |
| DB00630 | P38606 | 2.186 | Exist |
| DB01240 | P43119 | 2.175 | Exist |
| DB00762 | Q969P6 | 2.169 | Exist |
| DB01030 | P11387 | 2.169 | Exist |
| DB06699 | P22888 | 2.16 | Novel |
| DB00470 | P21554 | 2.16 | Exist |
| DB00486 | P21554 | 2.16 | Exist |
| DB01055 | P34897 | 2.154 | Novel |
| DB06287 | P62942 | 2.15 | Novel |
| DB01039 | P37231 | 2.135 | Novel |
| DB00569 | P01008 | 2.131 | Exist |
| DB00317 | P04626 | 2.121 | Novel |
| DB05630 | Q969P6 | 2.121 | Novel |
| DB01167 | P10613 | 2.119 | Novel |
| DB00491 | P14410 | 2.114 | Novel |
| DB00284 | O43451 | 2.114 | Exist |
| DB00582 | O15554 | 2.104 | Novel |
| DB01241 | P37231 | 2.103 | Novel |
| DB01265 | Q72547 | 2.1 | Novel |
| DB00709 | Q72547 | 2.096 | Exist |
| DB00946 | P15559 | 2.094 | Novel |
| DB01298 | P0AC13 | 2.084 | Exist |
| DB00682 | P15559 | 2.074 | Novel |
| DB00542 | Q9BYF1 | 2.071 | Novel |
| DB00196 | O15554 | 2.07 | Novel |
| DB00374 | P37231 | 2.067 | Novel |
| DB00230 | Q00975 | 2.065 | Novel |
| DB00636 | P37231 | 2.059 | Novel |
| DB00561 | O14649 | 2.059 | Exist |
| DB01125 | P02818 | 2.056 | Novel |
| DB00338 | P20648 | 2.056 | Exist |
| DB00498 | P15559 | 2.046 | Novel |
| DB00529 | P09252 | 2.045 | Novel |
| DB00943 | Q72547 | 2.018 | Exist |
| DB00790 | Q9BYF1 | 2.016 | Novel |
| DB01340 | Q9BYF1 | 2.016 | Novel |
| DB01418 | P15559 | 2.006 | Novel |
| DB00251 | O15554 | 2.004 | Novel |
| DB00492 | Q9BYF1 | 2.003 | Novel |
| DB01133 | Q13332 | 2.001 | Novel |
| DB00118 | P48637 | 2 | Novel |
| DB00138 | O14920 | 2 | Novel |
| DB00168 | O60427 | 2 | Novel |
| DB00208 | P43119 | 2 | Novel |
| DB00530 | P04626 | 2 | Novel |
| DB00616 | Q9BYF1 | 2 | Novel |
| DB00758 | P43119 | 2 | Novel |
| DB01080 | Q9BYV1 | 2 | Novel |
| DB01087 | P15559 | 2 | Novel |
| DB01180 | Q9BYF1 | 2 | Novel |
| DB01263 | O15554 | 2 | Novel |
| DB01299 | P00374 | 2 | Novel |
| DB01369 | P60725 | 2 | Novel |
| DB01599 | Q09428 | 2 | Novel |
| DB04786 | P23219 | 2 | Novel |
| DB04878 | P10253 | 2 | Novel |
| DB05260 | P38435 | 2 | Novel |
| DB06155 | P34972 | 2 | Novel |
| DB06209 | P43119 | 2 | Novel |
| DB06228 | P01008 | 2 | Novel |
| DB06605 | P01008 | 2 | Novel |
| DB08604 | Q08129 | 2 | Novel |
| DB08816 | P43119 | 2 | Novel |
| DB08864 | Q9H4B7 | 2 | Novel |
| DB08933 | O15554 | 2 | Novel |
| DB00464 | P00734 | 2 | Exist |
| DB00583 | O95477 | 2 | Exist |
| DB00609 | P0A5Y6 | 2 | Exist |
| DB00786 | P14780 | 2 | Exist |
| DB00951 | P0A5Y6 | 2 | Exist |
| DB01022 | P00734 | 2 | Exist |
| DB01025 | P35367 | 2 | Exist |
| DB01074 | Q8N8R3 | 2 | Exist |
| DB01197 | P14780 | 2 | Exist |
| DB01259 | P04626 | 2 | Exist |
| DB01553 | P18505 | 1.972 | Novel |
| DB00879 | Q72547 | 1.964 | Exist |
| DB00701 | Q72874 | 1.959 | Exist |
| DB00634 | P0AC13 | 1.951 | Exist |
| DB00736 | P20648 | 1.951 | Exist |
| DB00778 | P61177 | 1.936 | Novel |
| DB00249 | P04818 | 1.921 | Novel |
| DB08905 | P10275 | 1.88 | Novel |
| DB00678 | P30556 | 1.828 | Exist |
| DB01319 | Q72874 | 1.811 | Exist |
| DB01129 | P20648 | 1.785 | Exist |
| DB00148 | P12277 | 1.761 | Exist |
| DB01264 | Q72874 | 1.757 | Exist |
| DB00194 | P03176 | 1.741 | Novel |
| DB00632 | P03200 | 1.732 | Exist |
| DB08819 | P43088 | 1.724 | Exist |
| DB01190 | P0A7J6 | 1.715 | Exist |
| DB01627 | P0A7J6 | 1.715 | Exist |
| DB00535 | P0AD68 | 1.711 | Novel |
| DB00287 | P43088 | 1.71 | Exist |
| DB00282 | P14324 | 1.704 | Exist |
| DB00894 | P10275 | 1.699 | Novel |
| DB00627 | Q8TDS4 | 1.689 | Exist |
| DB00641 | P04035 | 1.684 | Exist |
| DB00399 | P14324 | 1.666 | Exist |
| DB00213 | P20648 | 1.654 | Exist |
| DB00796 | P30556 | 1.647 | Exist |
| DB00884 | P14324 | 1.625 | Exist |
| DB01342 | P30556 | 1.612 | Exist |
| DB00448 | P20648 | 1.596 | Exist |
| DB00654 | P43088 | 1.585 | Exist |
| DB00259 | P0AC13 | 1.572 | Exist |
| DB01611 | P01375 | 1.55 | Exist |
| DB04845 | P68371 | 1.546 | Novel |
| DB00275 | P30556 | 1.522 | Exist |
| DB00928 | P26358 | 1.522 | Exist |
| DB01262 | P26358 | 1.522 | Exist |
| DB00190 | P08913 | 1.521 | Novel |
| DB02187 | P03372 | 1.498 | Novel |
| DB00495 | Q05486 | 1.482 | Novel |
| DB06729 | P0AC13 | 1.476 | Exist |
| DB01095 | P04035 | 1.461 | Exist |
| DB00357 | P43681 | 1.422 | Novel |
| DB00931 | P0A7X3 | 1.416 | Novel |
| DB01361 | P60725 | 1.406 | Novel |
| DB00158 | P04818 | 1.404 | Novel |
| DB08860 | P04035 | 1.387 | Exist |
| DB01326 | Q75Y35 | 1.357 | Novel |
| DB00955 | P0A7S3 | 1.352 | Exist |
| DB00990 | P10275 | 1.351 | Novel |
| DB00177 | P30556 | 1.351 | Exist |
| DB01091 | Q14534 | 1.344 | Exist |
| DB00478 | Q8TCU5 | 1.343 | Novel |
| DB01349 | P30556 | 1.343 | Exist |
| DB00649 | Q05486 | 1.324 | Novel |
| DB06715 | Q9UP95 | 1.305 | Novel |
| DB00735 | Q14534 | 1.29 | Exist |
| DB08981 | P23219 | 1.285 | Novel |
| DB00362 | A2QLK4 | 1.281 | Exist |
| DB00798 | P0A7S3 | 1.274 | Exist |
| DB00727 | P20594 | 1.26 | Novel |
| DB01613 | P16066 | 1.258 | Exist |
| DB01029 | P30556 | 1.256 | Exist |
| DB01141 | A2QLK4 | 1.238 | Exist |
| DB08796 | P14416 | 1.232 | Novel |
| DB00857 | Q14534 | 1.222 | Exist |
| DB00923 | Q70KI2 | 1.22 | Exist |
| DB00147 | O00764 | 1.219 | Exist |
| DB00165 | O00764 | 1.219 | Exist |
| DB06292 | P13866 | 1.212 | Novel |
| DB08907 | P31639 | 1.21 | Exist |
| DB00710 | P14324 | 1.196 | Exist |
| DB00173 | Q13085 | 1.194 | Novel |
| DB00121 | Q13085 | 1.194 | Exist |
| DB01098 | P04035 | 1.193 | Exist |
| DB00639 | P10613 | 1.176 | Novel |
| DB01606 | P62594 | 1.163 | Exist |
| DB04876 | P27487 | 1.16 | Exist |
| DB06335 | P27487 | 1.16 | Exist |
| DB03255 | P08588 | 1.153 | Novel |
| DB06150 | P0AC13 | 1.148 | Novel |
| DB00224 | Q72874 | 1.135 | Exist |
| DB01076 | P04035 | 1.133 | Exist |
| DB00175 | P04035 | 1.126 | Exist |
| DB00900 | P09884 | 1.109 | Novel |
| DB01232 | Q72874 | 1.106 | Exist |
| DB08967 | P35367 | 1.1 | Novel |
| DB00325 | P20594 | 1.098 | Novel |
| DB00883 | P20594 | 1.098 | Novel |
| DB01612 | P20594 | 1.098 | Novel |
| DB08941 | P07550 | 1.098 | Novel |
| DB01207 | P23219 | 1.094 | Novel |
| DB01586 | Q04828 | 1.083 | Novel |
| DB08822 | P30556 | 1.078 | Exist |
| DB00987 | P26358 | 1.075 | Novel |
| DB00220 | Q72874 | 1.067 | Novel |
| DB01201 | P0A8V2 | 1.067 | Novel |
| DB01601 | Q72874 | 1.063 | Exist |
| DB05812 | P06401 | 1.061 | Novel |
| DB01006 | P10613 | 1.06 | Novel |
| DB00551 | P08254 | 1.053 | Novel |
| DB00156 | P26639 | 1.052 | Exist |
| DB04877 | O95342 | 1.048 | Novel |
| DB00141 | P15291 | 1.046 | Exist |
| DB00520 | A2QLK4 | 1.045 | Exist |
| DB01032 | Q4U2R8 | 1.045 | Exist |
| DB06203 | P27487 | 1.044 | Exist |
| DB08882 | P27487 | 1.044 | Exist |
| DB06777 | P52895 | 1.005 | Novel |
| DB00117 | P08243 | 1.001 | Novel |
| DB01048 | P00491 | 1.001 | Novel |
| DB08865 | P00519 | 1.001 | Novel |
| DB00817 | P0AFI2 | 1.001 | Exist |
| DB01099 | P12461 | 1.001 | Exist |
| DB01145 | Q27738 | 1.001 | Exist |
| DB00181 | P80404 | 1 | Novel |
| DB00300 | P00491 | 1 | Novel |
| DB00808 | P62158 | 1 | Novel |
| DB01250 | Q00653 | 1 | Novel |
| DB06795 | P35498 | 1 | Novel |
| DB00127 | P11926 | 1 | Exist |
| DB00130 | P00492 | 1 | Exist |
| DB00140 | Q969G6 | 1 | Exist |
| DB00150 | P23381 | 1 | Exist |
| DB00152 | Q9H3S4 | 1 | Exist |
| DB00166 | Q9Y234 | 1 | Exist |
| DB00183 | P32239 | 1 | Exist |
| DB00198 | P06818 | 1 | Exist |
| DB00212 | P00797 | 1 | Exist |
| DB00238 | P00491 | 1 | Exist |
| DB00250 | P0C0X1 | 1 | Exist |
| DB00260 | P0A6J8 | 1 | Exist |
| DB00262 | P29475 | 1 | Exist |
| DB00278 | P00734 | 1 | Exist |
| DB00290 | P49916 | 1 | Exist |
| DB00302 | P00747 | 1 | Exist |
| DB00323 | P17707 | 1 | Exist |
| DB00330 | P0A560 | 1 | Exist |
| DB00336 | P61889 | 1 | Exist |
| DB00348 | P32754 | 1 | Exist |
| DB00364 | P08620 | 1 | Exist |
| DB00369 | P04293 | 1 | Exist |
| DB00389 | P10828 | 1 | Exist |
| DB00400 | P10875 | 1 | Exist |
| DB00403 | P32238 | 1 | Exist |
| DB00410 | P41972 | 1 | Exist |
| DB00419 | Q16739 | 1 | Exist |
| DB00428 | P11168 | 1 | Exist |
| DB00435 | P33402 | 1 | Exist |
| DB00437 | P29475 | 1 | Exist |
| DB00446 | P08174 | 1 | Exist |
| DB00494 | P17707 | 1 | Exist |
| DB00503 | Q72874 | 1 | Exist |
| DB00507 | P94692 | 1 | Exist |
| DB00512 | P96558 | 1 | Exist |
| DB00513 | P23219 | 1 | Exist |
| DB00525 | Q92206 | 1 | Exist |
| DB00536 | P12277 | 1 | Exist |
| DB00549 | Q9NS75 | 1 | Exist |
| DB00550 | P10828 | 1 | Exist |
| DB00552 | O76074 | 1 | Exist |
| DB00558 | P06818 | 1 | Exist |
| DB00581 | P06864 | 1 | Exist |
| DB00587 | Q9NS75 | 1 | Exist |
| DB00600 | P14679 | 1 | Exist |
| DB00613 | P50135 | 1 | Exist |
| DB00625 | P00491 | 1 | Exist |
| DB00626 | P01023 | 1 | Exist |
| DB00638 | Q9F0I5 | 1 | Exist |
| DB00648 | P10109 | 1 | Exist |
| DB00673 | P20309 | 1 | Exist |
| DB00686 | P35916 | 1 | Exist |
| DB00693 | P06310 | 1 | Exist |
| DB00698 | P0A7R5 | 1 | Exist |
| DB00705 | P00491 | 1 | Exist |
| DB00707 | P01130 | 1 | Exist |
| DB00716 | Q9NS75 | 1 | Exist |
| DB00718 | P24024 | 1 | Exist |
| DB00724 | Q9NYK1 | 1 | Exist |
| DB00730 | P00363 | 1 | Exist |
| DB00738 | O14717 | 1 | Exist |
| DB00756 | Q9BZW2 | 1 | Exist |
| DB00763 | P10828 | 1 | Exist |
| DB00766 | P00807 | 1 | Exist |
| DB00775 | P08514 | 1 | Exist |
| DB00789 | P35367 | 1 | Exist |
| DB00822 | P21728 | 1 | Exist |
| DB00828 | P0A749 | 1 | Exist |
| DB00845 | Q5L2G3 | 1 | Exist |
| DB00847 | P61278 | 1 | Exist |
| DB00851 | P35367 | 1 | Exist |
| DB00855 | P13716 | 1 | Exist |
| DB00876 | P30556 | 1 | Exist |
| DB00895 | P12319 | 1 | Exist |
| DB00906 | P30531 | 1 | Exist |
| DB00916 | P29166 | 1 | Exist |
| DB00919 | P0A7X3 | 1 | Exist |
| DB00932 | Q72874 | 1 | Exist |
| DB00973 | P35610 | 1 | Exist |
| DB00992 | P01130 | 1 | Exist |
| DB00993 | P00492 | 1 | Exist |
| DB01011 | P10109 | 1 | Exist |
| DB01012 | P41180 | 1 | Exist |
| DB01020 | P33402 | 1 | Exist |
| DB01034 | P0A6R0 | 1 | Exist |
| DB01046 | P51788 | 1 | Exist |
| DB01051 | P43700 | 1 | Exist |
| DB01072 | Q72874 | 1 | Exist |
| DB01082 | P0A7X3 | 1 | Exist |
| DB01083 | P0A6R0 | 1 | Exist |
| DB01094 | P35610 | 1 | Exist |
| DB01097 | P10275 | 1 | Exist |
| DB01103 | P23219 | 1 | Exist |
| DB01117 | Q02127 | 1 | Exist |
| DB01123 | P00734 | 1 | Exist |
| DB01138 | P00519 | 1 | Exist |
| DB01143 | P49902 | 1 | Exist |
| DB01206 | Q9H169 | 1 | Exist |
| DB01217 | P10275 | 1 | Exist |
| DB01219 | P30542 | 1 | Exist |
| DB01256 | Q9A1X4 | 1 | Exist |
| DB01261 | P04035 | 1 | Exist |
| DB01282 | P30559 | 1 | Exist |
| DB01321 | P44345 | 1 | Exist |
| DB01347 | P30556 | 1 | Exist |
| DB01422 | Q99707 | 1 | Exist |
| DB01597 | P16444 | 1 | Exist |
| DB01764 | P50870 | 1 | Exist |
| DB02703 | P00484 | 1 | Exist |
| DB02959 | Q9RVD6 | 1 | Exist |
| DB04835 | P51681 | 1 | Exist |
| DB04865 | P39023 | 1 | Exist |
| DB04898 | P00734 | 1 | Exist |
| DB05265 | P18314 | 1 | Exist |
| DB05389 | Q7L5Y9 | 1 | Exist |
| DB05521 | B0B3C9 | 1 | Exist |
| DB06196 | P35610 | 1 | Exist |
| DB06210 | P40238 | 1 | Exist |
| DB06290 | Q91RS4 | 1 | Exist |
| DB06439 | P06858 | 1 | Exist |
| DB06689 | P00748 | 1 | Exist |
| DB06695 | P00734 | 1 | Exist |
| DB06775 | P31327 | 1 | Exist |
| DB06809 | P0A7S3 | 1 | Exist |
| DB06817 | Q7ZJM1 | 1 | Exist |
| DB08820 | P23219 | 1 | Exist |
| DB08827 | P35610 | 1 | Exist |
| DB08828 | P04150 | 1 | Exist |
| DB08868 | Q9H228 | 1 | Exist |
| DB08873 | B0B3C9 | 1 | Exist |
| DB08874 | Q18BX5 | 1 | Exist |
| DB08877 | O60674 | 1 | Exist |
| DB08890 | P25092 | 1 | Exist |
| DB08894 | P19235 | 1 | Exist |
| DB08908 | Q14145 | 1 | Exist |
| DB08930 | Q7ZJM1 | 1 | Exist |
| DB06770 | P35348 | 0.82 | Novel |
| DB01501 | P35367 | 0.817 | Novel |
| DB08990 | P35367 | 0.796 | Novel |
| DB01610 | P04293 | 0.775 | Novel |
| DB01550 | P27338 | 0.699 | Novel |
| DB06718 | P10275 | 0.674 | Novel |
| DB00832 | P14867 | 0.644 | Novel |
| DB00954 | P0A7J6 | 0.627 | Novel |
| DB01428 | P35354 | 0.587 | Novel |
| DB09015 | P10275 | 0.586 | Novel |
| DB00874 | P08588 | 0.541 | Novel |
| DB08983 | P35367 | 0.541 | Novel |
| DB00859 | P54687 | 0.497 | Novel |
| DB00779 | P43700 | 0.427 | Novel |
| DB06717 | P25103 | 0.394 | Novel |
| DB09003 | P14416 | 0.39 | Novel |
| DB00556 | P47870 | 0.376 | Novel |
| DB08824 | P11229 | 0.348 | Novel |
| DB00352 | P12268 | 0.327 | Novel |
| DB00676 | P35367 | 0.321 | Novel |
| DB00345 | P35354 | 0.313 | Novel |
| DB00395 | P47870 | 0.311 | Novel |
| DB00442 | P09884 | 0.307 | Novel |
| DB08968 | P35367 | 0.296 | Novel |
| DB00815 | P04070 | 0.295 | Novel |
| DB08924 | P31645 | 0.294 | Novel |
| DB08803 | P08913 | 0.275 | Novel |
| DB08887 | O60427 | 0.27 | Novel |
| DB08992 | Q9Y5Y9 | 0.267 | Novel |
| DB01168 | P08588 | 0.264 | Novel |
| DB08797 | P35354 | 0.264 | Novel |
| DB08957 | P07550 | 0.256 | Novel |
| DB08847 | Q96C36 | 0.247 | Novel |
| DB00265 | Q14524 | 0.243 | Novel |
| DB01164 | Q9UP95 | 0.243 | Novel |
| DB08834 | P52895 | 0.24 | Novel |
| DB08994 | P35367 | 0.238 | Novel |
| DB01424 | P23219 | 0.235 | Novel |
| DB01042 | P07101 | 0.232 | Novel |
| DB00743 | P52209 | 0.194 | Novel |
| DB00856 | P08588 | 0.193 | Novel |
| DB08979 | P11229 | 0.183 | Novel |
| DB06402 | P96558 | 0.172 | Novel |
| DB08842 | O76082 | 0.172 | Novel |
| DB06811 | P04070 | 0.166 | Novel |
| DB06804 | Q14524 | 0.16 | Novel |
| DB08984 | P14416 | 0.16 | Novel |
| DB01344 | P0AC13 | 0.159 | Novel |
| DB00601 | P00742 | 0.149 | Novel |
| DB00911 | P29166 | 0.14 | Novel |
| DB09008 | Q8DR59 | 0.138 | Novel |
| DB06590 | P0AD68 | 0.135 | Novel |
| DB00566 | Q12797 | 0.134 | Novel |
| DB06768 | P24298 | 0.123 | Novel |
| DB06704 | P08913 | 0.12 | Novel |
| DB00258 | Q12797 | 0.11 | Novel |
| DB08974 | Q71U36 | 0.105 | Novel |
| DB04911 | P96558 | 0.095 | Novel |
| DB01243 | P50579 | 0.091 | Novel |
| DB06803 | B0Q840 | 0.089 | Novel |
| DB00614 | P0A7R5 | 0.08 | Novel |
| DB01609 | P30556 | 0.079 | Novel |
| DB00971 | O14732 | 0.071 | Novel |
| DB01377 | O14732 | 0.071 | Novel |
| DB08835 | P08263 | 0.071 | Novel |
| DB01482 | P35367 | 0.07 | Novel |
| DB01058 | P46098 | 0.068 | Novel |
| DB06708 | Q12809 | 0.065 | Novel |
| DB08987 | Q14524 | 0.065 | Novel |
| DB00291 | Q4U2R8 | 0.055 | Novel |
| DB01187 | Q9Y5Y9 | 0.055 | Novel |
| DB03166 | P34897 | 0.053 | Novel |
| DB00662 | P07550 | 0.051 | Novel |
| DB00781 | P01023 | 0.05 | Novel |
| DB00236 | P23219 | 0.044 | Novel |
| DB00742 | P15291 | 0.044 | Novel |
| DB00791 | P04818 | 0.044 | Novel |
| DB08792 | P10109 | 0.044 | Novel |
| DB08866 | P04150 | 0.044 | Novel |
| DB00803 | P01023 | 0.003 | Novel |
| DB00339 | P35354 | 0.001 | Novel |
| DB08913 | Q9H2X9 | 0.001 | Novel |
| DB08976 | P21728 | 0.001 | Novel |
| DB01111 | P01023 | ~ 0 | Novel |
| DB01390 | P48167 | ~ 0 | Novel |
| DB09009 | Q9Y5Y9 | ~ 0 | Novel |

Table S8. ‘Top 1’ predictions for the HGBI Dataset. ‘Exist’ refers to predictions of links that represent known DTIs in the network. Novel refers to new predictions that are not known DTIs in the network.

| **chemName (DrugBank)** | **protID (HGNC)** | **Score** | **Type** |
| --- | --- | --- | --- |
| amifostine | 3356 | 315 | Exist |
| temazepam | 3356 | 307 | Exist |
| acid | 3356 | 307 | Exist |
| bupropion | 3356 | 307 | Exist |
| terfenadine | 3356 | 298 | Exist |
| retapamulin | 3356 | 256.403 | Exist |
| isoproterenol | 148 | 238 | Exist |
| phenacemide | 150 | 237.062 | Exist |
| methyprylon | 150 | 230.314 | Exist |
| mycophenolate | 3356 | 204.706 | Exist |
| rescinnamine | 3356 | 204.706 | Exist |
| aminosalicylic | 1813 | 200.253 | Exist |
| cefixime | 1813 | 196 | Exist |
| phenytoin | 1813 | 194 | Exist |
| ethopropazine | 1813 | 194 | Exist |
| acid | 1128 | 171.157 | Exist |
| divalproex | 1128 | 160.157 | Exist |
| trifluridine | 1128 | 158.074 | Exist |
| fludarabine | 1128 | 148.074 | Exist |
| auranofin | 1128 | 142.3 | Exist |
| trazodone | 148 | 137.675 | Exist |
| diclofenac | 1128 | 116 | Exist |
| chlorpropamide | 1128 | 116 | Exist |
| clodronate | 1128 | 106 | Exist |
| trimethadione | 148 | 105.616 | Exist |
| l-proline | 2555 | 100.204 | Exist |
| levothyroxine | 150 | 99.186 | Novel |
| tacrine | 2555 | 98.833 | Exist |
| dipivefrin | 2555 | 95.833 | Exist |
| guanabenz | 148 | 93.937 | Exist |
| rosiglitazone | 1132 | 93.379 | Novel |
| propiomazine | 2557 | 92.512 | Novel |
| neomycin | 2555 | 92.159 | Exist |
| aztreonam | 1128 | 91.621 | Exist |
| nortriptyline | 1128 | 89.793 | Exist |
| eplerenone | 1129 | 89.63 | Exist |
| adenosine | 150 | 84.073 | Novel |
| etodolac | 1813 | 84 | Novel |
| buprenorphine | 3356 | 82 | Novel |
| sertaconazole | 3356 | 82 | Novel |
| lenalidomide | 3356 | 78.7 | Novel |
| cefotetan | 148 | 76.951 | Novel |
| grepafloxacin | 1128 | 74.621 | Exist |
| ceruletide | 1129 | 72.798 | Exist |
| diflunisal | 147 | 71 | Novel |
| oxamniquine | 1129 | 71 | Novel |
| nalbuphine | 1131 | 65 | Novel |
| sulfisoxazole | 1132 | 64.072 | Novel |
| procyclidine | 150 | 64 | Novel |
| succinylcholine | 150 | 63.045 | Novel |
| methohexital | 3358 | 63.002 | Novel |
| nefazodone | 1132 | 60.29 | Novel |
| rivastigmine | 1132 | 60.073 | Novel |
| edrophonium | 1132 | 60.072 | Novel |
| tranexamic | 3350 | 58.582 | Novel |
| fluvastatin | 2554 | 57.867 | Exist |
| dimeglumine | 2555 | 56.91 | Exist |
| colestipol | 147 | 56.772 | Novel |
| quetiapine | 2555 | 56.714 | Exist |
| sulfate | 2555 | 56.713 | Exist |
| primidone | 148 | 55.838 | Exist |
| dinoprost | 148 | 52.929 | Novel |
| emedastine | 148 | 52.611 | Exist |
| piroxicam | 148 | 52.408 | Exist |
| lindane | 148 | 52.255 | Exist |
| masoprocol | 1128 | 51.27 | Novel |
| isoflurane | 1131 | 50.001 | Novel |
| dihydrotachysterol | 1131 | 50 | Novel |
| imipramine | 148 | 49 | Novel |
| benazepril | 148 | 49 | Novel |
| mecamylamine | 148 | 49 | Novel |
| yohimbine | 150 | 49 | Novel |
| cefoperazone | 150 | 47.433 | Novel |
| doxylamine | 148 | 46.021 | Exist |
| nalidixic | 3356 | 45.627 | Novel |
| proparacaine | 147 | 45.008 | Novel |
| nitroprusside | 148 | 44.419 | Exist |
| pregabalin | 1129 | 44.155 | Novel |
| hyoscyamine | 147 | 44 | Novel |
| benzthiazide | 150 | 44 | Novel |
| sulfametopyrazine | 148 | 44 | Exist |
| ritodrine | 1812 | 43.989 | Novel |
| minoxidil | 2555 | 43.833 | Exist |
| amitriptyline | 1128 | 43.802 | Novel |
| cinalukast | 3356 | 43.541 | Novel |
| bupivacaine | 150 | 43 | Novel |
| methadone | 1129 | 43 | Novel |
| aminohippurate | 1129 | 43 | Novel |
| alendronate | 150 | 43 | Novel |
| pemirolast | 150 | 43 | Novel |
| exemestane | 3356 | 42.256 | Novel |
| dobutamine | 3352 | 42.112 | Novel |
| flumethasone | 4988 | 41.343 | Exist |
| magnesium | 147 | 41 | Novel |
| ceftizoxime | 3356 | 41 | Novel |
| tenoxicam | 4988 | 40.999 | Exist |
| sodium | 3356 | 40 | Novel |
| netilmicin | 775 | 39.391 | Exist |
| ibutilide | 4988 | 39.355 | Exist |
| bumetanide | 3350 | 39 | Novel |
| toremifene | 150 | 38 | Novel |
| desipramine | 1129 | 38 | Novel |
| papaverine | 4986 | 37.95 | Novel |
| benzylpenicilloyl | 3352 | 37.113 | Novel |
| praziquantel | 147 | 37 | Novel |
| tetrahydrobiopterin | 775 | 35.995 | Exist |
| fenofibrate | 775 | 35.677 | Exist |
| dienestrol | 4986 | 35.649 | Novel |
| aminolevulinic | 3350 | 35.566 | Novel |
| fluconazole | 150 | 35.011 | Novel |
| mesoridazine | 3350 | 35 | Novel |
| terbinafine | 4985 | 34.339 | Exist |
| nicardipine | 781 | 34 | Novel |
| naproxen | 4988 | 33.779 | Exist |
| doxycycline | 775 | 33.746 | Exist |
| meprobamate | 775 | 32.935 | Exist |
| dexamethasone | 147 | 32.837 | Novel |
| valsartan | 6530 | 32.22 | Exist |
| furazolidone | 776 | 32.219 | Novel |
| mexiletine | 775 | 31.826 | Exist |
| cefotiam | 5742 | 31.7 | Exist |
| zileuton | 5742 | 31.124 | Exist |
| nitrofurantoin | 148 | 31 | Novel |
| guanethidine | 148 | 31 | Novel |
| tenofovir | 4985 | 30.466 | Exist |
| argatroban | 4985 | 30.456 | Exist |
| methoxyflurane | 6530 | 30.17 | Novel |
| phenprocoumon | 5742 | 30.124 | Exist |
| cephalexin | 1813 | 30 | Novel |
| tetracycline | 1129 | 29.884 | Novel |
| irbesartan | 6530 | 29.359 | Exist |
| ropinirole | 6530 | 29.282 | Exist |
| droperidol | 6530 | 29.231 | Exist |
| nisoldipine | 6530 | 29.176 | Exist |
| amodiaquine | 4985 | 28.542 | Novel |
| terbutaline | 6532 | 28.238 | Novel |
| vindesine | 5742 | 28.124 | Exist |
| fenoprofen | 4988 | 28.063 | Exist |
| acid | 6532 | 28.059 | Novel |
| chloride | 4985 | 27.207 | Exist |
| loperamide | 4985 | 27.207 | Exist |
| valproic | 4986 | 27.204 | Novel |
| chloroquine | 4985 | 27.153 | Exist |
| loteprednol | 5742 | 27.124 | Exist |
| hesperetin | 1128 | 27.003 | Novel |
| chloramphenicol | 6530 | 26.201 | Novel |
| oxaprozin | 153 | 26.128 | Exist |
| cinnarizine | 5742 | 25.124 | Exist |
| thalidomide | 154 | 25 | Novel |
| metharbital | 153 | 25 | Exist |
| suprofen | 5742 | 24.702 | Exist |
| pentamidine | 5742 | 24.379 | Exist |
| triazolam | 5742 | 24.355 | Exist |
| cycrimine | 5742 | 24.355 | Exist |
| ceftazidime | 5742 | 24.29 | Exist |
| fluoxetine | 5742 | 24.29 | Exist |
| nitric | 5742 | 24.251 | Exist |
| vincristine | 5742 | 24.21 | Exist |
| cyclopentolate | 5742 | 24.168 | Exist |
| meperidine | 5742 | 24.134 | Novel |
| azelastine | 5742 | 24.134 | Novel |
| ergonovine | 5742 | 24.127 | Exist |
| indomethacin | 5743 | 24.124 | Novel |
| cefalotin | 5742 | 24.124 | Novel |
| dapiprazole | 5742 | 24.124 | Exist |
| carteolol | 5742 | 24.124 | Exist |
| acetohydroxamic | 5742 | 24.124 | Exist |
| sumatriptan | 5742 | 24.124 | Exist |
| methazolamide | 5742 | 24.124 | Exist |
| irinotecan | 5742 | 24.124 | Exist |
| colistin | 5742 | 24.124 | Exist |
| cyclobenzaprine | 5742 | 24.124 | Exist |
| pseudoephedrine | 1812 | 24 | Novel |
| cefadroxil | 1812 | 24 | Novel |
| cabergoline | 154 | 23.879 | Exist |
| baclofen | 154 | 23.708 | Exist |
| diphenidol | 153 | 23.397 | Exist |
| salicyclic | 153 | 23.373 | Exist |
| proguanil | 153 | 23.304 | Exist |
| flurandrenolide | 760 | 23.297 | Exist |
| clonidine | 153 | 23.27 | Exist |
| tolbutamide | 153 | 23.2 | Exist |
| pivalate | 154 | 23.125 | Exist |
| ajmaline | 153 | 23.085 | Exist |
| bleomycin | 783 | 23.083 | Novel |
| chlorpheniramine | 153 | 23.073 | Exist |
| fosinopril | 153 | 23.07 | Exist |
| aciclovir | 1813 | 23 | Novel |
| thioguanine | 153 | 23 | Exist |
| dexfenfluramine | 153 | 23 | Exist |
| betazole | 6531 | 22.232 | Novel |
| maprotiline | 760 | 21.613 | Exist |
| cholecalciferol | 6530 | 20.313 | Novel |
| dicloxacillin | 6530 | 20 | Novel |
| labetalol | 148 | 20 | Novel |
| acetohexamide | 760 | 19.231 | Exist |
| benzquinamide | 760 | 19.191 | Exist |
| leflunomide | 1128 | 18.37 | Novel |
| tadalafil | 760 | 18.096 | Exist |
| clotrimazole | 759 | 17.096 | Novel |
| nifedipine | 760 | 17.096 | Exist |
| cycloserine | 134 | 17.037 | Exist |
| moxifloxacin | 760 | 16.631 | Exist |
| isoflurophate | 6530 | 16.239 | Novel |
| orlistat | 6530 | 16.191 | Novel |
| ribavirin | 760 | 16.096 | Exist |
| sertraline | 4988 | 15.537 | Novel |
| bisoprolol | 5142 | 14.057 | Exist |
| chloroprocaine | 781 | 14.051 | Novel |
| carboprost | 4985 | 13.134 | Novel |
| entacapone | 6258 | 12.453 | Exist |
| levocabastine | 5241 | 12.354 | Novel |
| lorazepam | 5141 | 12.348 | Novel |
| tretinoin | 134 | 12.074 | Exist |
| methacycline | 775 | 12 | Novel |
| alfuzosin | 5241 | 11.582 | Exist |
| miconazole | 2555 | 11.283 | Novel |
| doxepin | 135 | 11.059 | Novel |
| iodixanol | 2099 | 11.047 | Novel |
| cerulenin | 148 | 11 | Novel |
| lamivudine | 6256 | 10.576 | Novel |
| linezolid | 5142 | 10.354 | Novel |
| treprostinil | 5241 | 10.293 | Exist |
| brompheniramine | 2555 | 10.283 | Novel |
| betaxolol | 6258 | 10.27 | Exist |
| edetic | 1813 | 10.026 | Novel |
| succimer | 5241 | 9.46 | Exist |
| clobetasol | 134 | 9 | Novel |
| ifosfamide | 3815 | 9 | Exist |
| pentostatin | 2099 | 8.025 | Novel |
| atomoxetine | 5916 | 8 | Novel |
| prednisone | 2554 | 8 | Novel |
| aliskiren | 2099 | 8 | Exist |
| ethambutol | 2099 | 7.895 | Novel |
| estrogens | 5241 | 7.306 | Exist |
| paricalcitol | 2100 | 7.271 | Novel |
| risperidone | 2099 | 7.271 | Exist |
| quinidine | 5142 | 7.077 | Novel |
| clofazimine | 81033 | 7.063 | Novel |
| trihexyphenidyl | 1436 | 7 | Exist |
| l-carnitine | 3815 | 7 | Exist |
| sodium | 7846 | 6.633 | Novel |
| gadoversetamide | 7277 | 6.633 | Novel |
| secobarbital | 2908 | 6.545 | Novel |
| cisplatin | 2908 | 6.465 | Novel |
| mitotane | 5241 | 6.415 | Novel |
| l-aspartic | 135 | 6.345 | Novel |
| sulfapyridine | 5241 | 6.299 | Exist |
| griseofulvin | 5241 | 6.193 | Novel |
| enalapril | 2908 | 6.176 | Novel |
| valrubicin | 2159 | 6.016 | Novel |
| mercaptopurine | 462 | 6.016 | Exist |
| dichlorphenamide | 462 | 6.016 | Exist |
| oxiconazole | 5241 | 6 | Novel |
| loratadine | 5241 | 6 | Novel |
| clofibrate | 5241 | 6 | Novel |
| atovaquone | 5241 | 6 | Novel |
| bismuth | 134 | 6 | Novel |
| rimantadine | 5241 | 5.94 | Novel |
| galantamine | 5241 | 5.94 | Novel |
| tubocurarine | 134 | 5.801 | Novel |
| daunorubicin | 5241 | 5.59 | Novel |
| candicidin | 2908 | 5.347 | Exist |
| b | 3356 | 5.113 | Novel |
| mitomycin | 2908 | 5.106 | Novel |
| selegiline | 5141 | 5.077 | Novel |
| ethchlorvynol | 81033 | 5.065 | Novel |
| tipranavir | 7153 | 5.063 | Novel |
| quinacrine | 81033 | 5.063 | Novel |
| phenindione | 1128 | 5 | Novel |
| isosorbide | 3359 | 5 | Novel |
| trioxide | 1436 | 5 | Exist |
| darunavir | 134 | 4.878 | Novel |
| gamma-homolinolenic | 5916 | 4.753 | Novel |
| docetaxel | 10383 | 4.724 | Novel |
| pipecuronium | 7067 | 4.704 | Novel |
| amantadine | 5916 | 4.508 | Novel |
| eprosartan | 1128 | 4.486 | Novel |
| rosuvastatin | 7153 | 4.472 | Novel |
| carmustine | 7067 | 4.464 | Exist |
| famciclovir | 7067 | 4.464 | Exist |
| mezlocillin | 6833 | 4.445 | Exist |
| levorphanol | 5733 | 4.382 | Exist |
| fluticasone | 5241 | 4.375 | Novel |
| ethosuximide | 5422 | 4.369 | Exist |
| lovastatin | 5422 | 4.297 | Exist |
| ticlopidine | 6833 | 4.287 | Exist |
| tacrolimus | 5731 | 4.262 | Novel |
| metoprolol | 6331 | 4.23 | Exist |
| fluocinonide | 3767 | 4.14 | Novel |
| butabarbital | 5241 | 4.134 | Novel |
| chlorpromazine | 1128 | 4.126 | Novel |
| framycetin | 116443 | 4.052 | Novel |
| hydrochlorothiazide | 5422 | 4.044 | Exist |
| acid | 5742 | 4 | Novel |
| sulpiride | 5742 | 4 | Novel |
| epirubicin | 5742 | 4 | Novel |
| pentosan | 5742 | 4 | Novel |
| methylphenobarbital | 5742 | 4 | Novel |
| remifentanil | 5742 | 4 | Novel |
| felbamate | 5742 | 4 | Novel |
| azathioprine | 5742 | 4 | Novel |
| clozapine | 26 | 4 | Exist |
| zanamivir | 6337 | 4 | Exist |
| promazine | 7155 | 3.755 | Novel |
| latanoprost | 7153 | 3.689 | Exist |
| raloxifene | 7067 | 3.642 | Novel |
| pipobroman | 6335 | 3.55 | Novel |
| bethanidine | 2561 | 3.548 | Novel |
| etonogestrel | 2555 | 3.545 | Novel |
| mivacurium | 2555 | 3.545 | Novel |
| marimastat | 2561 | 3.53 | Novel |
| zoledronate | 1813 | 3.496 | Novel |
| amdinocillin | 1813 | 3.496 | Novel |
| alfentanil | 2908 | 3.447 | Novel |
| trimethaphan | 6323 | 3.335 | Novel |
| disopyramide | 6331 | 3.297 | Novel |
| cefmenoxime | 278 | 3.264 | Novel |
| bromodiphenhydramine | 4128 | 3.26 | Novel |
| oxazepam | 5732 | 3.136 | Novel |
| mefloquine | 6336 | 3.107 | Novel |
| desloratadine | 6336 | 3.107 | Novel |
| l-phenylalanine | 43 | 3.051 | Exist |
| vinorelbine | 43 | 3.051 | Exist |
| levosimendan | 43 | 3.051 | Exist |
| flunisolide | 5422 | 3.027 | Novel |
| isotretinoin | 776 | 3.019 | Novel |
| testolactone | 2908 | 3.014 | Novel |
| memantine | 3758 | 3.007 | Novel |
| bortezomib | 5141 | 3 | Novel |
| penicillin | 5422 | 3 | Novel |
| montelukast | 2099 | 3 | Novel |
| triamcinolone | 3359 | 3 | Novel |
| olopatadine | 5141 | 3 | Novel |
| butenafine | 5742 | 3 | Novel |
| flavoxate | 596 | 2.979 | Exist |
| chloride | 596 | 2.979 | Exist |
| bepridil | 2908 | 2.839 | Novel |
| diflorasone | 2555 | 2.809 | Novel |
| malathion | 134 | 2.665 | Novel |
| sucralfate | 7153 | 2.663 | Novel |
| pheniramine | 5241 | 2.644 | Novel |
| chlorambucil | 203068 | 2.641 | Novel |
| ciclesonide | 2099 | 2.616 | Novel |
| gliquidone | 5742 | 2.53 | Novel |
| nedocromil | 7150 | 2.528 | Exist |
| mepivacaine | 7150 | 2.528 | Exist |
| mitiglinide | 5742 | 2.523 | Novel |
| carbachol | 1813 | 2.394 | Novel |
| atropine | 148 | 2.368 | Novel |
| clocortolone | 1813 | 2.361 | Novel |
| mustard | 2555 | 2.353 | Novel |
| magnesium | 1813 | 2.332 | Novel |
| dactinomycin | 1813 | 2.296 | Novel |
| cefaclor | 2908 | 2.259 | Novel |
| amyl | 776 | 2.253 | Novel |
| levodopa | 6531 | 2.252 | Novel |
| tripelennamine | 1813 | 2.22 | Novel |
| mometasone | 153 | 2.183 | Novel |
| fentanyl | 153 | 2.183 | Novel |
| cefonicid | 2561 | 2.155 | Novel |
| thiothixene | 596 | 2.113 | Novel |
| ticarcillin | 148 | 2.107 | Novel |
| pantoprazole | 3156 | 2.091 | Exist |
| ivermectin | 3066 | 2.091 | Exist |
| amcinonide | 2555 | 2.089 | Novel |
| vitamin | 3156 | 2.045 | Novel |
| ciclopirox | 3351 | 2.043 | Novel |
| cimetidine | 2099 | 2 | Novel |
| misoprostol | 240 | 2 | Novel |
| diethylpropion | 759 | 2 | Novel |
| amiodarone | 59272 | 2 | Novel |
| proflavine | 775 | 2 | Novel |
| choline | 3757 | 2 | Exist |
| methylergonovine | 5739 | 2 | Exist |
| iodide | 471 | 2 | Exist |
| bentoquatam | 6715 | 2 | Exist |
| medroxyprogesterone | 1719 | 2 | Exist |
| pentazocine | 1636 | 2 | Exist |
| losartan | 1636 | 2 | Exist |
| chloride | 6716 | 2 | Exist |
| bretylium | 5467 | 2 | Exist |
| l-alanine | 5422 | 1.996 | Novel |
| metixene | 203068 | 1.986 | Novel |
| mirtazapine | 1813 | 1.97 | Novel |
| doxorubicin | 1813 | 1.956 | Novel |
| flutamide | 776 | 1.93 | Novel |
| tamsulosin | 6531 | 1.918 | Novel |
| estropipate | 5241 | 1.892 | Novel |
| entecavir | 3757 | 1.882 | Novel |
| candesartan | 10280 | 1.867 | Novel |
| duloxetine | 10280 | 1.863 | Novel |
| prilocaine | 2561 | 1.856 | Novel |
| nelarabine | 5241 | 1.825 | Novel |
| sulfacytine | 5241 | 1.781 | Novel |
| pentobarbital | 1128 | 1.728 | Novel |
| fluocinolone | 2561 | 1.718 | Novel |
| paclitaxel | 2099 | 1.713 | Novel |
| lisdexamfetamine | 5742 | 1.69 | Novel |
| forasartan | 2561 | 1.625 | Novel |
| salsalate | 3065 | 1.624 | Exist |
| propionate | 2908 | 1.605 | Novel |
| doxacurium | 2561 | 1.585 | Novel |
| megestrol | 1813 | 1.556 | Novel |
| enoxaparin | 2555 | 1.506 | Novel |
| testosterone | 2908 | 1.465 | Novel |
| mephentermine | 1813 | 1.452 | Novel |
| candoxatril | 2099 | 1.424 | Novel |
| rizatriptan | 759 | 1.419 | Novel |
| thiethylperazine | 2908 | 1.408 | Novel |
| oxide | 1813 | 1.408 | Novel |
| mifepristone | 2561 | 1.385 | Novel |
| dextromethorphan | 2561 | 1.36 | Novel |
| aminoglutethimide | 5241 | 1.357 | Novel |
| tasosartan | 2561 | 1.336 | Novel |
| acid | 6583 | 1.323 | Exist |
| phenylpropanolamine | 2555 | 1.32 | Novel |
| bendroflumethiazide | 2555 | 1.318 | Novel |
| diphemanil | 1813 | 1.295 | Novel |
| nadh | 18 | 1.285 | Novel |
| polylysine | 6336 | 1.268 | Novel |
| adefovir | 2908 | 1.267 | Novel |
| liothyronine | 6336 | 1.263 | Novel |
| acid | 6336 | 1.263 | Novel |
| dyphylline | 2561 | 1.262 | Novel |
| l-lysine | 5352 | 1.229 | Exist |
| desflurane | 153 | 1.221 | Novel |
| trospium | 2908 | 1.209 | Novel |
| vinblastine | 2938 | 1.207 | Novel |
| menthol | 57468 | 1.193 | Exist |
| thiamine | 2875 | 1.168 | Exist |
| alosetron | 6531 | 1.16 | Novel |
| terazosin | 116443 | 1.158 | Novel |
| erythrityl | 116443 | 1.158 | Novel |
| azithromycin | 153 | 1.151 | Novel |
| ciprofloxacin | 462 | 1.149 | Novel |
| hydroxyurea | 36 | 1.133 | Novel |
| oxyphenonium | 5742 | 1.128 | Novel |
| lomefloxacin | 3352 | 1.123 | Novel |
| uracil | 8973 | 1.118 | Novel |
| celecoxib | 36 | 1.102 | Novel |
| tolazamide | 6558 | 1.101 | Novel |
| quinethazone | 2561 | 1.09 | Novel |
| diltiazem | 2246 | 1.084 | Exist |
| propoxyphene | 1950 | 1.084 | Exist |
| morphine | 36 | 1.078 | Exist |
| caffeine | 6530 | 1.07 | Novel |
| spirapril | 2561 | 1.064 | Novel |
| encainide | 3612 | 1.053 | Exist |
| mesylate | 3781 | 1.037 | Novel |
| l-histidine | 5422 | 1.028 | Novel |
| prednisolone | 1129 | 1.022 | Novel |
| roxithromycin | 6530 | 1.021 | Novel |
| tetranitrate | 3612 | 1.012 | Novel |
| pargyline | 2908 | 1.004 | Novel |
| vancomycin | 1812 | 1 | Novel |
| methimazole | 5624 | 1 | Novel |
| cyanocobalamin | 4548 | 1 | Exist |
| l-methionine | 645 | 1 | Exist |
| l-citrulline | 501 | 1 | Exist |
| aspartame | 18 | 1 | Exist |
| l-asparagine | 5689 | 1 | Exist |
| butalbital | 1595 | 1 | Exist |
| topiramate | 3978 | 1 | Exist |
| acetaminophen | 2280 | 1 | Exist |
| nitrofurazone | 1583 | 1 | Exist |
| palonosetron | 523 | 1 | Exist |
| allopurinol | 5624 | 1 | Exist |
| norgestrel | 217 | 1 | Exist |
| oxaliplatin | 1909 | 1 | Exist |
| doxazosin | 4837 | 1 | Exist |
| piperazine | 523 | 1 | Exist |
| nilutamide | 2209 | 1 | Exist |
| aprepitant | 240 | 1 | Exist |
| moricizine | 51284 | 1 | Exist |
| nitroglycerin | 3674 | 1 | Exist |
| dolasetron | 476 | 1 | Exist |
| alprostadil | 217 | 1 | Exist |
| haloprogin | 5226 | 1 | Exist |
| pindolol | 185 | 1 | Exist |
| selenium | 5465 | 1 | Exist |
| levobupivacaine | 196 | 1 | Exist |
| busulfan | 2194 | 1 | Exist |
| mimosine | 523 | 1 | Exist |
| melatonin | 251 | 1 | Exist |
| orphenadrine | 1956 | 1 | Exist |
| domperidone | 1621 | 1 | Exist |
| brinzolamide | 2263 | 1 | Exist |
| theobromine | 2099 | 0.986 | Novel |
| almotriptan | 134 | 0.977 | Novel |
| acid | 2908 | 0.977 | Novel |
| methoxamine | 5733 | 0.958 | Novel |
| amphotericin | 1128 | 0.946 | Novel |
| bicarbonate | 1813 | 0.941 | Novel |
| clomipramine | 5241 | 0.937 | Novel |
| tropicamide | 148 | 0.936 | Novel |
| ardeparin | 5733 | 0.917 | Novel |
| pyruvic | 6531 | 0.909 | Novel |
| streptomycin | 5733 | 0.898 | Novel |
| methdilazine | 1812 | 0.888 | Novel |
| capreomycin | 154 | 0.887 | Novel |
| pentoxifylline | 2280 | 0.875 | Novel |
| mefenamic | 6833 | 0.855 | Novel |
| fluoxymesterone | 5422 | 0.846 | Novel |
| quinapril | 2099 | 0.837 | Novel |
| trifluoperazine | 5241 | 0.836 | Novel |
| dinitrate | 1131 | 0.813 | Novel |
| naltrexone | 6336 | 0.81 | Novel |
| flurazepam | 3269 | 0.805 | Novel |
| adenosine | 1812 | 0.796 | Novel |
| cevimeline | 4548 | 0.794 | Novel |
| l-leucine | 5422 | 0.788 | Novel |
| quinupristin | 2939 | 0.779 | Novel |
| lopinavir | 6530 | 0.764 | Novel |
| tigecycline | 2908 | 0.762 | Novel |
| methotrexate | 2555 | 0.728 | Novel |
| dinoprostone | 5241 | 0.728 | Novel |
| oxide | 1128 | 0.713 | Novel |
| ethanol | 150 | 0.71 | Novel |
| nimodipine | 6833 | 0.707 | Novel |
| demeclocycline | 116443 | 0.707 | Novel |
| l-ornithine | 2875 | 0.694 | Novel |
| sodium | 1813 | 0.681 | Novel |
| prazepam | 5148 | 0.675 | Novel |
| ceforanide | 5241 | 0.667 | Novel |
| vidarabine | 1129 | 0.664 | Novel |
| dipivoxil | 1812 | 0.661 | Novel |
| dextrothyroxine | 2099 | 0.656 | Novel |
| ketazolam | 148 | 0.645 | Novel |
| fluvoxamine | 1812 | 0.643 | Novel |
| estradiol | 2908 | 0.636 | Novel |
| chlorotrianisene | 2099 | 0.631 | Novel |
| pranlukast | 8856 | 0.631 | Novel |
| amprenavir | 1128 | 0.628 | Novel |
| c | 36 | 0.613 | Novel |
| ketotifen | 6240 | 0.613 | Novel |
| l-glutamic | 36 | 0.607 | Novel |
| adenine | 154 | 0.602 | Novel |
| fosamprenavir | 1583 | 0.599 | Novel |
| lamotrigine | 2908 | 0.574 | Novel |
| indapamide | 154 | 0.573 | Novel |
| acid | 5742 | 0.565 | Novel |
| atorvastatin | 2555 | 0.564 | Novel |
| testosterone | 5241 | 0.563 | Novel |
| salbutamol | 6530 | 0.556 | Novel |
| methantheline | 3781 | 0.548 | Novel |
| rosoxacin | 2280 | 0.531 | Novel |
| solifenacin | 2280 | 0.526 | Novel |
| nateglinide | 6531 | 0.522 | Novel |
| spermine | 36 | 0.515 | Novel |
| ipratropium | 3351 | 0.508 | Novel |
| nadolol | 760 | 0.507 | Novel |
| trimipramine | 759 | 0.504 | Novel |
| arsenic | 3351 | 0.503 | Novel |
| sulfonate | 2280 | 0.502 | Novel |
| ergocalciferol | 18 | 0.496 | Novel |
| calcitriol | 36 | 0.483 | Novel |
| amlexanox | 153 | 0.476 | Novel |
| nandrolone | 6336 | 0.47 | Novel |
| ketoconazole | 2939 | 0.466 | Novel |
| thiamylal | 513 | 0.465 | Novel |
| methyclothiazide | 3351 | 0.459 | Novel |
| polystyrene | 2561 | 0.445 | Novel |
| venlafaxine | 781 | 0.444 | Novel |
| ethinyl | 2908 | 0.443 | Novel |
| emtricitabine | 5742 | 0.442 | Novel |
| metolazone | 3269 | 0.441 | Novel |
| aprindine | 203068 | 0.429 | Novel |
| suramin | 154 | 0.426 | Novel |
| sirolimus | 6240 | 0.424 | Novel |
| l-isoleucine | 2908 | 0.423 | Novel |
| gatifloxacin | 2555 | 0.421 | Novel |
| valganciclovir | 2099 | 0.414 | Novel |
| ritonavir | 6323 | 0.411 | Novel |
| carprofen | 1636 | 0.409 | Novel |
| sufentanil | 6323 | 0.399 | Novel |
| zafirlukast | 1636 | 0.387 | Novel |
| clidinium | 5241 | 0.386 | Novel |
| eletriptan | 36 | 0.384 | Novel |
| creatine | 36 | 0.383 | Novel |
| bacampicillin | 6530 | 0.368 | Novel |
| fondaparinux | 759 | 0.366 | Novel |
| dihydroergotamine | 3269 | 0.36 | Novel |
| buspirone | 1636 | 0.358 | Novel |
| lipoic | 1636 | 0.344 | Novel |
| dicumarol | 1128 | 0.344 | Novel |
| tolazoline | 36 | 0.344 | Novel |
| gemifloxacin | 6530 | 0.343 | Novel |
| polythiazide | 6530 | 0.343 | Novel |
| letrozole | 4986 | 0.341 | Novel |
| naftifine | 5742 | 0.338 | Novel |
| perphenazine | 4986 | 0.337 | Novel |
| cysteamine | 5241 | 0.334 | Novel |
| voriconazole | 240 | 0.331 | Novel |
| josamycin | 18 | 0.326 | Novel |
| itraconazole | 6833 | 0.325 | Novel |
| metyrapone | 6336 | 0.322 | Novel |
| brimonidine | 781 | 0.321 | Novel |
| riboflavin | 501 | 0.319 | Novel |
| disulfiram | 2099 | 0.319 | Novel |
| a | 36 | 0.318 | Novel |
| vitamin | 36 | 0.316 | Novel |
| paramethasone | 3752 | 0.314 | Novel |
| procaine | 1129 | 0.31 | Novel |
| etoricoxib | 5742 | 0.307 | Novel |
| diphenhydramine | 3781 | 0.304 | Novel |
| dexmedetomidine | 6833 | 0.303 | Novel |
| phenobarbital | 2908 | 0.3 | Novel |
| glycine | 5241 | 0.298 | Novel |
| diatrizoate | 2908 | 0.298 | Novel |
| tolcapone | 1129 | 0.297 | Novel |
| spectinomycin | 1129 | 0.296 | Novel |
| cefradine | 153 | 0.295 | Novel |
| guanadrel | 5241 | 0.287 | Novel |
| dimeglumine | 2908 | 0.283 | Novel |
| l-serine | 18 | 0.282 | Novel |
| glutathione | 2875 | 0.281 | Novel |
| iophendylate | 5742 | 0.279 | Novel |
| acetate | 2555 | 0.277 | Novel |
| cortisone | 1128 | 0.273 | Novel |
| s-adenosylmethionine | 18 | 0.272 | Novel |
| methylbromide | 7153 | 0.27 | Novel |
| guanidine | 3269 | 0.268 | Novel |
| pyridoxal | 6541 | 0.265 | Novel |
| prednicarbate | 2099 | 0.263 | Novel |
| acebutolol | 154 | 0.263 | Novel |
| b | 3751 | 0.26 | Novel |
| procarbazine | 1131 | 0.26 | Novel |
| biotin | 36 | 0.256 | Novel |
| tobramycin | 1129 | 0.248 | Novel |
| levomethadyl | 2555 | 0.247 | Novel |
| miglustat | 7153 | 0.244 | Novel |
| lansoprazole | 2555 | 0.242 | Novel |
| theophylline | 5241 | 0.241 | Novel |
| loracarbef | 6336 | 0.241 | Novel |
| carphenazine | 3751 | 0.238 | Novel |
| acid | 153 | 0.237 | Novel |
| salicylate-sodium | 8854 | 0.237 | Novel |
| thioridazine | 148 | 0.236 | Novel |
| methoxsalen | 3351 | 0.232 | Novel |
| acid | 6240 | 0.231 | Novel |
| altretamine | 1128 | 0.23 | Novel |
| phentermine | 203068 | 0.229 | Novel |
| cyclacillin | 116443 | 0.227 | Novel |
| torasemide | 513 | 0.226 | Novel |
| colchicine | 4985 | 0.224 | Novel |
| rifaximin | 2815 | 0.223 | Novel |
| sulfadoxine | 4986 | 0.221 | Novel |
| nafarelin | 4985 | 0.22 | Novel |
| cladribine | 125 | 0.219 | Novel |
| methotrimeprazine | 125 | 0.219 | Novel |
| crotamiton | 523 | 0.216 | Novel |
| balsalazide | 203068 | 0.216 | Novel |
| timolol | 2555 | 0.214 | Novel |
| betamethasone | 5742 | 0.207 | Novel |
| pyrimethamine | 1128 | 0.206 | Novel |
| conivaptan | 153 | 0.199 | Novel |
| iodipamide | 153 | 0.195 | Novel |
| paromomycin | 3269 | 0.194 | Novel |
| sodium | 2908 | 0.192 | Novel |
| aminophenazone | 1129 | 0.19 | Novel |
| gabapentin | 8854 | 0.189 | Novel |
| succinic | 2875 | 0.187 | Novel |
| oxyphencyclimine | 3752 | 0.187 | Novel |
| capecitabine | 203068 | 0.187 | Novel |
| halazepam | 18 | 0.186 | Novel |
| phenindamine | 5742 | 0.18 | Novel |
| cefamandole | 2561 | 0.178 | Novel |
| decitabine | 4306 | 0.174 | Novel |
| clonazepam | 3269 | 0.172 | Novel |
| fenoldopam | 5241 | 0.166 | Novel |
| rifabutin | 5241 | 0.165 | Novel |
| amiloride | 6716 | 0.163 | Novel |
| acamprosate | 3351 | 0.162 | Novel |
| bacitracin | 196 | 0.16 | Novel |
| alpha-linolenic | 8854 | 0.158 | Novel |
| isoetharine | 5139 | 0.158 | Novel |
| hexachlorophene | 6336 | 0.157 | Novel |
| fluorometholone | 1786 | 0.156 | Novel |
| rocuronium | 3356 | 0.156 | Novel |
| acid | 2099 | 0.155 | Novel |
| metaxalone | 3156 | 0.154 | Novel |
| phenmetrazine | 6336 | 0.153 | Novel |
| acid | 4548 | 0.149 | Novel |
| carbimazole | 3156 | 0.148 | Novel |
| flecainide | 153 | 0.148 | Novel |
| l-valine | 36 | 0.146 | Novel |
| nizatidine | 5241 | 0.146 | Novel |
| polysulfate | 1129 | 0.139 | Novel |
| porfimer | 513 | 0.138 | Novel |
| sulfate | 2904 | 0.138 | Novel |
| trisalicylate-choline | 5241 | 0.138 | Novel |
| cefuroxime | 6531 | 0.134 | Novel |
| benzyl | 1129 | 0.132 | Novel |
| lapatinib | 153 | 0.132 | Novel |
| carbenicillin | 203068 | 0.13 | Novel |
| rifapentine | 6323 | 0.13 | Novel |
| medrysone | 2099 | 0.129 | Novel |
| cilostazol | 5742 | 0.128 | Novel |
| methadyl | 1128 | 0.128 | Novel |
| ergoloid | 6715 | 0.127 | Novel |
| riluzole | 1636 | 0.126 | Novel |
| amrinone | 25 | 0.126 | Novel |
| micafungin | 2908 | 0.124 | Novel |
| zalcitabine | 5742 | 0.123 | Novel |
| vecuronium | 6716 | 0.118 | Novel |
| isopropamide | 6716 | 0.118 | Novel |
| penciclovir | 1956 | 0.117 | Novel |
| glucosamine | 4985 | 0.117 | Novel |
| vorinostat | 3269 | 0.117 | Novel |
| ceftibuten | 1595 | 0.116 | Novel |
| l-tyrosine | 3458 | 0.114 | Novel |
| desoximetasone | 2247 | 0.113 | Novel |
| foscarnet | 759 | 0.112 | Novel |
| thiopental | 2247 | 0.111 | Novel |
| tocainide | 5241 | 0.11 | Novel |
| acid | 5241 | 0.109 | Novel |
| diazepam | 5742 | 0.109 | Novel |
| glibenclamide | 3269 | 0.107 | Novel |
| rifampin | 3269 | 0.107 | Novel |
| monophosphate | 5241 | 0.105 | Novel |
| acid | 2555 | 0.104 | Novel |
| oxtriphylline | 5241 | 0.104 | Novel |
| flunitrazepam | 2555 | 0.104 | Novel |
| mephenytoin | 1129 | 0.102 | Novel |
| fosfomycin | 2099 | 0.102 | Novel |
| budesonide | 1636 | 0.101 | Novel |
| dimethyl | 1786 | 0.1 | Novel |
| anastrozole | 1636 | 0.1 | Novel |
| bezafibrate | 1812 | 0.1 | Novel |
| nitrendipine | 2182 | 0.099 | Novel |
| echothiophate | 1129 | 0.095 | Novel |
| podofilox | 6240 | 0.095 | Novel |
| triethiodide | 4548 | 0.093 | Novel |
| repaglinide | 1128 | 0.092 | Novel |
| gadoteridol | 5465 | 0.091 | Novel |
| gonadorelin | 2561 | 0.091 | Novel |
| propionate | 6531 | 0.091 | Novel |
| sulfoxone | 1129 | 0.089 | Novel |
| cromoglicate | 523 | 0.087 | Novel |
| ertapenem | 6240 | 0.085 | Novel |
| clomocycline | 6476 | 0.084 | Novel |
| mupirocin | 6240 | 0.083 | Novel |
| verteporfin | 5515 | 0.083 | Novel |
| clindamycin | 6833 | 0.083 | Novel |
| triprolidine | 2247 | 0.082 | Novel |
| azacitidine | 2247 | 0.082 | Novel |
| carbetocin | 2247 | 0.082 | Novel |
| melphalan | 759 | 0.081 | Novel |
| acid | 6240 | 0.08 | Novel |
| diphenylpyraline | 4986 | 0.08 | Novel |
| epoprostenol | 84706 | 0.079 | Novel |
| phenoxybenzamine | 36 | 0.078 | Novel |
| pyridoxine | 185 | 0.076 | Novel |
| trimetrexate | 1812 | 0.076 | Novel |
| ethinamate | 2555 | 0.074 | Novel |
| antipyrine | 1583 | 0.074 | Novel |
| thiotepa | 1813 | 0.073 | Novel |
| levofloxacin | 1812 | 0.072 | Novel |
| cephapirin | 4548 | 0.07 | Novel |
| menadione | 3351 | 0.069 | Novel |
| sulfate | 1812 | 0.069 | Novel |
| nelfinavir | 6532 | 0.068 | Novel |
| xanthophyll | 1636 | 0.067 | Novel |
| isradipine | 5733 | 0.066 | Novel |
| oxandrolone | 153 | 0.066 | Novel |
| acenocoumarol | 1131 | 0.066 | Novel |
| amlodipine | 3351 | 0.065 | Novel |
| antrafenine | 1129 | 0.065 | Novel |
| carbamazepine | 5742 | 0.064 | Novel |
| fexofenadine | 2555 | 0.064 | Novel |
| imiquimod | 4986 | 0.063 | Novel |
| acid | 148 | 0.063 | Novel |
| idoxuridine | 6336 | 0.062 | Novel |
| ganciclovir | 2247 | 0.062 | Novel |
| desonide | 148 | 0.062 | Novel |
| cinolazepam | 6716 | 0.062 | Novel |
| stepronin | 4986 | 0.06 | Novel |
| zaleplon | 2555 | 0.059 | Novel |
| l-glutamine | 36 | 0.058 | Novel |
| folic | 5468 | 0.058 | Novel |
| norepinephrine | 2247 | 0.058 | Novel |
| acetyldigitoxin | 1636 | 0.058 | Novel |
| astemizole | 6336 | 0.058 | Novel |
| bexarotene | 36 | 0.056 | Novel |
| tiaprofenic | 153 | 0.056 | Novel |
| phenylephrine | 6329 | 0.054 | Novel |
| epinastine | 4985 | 0.054 | Novel |
| acid | 4548 | 0.054 | Novel |
| topotecan | 2904 | 0.054 | Novel |
| pramipexole | 3612 | 0.053 | Novel |
| miglitol | 1636 | 0.053 | Novel |
| paroxetine | 3612 | 0.053 | Novel |
| molindone | 3612 | 0.053 | Novel |
| pemetrexed | 3156 | 0.052 | Novel |
| isocarboxazid | 5465 | 0.051 | Novel |
| lercanidipine | 4985 | 0.05 | Novel |
| nitrite | 5241 | 0.05 | Novel |
| esmolol | 1128 | 0.049 | Novel |
| gentamicin | 153 | 0.048 | Novel |
| zuclopenthixol | 6541 | 0.048 | Novel |
| cidofovir | 6329 | 0.047 | Novel |
| lauryl | 154 | 0.047 | Novel |
| tioconazole | 6476 | 0.047 | Novel |
| acid | 3351 | 0.046 | Novel |
| halothane | 196 | 0.046 | Novel |
| captopril | 1909 | 0.046 | Novel |
| practolol | 3269 | 0.046 | Novel |
| lisinopril | 1128 | 0.045 | Novel |
| mycophenolic | 4985 | 0.045 | Novel |
| acid | 5241 | 0.029 | Novel |
| olsalazine | 2246 | 0.019 | Novel |
| trimeprazine | 203068 | 0.018 | Novel |
| penicillin | 2908 | 0.017 | Novel |
| dyclonine | 2246 | 0.015 | Novel |
| gallamine | 2246 | 0.014 | Novel |
| sulfoxide | 2246 | 0.014 | Novel |
| sitagliptin | 1128 | 0.014 | Novel |
| didanosine | 146 | 0.012 | Novel |
| acid | 2908 | 0.01 | Novel |
| besylate | 3156 | 0.01 | Novel |
| methylprednisolone | 513 | 0.01 | Novel |
| calcidiol | 2908 | 0.009 | Novel |
| chlormerodrin | 18 | 0.009 | Novel |
| enoxacin | 6240 | 0.008 | Novel |
| dacarbazine | 6531 | 0.008 | Novel |
| minocycline | 2246 | 0.008 | Novel |
| naloxone | 148 | 0.008 | Novel |
| anidulafungin | 1128 | 0.007 | Novel |
| ranitidine | 6240 | 0.006 | Novel |
| tromethamine | 146 | 0.005 | Novel |
| simvastatin | 1813 | 0.005 | Novel |
| etoposide | 6716 | 0.005 | Novel |
| sulfamethazine | 16 | 0.005 | Novel |
| metronidazole | 153 | 0.004 | Novel |
| cinacalcet | 2939 | 0.004 | Novel |
| histamine | 6240 | 0.003 | Novel |
| hydroflumethiazide | 3066 | 0.003 | Novel |
| acid | 2246 | 0.003 | Novel |
| dimenhydrinate | 1636 | 0.003 | Novel |
| pimozide | 7153 | 0.003 | Novel |
| pioglitazone | 2246 | 0.003 | Novel |
| saquinavir | 2246 | 0.003 | Novel |
| alfacalcidol | 1636 | 0.003 | Novel |
| ursodeoxycholic | 1636 | 0.003 | Novel |
| nicotine | 2246 | 0.002 | Novel |
| omeprazole | 38 | 0.002 | Novel |
| carisoprodol | 1128 | 0.002 | Novel |
| violet | 3458 | 0.002 | Novel |
| streptozocin | 240 | 0.002 | Novel |
| cyclothiazide | 153 | 0.002 | Novel |
| nafcillin | 3066 | 0.002 | Novel |
| phosphate | 523 | 0.002 | Novel |
| mofetil | 3269 | 0.002 | Novel |
| acetazolamide | 6240 | 0.002 | Novel |
| ezetimibe | 1812 | 0.002 | Novel |
| gliclazide | 1128 | 0.002 | Novel |
| fomepizole | 1128 | 0.002 | Novel |
| decamethonium | 124 | 0.002 | Novel |
| fencamfamine | 38 | 0.002 | Novel |
| sulfamerazine | 1909 | 0.002 | Novel |
| imipenem | 279 | 0.002 | Novel |
| indecainide | 279 | 0.001 | Novel |
| enflurane | 153 | 0.001 | Novel |
| cetirizine | 1636 | 0.001 | Novel |
| eszopiclone | 153 | 0.001 | Novel |
| teniposide | 240 | 0.001 | Novel |
| albendazole | 16 | 0.001 | Novel |
| imatinib | 513 | 0.001 | Novel |
| furosemide | 6716 | 0.001 | Novel |
| methylsulfate | 2246 | 0.001 | Novel |
| biperiden | 3359 | 0.001 | Novel |
| benzonatate | 1128 | 0.001 | Novel |
| mechlorethamine | 279 | 0.001 | Novel |
| granisetron | 279 | 0.001 | Novel |
| dipyridamole | 4548 | 0.001 | Novel |
| telithromycin | 367 | 0.001 | Novel |
| sulfamethoxazole | 116443 | 0.001 | Novel |
| probenecid | 5241 | 0.001 | Novel |
| perhexiline | 3066 | 0.001 | Novel |
| sibutramine | 4985 | 0.001 | Novel |
| trilostane | 1128 | 0.001 | Novel |
| ambenonium | 4548 | 0.001 | Novel |
| levetiracetam | 2246 | 0.001 | Novel |
| metipranolol | 1128 | 0.001 | Novel |
| finasteride | 1128 | 0.001 | Novel |
| metocurine | 38 | 0.001 | Novel |
| pancuronium | 38 | 0.001 | Novel |
| nitroxoline | 4985 | 0.001 | Novel |
| dextroamphetamine | 153 | 0.001 | Novel |
| iron | 6329 | 0.001 | Novel |
| lincomycin | 16 | 0.001 | Novel |
| adapalene | 1636 | ~ 0 | Novel |
| zolpidem | 4985 | ~ 0 | Novel |
| trimethoprim | 3156 | ~ 0 | Novel |
| quinine | 4985 | ~ 0 | Novel |
| hydroxyzine | 1137 | ~ 0 | Novel |
| sulindac | 150 | ~ 0 | Novel |
| metaraminol | 6240 | ~ 0 | Novel |
| niacin | 150 | ~ 0 | Novel |
| benzoate | 523 | ~ 0 | Novel |
| moexipril | 196 | ~ 0 | Novel |
| scopolamine | 2246 | ~ 0 | Novel |
| tranylcypromine | 1636 | ~ 0 | Novel |
| acetophenazine | 1636 | ~ 0 | Novel |
| ouabain | 116443 | ~ 0 | Novel |
| pergolide | 150 | ~ 0 | Novel |
| estazolam | 1636 | ~ 0 | Novel |
| chlorprothixene | 2892 | ~ 0 | Novel |
| cefoxitin | 3458 | ~ 0 | Novel |
| drospirenone | 3269 | ~ 0 | Novel |
| digitoxin | 279 | ~ 0 | Novel |
| neostigmine | 150 | ~ 0 | Novel |
| acetate | 150 | ~ 0 | Novel |
| phendimetrazine | 38 | ~ 0 | Novel |
| zinc | 1636 | ~ 0 | Novel |
| acid | 150 | ~ 0 | Novel |
| deferasirox | 1128 | ~ 0 | Novel |
| terlipressin | 7153 | ~ 0 | Novel |
| acetic | 148 | ~ 0 | Novel |
